# CySP3-96 Enables Scalable, Streamlined, and Low-Cost Sample Preparation for Cysteine Chemoproteomic Applications

**DOI:** 10.1016/j.mcpro.2024.100898

**Published:** 2024-12-18

**Authors:** Flowreen Shikwana, Beeta S. Heydari, Samuel Ofori, Cindy Truong, Alexandra C. Turmon, Joelle Darrouj, Lara Holoidovsky, Jeffrey L. Gustafson, Keriann M. Backus

**Affiliations:** 1 Biological Chemistry Department, David Geffen School of Medicine, UCLA, Los Angeles, California, USA; 2 Department of Chemistry and Biochemistry, UCLA, Los Angeles, California, USA; 3 Department of Chemistry and Biochemistry, San Diego State University, San Diego, California, USA; 4 Department of Chemistry, Stony Brook University, Stony Brook, New York, USA; 5 Molecular Biology Institute, UCLA, Los Angeles, California, USA; 6 DOE Institute for Genomics and Proteomics, UCLA, Los Angeles, California, USA; 7 Jonsson Comprehensive Cancer Center, UCLA, Los Angeles, California, USA; 8 Eli and Edythe Broad Center of Regenerative Medicine and Stem Cell Research, UCLA, Los Angeles, California, USA

**Keywords:** cysteine, chemoproteomic, SP3, 96-well, covalent, atropisomer

## Abstract

Cysteine chemoproteomic screening platforms are widely utilized for chemical probe and drug discovery campaigns. Chemoproteomic compound screens, which use a mass spectrometry–based proteomic readout, can interrogate the structure activity relationship for thousands of proteins in parallel across the proteome. The versatility of chemoproteomic screens has been demonstrated across electrophilic, nucleophilic, and reversible classes of molecules. However, a key bottleneck that remains for these approaches is the low throughput nature of the most established sample preparation workflows, which rely on many time-intensive and often error prone steps. Addressing these challenges, here we establish a novel workflow, termed CySP3-96, that pairs single-pot, solid-phase-enhanced, sample preparation (SP3) with a customized 96-well sample cleanup workflow to achieve streamlined multiplexed sample preparation. Our CySP3-96 method addresses prior volume limitations of SP3, which allows for seamless 96-well chemoproteomic sample preparation, including for large input amounts that are incompatible with prior methods. By deploying CySP3-96 to screen a focused set of 16 cysteine-reactive compounds, we identify 2633 total ligandable cysteines, including 21 not captured in CysDB. Chemoproteomic analysis of a pair of atropisomeric electrophilic kinase inhibitors reveals striking stereoselective cysteine ligandability for 67 targets across the proteome. When paired with our innovative budget friendly magnetic resin, CySP3-96 represents a versatile, low cost, and highly reproducible screening platform with widespread applications spanning all types of chemoproteomic studies.

## Highlights

• Establish a scalable plate-based SP3 workflow for cysteine chemoproteomics.
• Identify a new low-cost resin for sample cleanup.
• Electrophilic compound screens identify 2633 ligandable cysteines.
• Electrophilic atropisomeric compound pair shows stereoselective ligandability.

## Graphical Abstract

**Figure:**
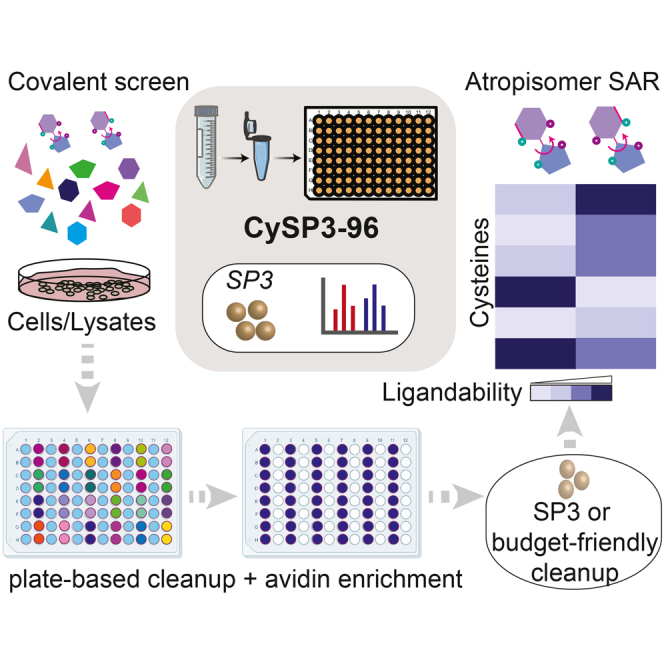

## In brief

Cysteine chemoproteomic methods have enabled the discovery of ligandable or potentially druggable residues proteome-wide. To streamline high-throughput sample preparation for chemoproteomic screens, we developed CySP3-96,which transitions our prior SP3-chemoproteomic workflows into a cost-saving 96-well format, while maintaining comparable performance. By applying CySP3-96 to a screen of a focused set of electrophilic compounds, including custom electrophilic atropisomeric covalent kinase inhibitor analogues, we provide a proteome-wide map of ligandable cysteine residues and demonstrate strong stereoselective cysteine labeling activity for atropisomers.

Mass spectrometry–based chemoproteomics has emerged as a powerful technology capable of pinpointing potential druggable sites proteome-wide. Illustrating the broad impact of chemoproteomics, screening platforms are now available to assess nearly all nucleophilic amino acid side chains (1), inclusive of cysteine (2, 3, 4, 5), lysine (6, 7), serine (8, 9, 10), tyrosine (11, 12, 13), methionine (14, 15), glutamate, and aspartate (16, 17, 18). Cysteine remains a favored residue to target, due to its high nucleophilicity, important structural and functional roles within proteins, and proven therapeutic relevance, as exemplified by FDA-approved cysteine-reactive drugs such as the Gly12Cys GTPase KRas inhibitors (19, 20). Thus, a key objective of cysteine chemoproteomic studies is to enable the discovery of scout or pathfinder molecules (3, 21, 22, 23, 24, 25, 26, 27, 28, 29, 30, 31, 32, 33, 34, 35, 36, 37) for all potentially druggable, or “ligandable,” cysteine residues.

Towards achieving this goal, chemoproteomic screening platforms apply the same general strategy. First, cells or lysates are treated with electrophilic compounds or vehicle, followed by capping of all unreacted cysteines with a pan-cysteine reactive probe, such as iodoacetamide alkyne (IAA) or iodoacetamide desthiobiotin. After control and treatment groups are isotopically differentiated, either *via* heavy/light biotin-reagents (5, 38) or with isobaric tags (21, 39, 40), samples are then subject to sequence-specific proteolysis and enrichment on avidin resin along with multiple sample cleanup steps throughout this process. Compound-modified cysteines are then identified *via* liquid-chromatography tandem mass-spectrometry (LC-MS/MS) based on compound-induced decreases in either precursor or reporter ion abundance. Demonstrating their broad utility, these workflows have now been widely implemented both by our group (3, 38, 40, 41, 42, 43) and many others (4, 21, 39, 44), enabling discovery of covalent tool compounds that engage a wide range of protein classes, spanning transcription factors (45), RNA binding proteins (35), kinases (36), proteasome regulators (34), proteases (3, 46), and even classes of posttranslationally modified cysteines (47).

One important consideration for chemoproteomic screens is library composition. As chemoproteomic screens remain comparatively low throughput, selection of a focused and diverse set of electrophilic fragments and more elaborated compounds has become a go-to strategy to maximize the coverage of potential targets (2, 44). Alongside structural diversity, the addition of enantioenriched libraries to chemoproteomic screening decks provides added value to facilitate delineation of high confidence compound–cysteine interactions and on target phenotypes (28, 34, 45, 48, 49, 50, 51). While a number of studies have now reported chemoproteomic datasets using pairs of enantioprobes, the target profile of compounds featuring other types of chirality remains largely unexplored. Atropisomerism is a conformational chirality that occurs when there is constrained rotation about a bond that results in the ‘rotamers’ being isolable enantiomers. Atropisomerism is becoming increasingly ubiquitous throughout modern drug discovery (52). However, to the best of our knowledge, there have been no chemoproteomic studies for electrophilic atropisomer pairs.

With the increasing availability of comparatively large 3200+ electrophilic compound libraries available for purchase and new straightforward chemistries for rapid electrophilic compound library synthesis (53), the number of easily accessible electrophilic compounds available to researchers far outstrips current throughput of chemoproteomic screening platforms, both in terms of sample preparation and data acquisition. Methodological innovations that result in streamlined sample preparation offer the potential to improve sample throughput. Many groups have developed approaches to address these issues, such as SP2 for rapid and reproducible proteomics (54), implementation of microflow LC-MS/MS analysis for improved reproducibility (55), development of intelligent acquisition software (56), and utilizing a label-free approach to speed up acquisition and throughput (57). Transitioning from 1.5 ml microfuge tubes to 96-well plates is one approach to improve sample reproducibility and throughput, which allows for reduced manual sample manipulation, decreased points for human error, and compatibility with automation. Sample cleanup methods, such as in-stage tip (58), S-Trap (59), and single-pot, solid-phase-enhanced sample preparation (SP3) (60, 61) are integral parts of most plate-based sample workflows, as illustrated by their applications to phosphoproteomics (62), clinical proteomics (63, 64), identification of stereo-specific targets (45), and discovery of off target proteins (39). Our recent work revealed that SP3 cleanup is well-suited to chemoproteomics, yielding improved coverage and benefitting from decreased input material (41, 42). SP3 cleanup is also featured in the recently reported TMT- and 96-well-plate–based chemoproteomic platform by Gygi *et al.* (21) and has been shown to have high reproducibility between experimental replicates (65), which further demonstrates the utility of SP3 in transitioning cysteine chemoproteomics to streamlined automated sample preparation.

Despite these considerable advances, several unaddressed challenges remain. First, as demonstrated by us and others (41, 66), SP3 cleanup does not scale well, with decreased yields at larger input materials—this limitation is particularly relevant for less reactive chemical probes, for which more sample is required to achieve higher coverage and for compatibility with microflow acquisition methods that are distinguished by lower failure rates than nanoflow (55, 67). Second, and looking beyond SP3-specific workflows, many cleanup methods are quite costly, hindering implementation for large-scale screening applications. And lastly, for chemoproteomics, the avidin-enrichment step remains a bottleneck for fully transitioning an entire workflow into a 96-well format, with most prior reports conducting peptide capture manually in plastic containers.

Here we report the CySP3-96 method, which implements an unprecedented scalable SP3 cleanup and a low cost SP3-alternative resin to enable robust and high coverage cysteine ligandability screens in a fully 96-well plate format. To establish CySP3-96, we first determined the source of the decreased coverage for SP3 samples generated with higher protein input, namely increased peptide concentration in the tryptic digest. We addressed this limitation by bypassing the SP3 peptide cleanup step entirely, which allowed us to maintain volumes compatible with deep-well plates. Benchmarking the CySP3-96 method to our established cysteine-SP3 platform revealed comparable coverage together with at least a 90-min per sample decrease in processing time with increasing time saved proportional to increase in the number of samples. These improvements allowed for high coverage screening in a 96-well plate format of 16 total known and novel electrophiles, including unprecedented atropisomeric compounds, which identified 2633 total ligandable cysteines, of which 21 have not been reported previously in CysDB (68). Distinguished by scalability, low cost, and ease of implementation, we expect CySP3-96 to prove widely useful for a broad range of cysteine chemoproteomic applications.

## Experimental Procedures

### Cell Lines, Culture Conditions

Cells were cultured in Roswell Park Memorial Institute (Gibco RPMI 1640 Medium, 11875119) 1640 media supplemented with 1% Penicillin-Streptomycin (Gibco, Penicillin-Streptomycin 10,000 U/ml, 15140122) and 10% fetal bovine serum (Avantor, Seradigm, Premium Grade Fetal Bovine Serum, Cat. No. 97068-085). The fetal bovine serum used with our cell culture media was obtained from Avantor Seradigm (lot #214B17). The Jurkat cell line was obtained from ATCC. Cells were grown at 37 °C and 5% CO_2_ in a humidified incubator. Cell line was tested monthly with the *Mycoplasma* detection kit (InvitroGen).

### Cell Harvesting and Lysis

Cells were centrifuged at 300*g* for 5 min and media was aspirated. Cells were washed twice with cold 1X PBS twice. Cells were resuspended in cold 1X PBS prior to lysis which was done by probe sonication on ice (1 s of sonication at 2 amplitude with 10 s of rest on ice for a total of 10 cycles). Lysates were clarified by centrifugation at 13,000*g* for 5 min and the supernatant transferred to a new cold microfuge tube. The protein concentration of the lysate was determined by BioRad DC protein assay kit (Cat. No. 5000112 BioRad Life Science). Lysates were then diluted to stated concentrations for experiments.

### IA-Rhodamine Gel

Jurkat whole cell lysates were normalized to 1.5 mg/ml. Each lysate was treated with either DMSO or compound (500 μM or at stated concentration) for 1 h at ambient temperature. Treatment was followed by labeling with 5 μM of IA Rhodamine for 20 min at ambient temperature. Then, 4× SDS loading dye was added to each sample at a final concentration of 1×. Samples were then loaded into a BioRad Bis/Tris gel and run until the dye front ran off at 150V. Gel was imaged with a BioRad ChemiDoc using the Rhodamine channel. Coomassie (Abcam InstantBlue Coomassie Protein Stain, Cat. No. ab119211) was obtained by rocking gel with Coomassie overnight at 4 °C and washing gel with MilliQ water. Gel was imaged with Coomassie gel setting on the BioRad ChemiDoc.

### Cysteine Chemoproteomic Sample Preparation Using Biotin-Azide Capture Reagents, TEV-Cleavable Biotin-Azide Tags, or sCIP Biotin Capture Reagents

Jurkat whole cell lysates were normalized to either 1.5 mg/ml or 2 mg/ml (as stated). Each lysate was treated with either DMSO or compound (500 uM or at stated concentration) for 1 h at ambient temperature. Treatment was followed by labeling with 200 μM IAA for 1 h at ambient temperature. Then, samples were subjected to bioorthogonal copper(I)-catalyzed azide-alkyne cycloaddition ‘click’ conjugation to heavy (DMSO treated) or light (compound treated) biotin azide tags at ambient temperature for 1 h. The click reaction was performed for 200 μl sample volumes by adding 24 μl of a premixed solution of click reagents which included TBTA (12 μl of 1.7 mM stock in DMSO/t-butanol 1:4 per sample, 100 μM final concentration), CuSO_4_ (4 μl of 50 mM stock in water per sample, 1 mM final concentration), TCEP (4 μl of fresh 50 mM stock in water per sample, 100 μM final concentration), and biotin azide (for biotin-azide and silane-based cleavable isotopically labeled proteomic (sCIP) capture reagents: 4 μl of 20 mM stock in DMSO per sample, 400 μM final concentration; for tobacco etch virus (TEV) tags: 4 μl of 5 mM stock per sample, final concentration = 100 μM). After click, protein was solubilized by the addition of SDS at a final concentration of SDS. Samples were then treated with benzonase (Novagen Benzonase Nuclease, Purity >90% MilliporeSigma 707464) at 37 °C for 30 min. Heavy and light samples were combined at equal volumes and subjected to cleanup.

### Protein Cleanup Using SP3

Sera-Mag SpeedBeads Carboxyl Magnetic Beads (GE Healthcare, 65152105050250, 50 μg/μl, total 1 mg and GE Healthcare, 45152105050250, 50 μg/μl, total 1 mg) were combined in equal volumes for a total of 60 to 80 μl per sample. The bead slurry was washed 3 times with ultrapure water and resuspended in 60 to 80 μl of water per sample. The washed beads were then added to each sample (600–800 μg protein input) and incubated at ambient temperature with shaking (1000 rpm) for 5 min. Six hundred microliters of absolute ethanol (for ≥55% ethanol v/v) was added to each sample and samples were then incubated with shaking (1000 rpm) for an additional 5 to 10 min at ambient temperature. Beads were then separated from the solution using a magnetic rack (Sergi Lab Supplies, Cat. No. 1005a) and washed 3 times using 80% ethanol. Beads were then resuspended in 200 μl of 2M urea in PBS with 0.5% SDS. DTT was then added at a final concentration of 10 mM and samples were incubated at 65 °C for 15 min. Following this, IA was added at a final concentration of 20 mM and samples were incubated for 30 min at 37 °C. Then, 500 μl of absolute ethanol was added to each sample and incubated at ambient temperature with shaking (1000 rpm) for 5 to 10 min. A magnetic rack was again used to separate the beads and washed 3 times with 80% ethanol. The beads were then resuspended in 150 μl of 2M urea in PBS containing 4 mM CaCl_2_ and 2 μl of 1 mg/ml trypsin (Worthington Biochem LS003740). Samples were digested at 37 °C with shaking (300 rpm) for 12 to 16 h.

### Peptide Cleanup Using SP3

**Fig. 1:**
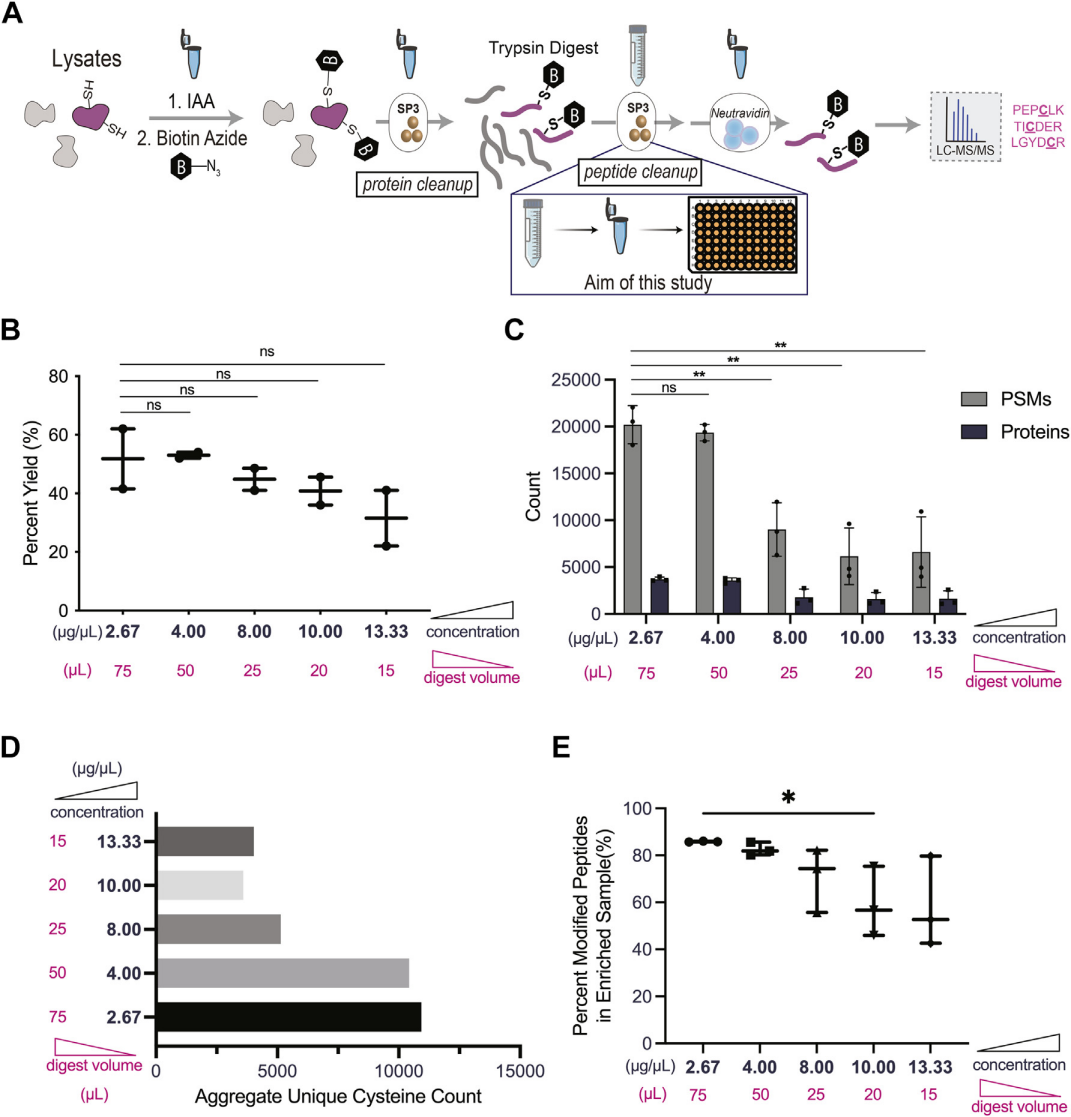
**SP3 peptide cleanup volume scaledown results in overall decreased coverage.***A*, shows v1.0 cysteine chemoproteomics workflow in which SP3 resin is used for both protein- and peptide-level sample cleanup, with scaledown of trypsin digestion sample volume to achieve compatibility with 96-well plate. *B*–*E*, 200 μg of Jurkat whole lysate capped with IAA and biotin azide and subjected to v1.0 cysteine chemoproteomic sample preparation varying the trypsin digest volume and concentration. *B*, calculated peptide recovery using peptide assay varying digest concentration and digest volume. n = 2. *C*, peptide spectral matches (PSMs) and protein coverage for digest volume scaledown. n = 3. *D*, counts of unique cysteines across all replicates (n = 3) in aggregate for each digest volume used for sample preparation. *E*, percent modified peptides in comparison to all detected peptides for each digest concentration. n = 3. For *B*, *C*, *E*, data represent mean values and SD. Statistical significance was calculated with unpaired Student's t-tests, ns, not significant, ∗ *p* < 0.05, ∗∗ *p* < 0.005, NS *p* > 0.05. All MS data is available in Supplemental Table S4.

Acetonitrile was added to the tryptic peptides and SP3 beads at ≥95% of the final volume (3.8 ml acetonitrile for the 150 μl digest solution; refer to Supplemental Table S1 for exact volumes corresponding to Fig. 1) and incubated at ambient temperature for 10 min with shaking (1000 rpm). The beads were then separated from the solution using a magnetic rack and washed 3 times with 1 ml of 100% acetonitrile. The beads were then resuspended in 100 μl of 2% DMSO in ultrapure water and incubated at 37 °C with shaking (1000 rpm) for 30 min. This elution was collected in a separate low-bind tube (Fisherbrand, Low-Retention Microcentrifuge Tubes Cat. No. 02-681-320) and the beads were resuspended in an additional 100 μl of 2% DMSO in ultrapure water at the same conditions as the first elution, except for 45 min. The second elution was collected and combined with the first elution.

### Peptide Cleanup Through Neutravidin Enrichment

Note: Neutravidin enrichment was used for the isotopically labeled biotin azide enrichment reagents which have no cleavable moiety. These reagents were used primarily throughout this work unless otherwise stated. For each sample, 50 μl of agarose neutravidin beads (Thermo Fisher Scientific 29200) were washed 3 times with IAP buffer (50 mM MOPS-NaOH, 10 mM Na_2_HPO_4_, 50 mM NaCl, pH 7.2), then resuspended in a slurry of 400 μl of IAP buffer per sample. The 400 μl neutravidin bead slurry was added to each sample of tryptic peptide (as obtained through the method described above “Protein cleanup using single-pot solid-phase-enhanced sample preparation (SP3)”) and let incubate for 2 h at ambient temperature (for microfuge tubes, samples were rotated; for plate, samples were left shaking at 600 rpm). The 2 M urea digest samples were diluted 4-fold during this step. Then, beads were centrifuged at 1800*g* for 3 min and the supernatant was aspirated. The beads were then washed 3 times with PBS followed by another three washes with ultrapure water. The beads were then resuspended in 60 μl of 80% acetonitrile in water with 0.1% formic acid to elute peptides from beads. This first elution was incubated at ambient temperature for 10 min. Beads were separated by centrifugation (1800*g* for 3 min) and the elution was collected in a new low-bind microfuge tube. The beads were again resuspended in another 60 μl of the elution solution and let incubate at 72 °C for 10 min. The second elution was collected and combined with the first. The beads were once again resuspended with 60 μl of the same elution solution and this last elution was also collected and combined with the prior elutions. Samples were then dried (SpeedVac) and reconstituted with mass spectrometry buffer (5% acetonitrile and 1% formic acid in ultrapure water) and analyzed by LC-MS/MS.

### Protein Cleanup in a 96-Deep Well Plate Using SP3 Beads

Samples were prepared in the same way as described in the methods above “Isotop-ABPP sample preparation” and “Protein cleanup using SP3” with the following exceptions: All lysates were aliquoted into each well of a 96-deep well plate (MilliporeSigma Supelco SPE 96-Deep Square Well Collection Plate, Cat. No. 11-100-3690) and all treatments and click were carried out within the wells of the plate. Plate was sealed with a sealing mat (MilliporeSigma Supelco 96 Square Well Pierceable Cap Mats, Cat. No. 11-100-3884) during each incubation. The magnetic beads were separated with a magnetic stand-96 apparatus (Thermo Fisher Scientific, Cat no. AM10027). Beads were resuspended within the wells of the plate by brief (30 seconds-1 minute) shaking at 1000 rpm on an Eppendorf ThermoMixerC (Cat. No. 5382000023) with plate attachment (Eppendorf Smartblock Thermoblock Cat. No. 5363000039). Multichannel pipettes were used for the addition of lysates and reagents to the wells. All volumes were kept under 1.1 ml within the wells.

### Peptide Cleanup in a 96-Deep Well Plate Using SP3 Beads

Digest solutions post-digest were transferred into a new 96-deep well plate and diluted 2 fold with PBS. For each sample, 50 μl of magnetic neutravidin beads (Cytiva Cat No. 78152104010150) were washed 3 times with IAP buffer (50 mM MOPS-NaOH, 10 mM Na_2_HPO_4_, 50 mM NaCl, pH 7.2) and then resuspended in a slurry of 300 μl of IAP buffer per sample. Plate was then sealed with a sealing mat. Samples were incubated with the neutravidin beads for 2 h at ambient temperature with shaking at 1000 rpm on the thermomixer with plate attachment. Beads were then separated using the magnetic stand-96 apparatus and washed 3 times with PBS followed by another three washes with ultrapure water. Beads were then resuspended in 60 μl of 80% acetonitrile in water with 0.1% formic acid for the elution of peptides at ambient temperature for 10 min. For resuspension of beads, plate was subjected to brief (30 seconds-1 min) of shaking at 1000 rpm on plate shaker. Three total elutions were performed and collected as described in the section “Peptide cleanup through neutravidin enrichment” with the modifications of the incubations done with mixing at 1000 rpm and the 72 °C incubation done on the thermomixer with an Eppendorf ThermoTop (Cat. No. 5308000003). Samples were then vacuum dried (SpeedVac) and reconstituted with mass spectrometry buffer (5% acetonitrile and 1% formic acid in ultrapure water) and analyzed by LC-MS/MS.

### Peptide Cleanup Through Streptavidin Enrichment

Note: Streptavidin enrichment used for the following biotin capture reagents: TEV cleavable isotopic Tandem Orthogonal Proteolysis-ABPP (isoTOP-ABPP)-TEV biotin azide reagents and sCIP biotin azide reagents containing acid cleavable dialkoxydiphenylsilane moiety. These reagents were used to establish compatibility of method with streptavidin enrichment as well as neutravidin enrichment. For each sample, 50 μl of agarose streptavidin beads (Thermo Fisher Scientific 20353) were washed 3 times with PBS and then resuspended in a slurry of 400 μl of beads in PBS per sample. The 400 μl neutravidin bead slurry was added to each sample of tryptic peptide (as obtained through the method described above “Protein cleanup using single-pot SP3”) and let incubate for 2 h at ambient temperature (for microfuge tubes, samples were rotated; for plate, samples were left shaking at 600 rpm). The 2 M urea digest samples were diluted 4-fold during this step. Then, beads were centrifuged at 1800*g* for 3 min and the supernatant was aspirated. The beads were then washed three times with PBS followed by another three washes with ultrapure water. For isoTOP-ABPP-TEV (5) reagents, beads were resuspended in TEV buffer (50 mM Tris, pH 8, 0.5 mM EDTA, 1 mM DTT) with 1 μl TEV protease (2 mg/ml; MacroLab) and left rotating in microfuge tubes overnight. Elution was collected by centrifugation of beads (1800*g* for 3 min) followed by collection of supernatant, then one 20 μl wash of the beads followed by another round of centrifugation and collection. Elutions were then zip-tipped using Pierce C18 tips (Thermo Fisher Scientific 87784) according to the manufacturer's protocol. Samples were then dried (SpeedVac) and reconstituted with mass spectrometry buffer (5% Optima LC/MS grade acetonitrile and 1% Optima LC/MS formic acid in ultrapure water) and analyzed by LC-MS/MS.

For MS1 sCIP reagents, the beads were resuspended in 200 μl of 2% formic acid in water and the elution was incubated at ambient temperature for 30 min. Beads were separated by centrifugation (1800*g* for 3 min) and the elution was collected in a new low-bind microfuge tube. A second elution was carried out with 80% acetonitrile in water for 5 min at ambient temperature. The second elution was collected and combined with the first. Samples were then dried (SpeedVac) and reconstituted with mass spectrometry buffer (5% Optima LC/MS grade acetonitrile and 1% Optima LC/MS formic acid in ultrapure water) and analyzed by LC-MS/MS.

### Protein Cleanup Using Standard DNA Cleanup Magnetic Beads

DNA cleanup magnetic beads obtained from SergiLabSupplies (Cat. No. 1040, Lot S22122801) were used at the stated volumes of bead slurry. The exact procedures for protein cleanup with SP3 beads were followed.

### Peptide Cleanup Using Standard DNA Cleanup Magnetic Beads

The exact procedures for peptide cleanup with SP3 beads were followed.

### Measurement of Peptide Concentrations

Peptide concentrations were obtained through the use of the Pierce Quantitative Colorimetric Peptide Assay (Thermo Fisher Scientific, Cat no. 23275). Manufacturer's protocol was followed.

### Assessing Bead Concentration and Size

A Thermo Fisher Countess II was used to measure bead concentration and size for each bead type. Beads were diluted as stated and the final reported count was adjusted based on the initial dilution.

### LC-MS/MS Acquisition

The samples were analyzed by LC-MS/MS using a Thermo Scientific Orbitrap Eclipse Tribrid mass spectrometer. Peptides were fractionated online using an 18 cm long, 100 μM inner diameter (ID) fused silica capillary packed in-house with bulk C18 reversed phase resin (particle size, 1.9 μm; pore size, 100 Å; Dr Maisch GmbH). The 70-min or 120-min water/acetonitrile gradient was delivered using a Thermo Scientific EASY-nLC 1200 system at different flow rates (buffer A: water with 3% DMSO and 0.1% formic acid and buffer B: 80% acetonitrile with 3% DMSO and 0.1% formic acid). Detailed gradient information for each experiment is outlined in Supplemental Table S3. Data was collected with charge exclusion (1, 8, >8). Data was acquired using a data-dependent acquisition method consisting of a full MS1 scan (Resolution = 120,000) followed by sequential MS2 scans (Resolution = 15,000) to utilize the remainder of the 1 s cycle time. Precursor isolation window was set as 1.6 and normalized collision energy was set as 30%. Files of MS data with corresponding experiments can be found in Supplemental Table S8.

### Methods for Covalent Docking

The covalent docking function of Molecular Operating Environment (MOE) 2020 was used to dock BEH-1 into a cocrystal structure of serine/threonine-protein kinase Chk2 (CHEK2) (PDB ID: 2XBJ) using the Amber10:EHT forcefield. The protein was first prepared using the “QuickPrep” function in MOE with its default settings, and BEH-1 was put in the correct protonation state at pH 7. Cys231 was identified in the crystal structure and was used to define the active site of CHEK2. Solvent atoms were not taken into account during the docking. The reaction that was used to generate the covalent bond was “Michael acceptor, 1,4-addition, beta-mercapto carbonyl,” which was picked among the list of reactions MOE provided (Functional group, Class, and Product areas respectively). The induced fit postplacement refinement method was used, in which the ligand was allowed to move ∼6 Å away from the defined binding site, and the side chains were free to move. The final scoring methodology used was the Generalized Born Volume Integral/Weighted Surface Area dG. The top five scoring (“S score” or “MOE predicted affinity value” on MOE) nonredundant poses were retained. Each pose was further minimized using the “Minimize” function on MOE to obtain the final docked structures.

### Data Compilation and Statistics

**Fig. 2:**
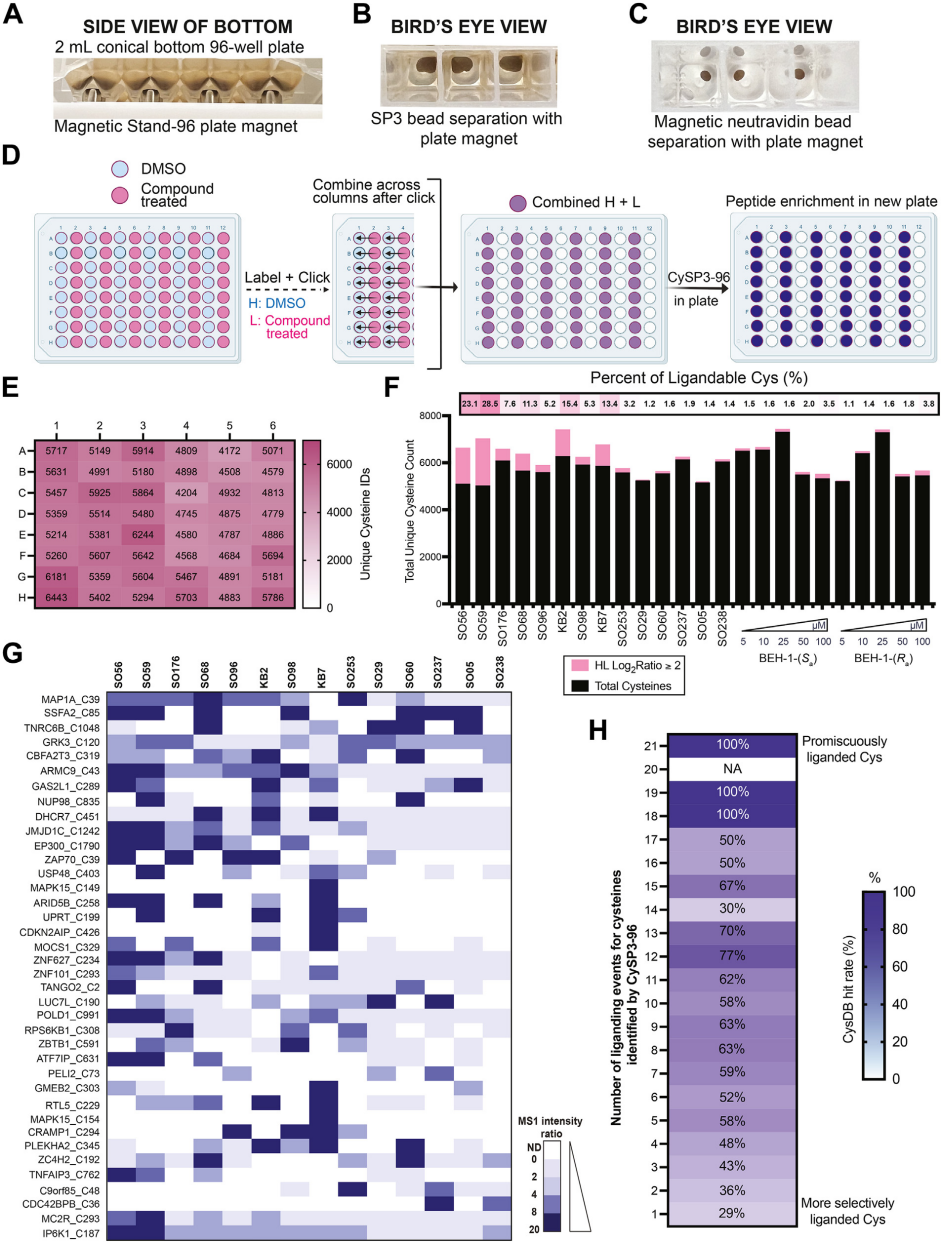
**Streamlined SP3 preparation seamlessly transitions into 96 deep-well plate format for the screening of cysteine reactive electrophiles.***A*, deep-well plate with Magnetic Stand-96 magnet used for bead separation. *B*, visualization of the magnetic bead separation in each well. *C*, visualization of Cytiva magnetic neutravidin bead separation in each well for enrichment. *D*, workflow for the plate-based screening starting with in-plate lysate treatment with compounds or vehicle, labeling with IAA, click conjugate to either heavy or light biotin azide enrichment reagents, and proteomic sample preparation. *E*, number of unique cysteines identified per well across the plate for cysteine electrophilic compound screen. *F*, cysteine ligandability per compound as compared to overall coverage. *G*, shows the SAR of liganded cysteines not previously identified in CysDB. MS1 intensity ratios are calculated as H/L, with N.D. indicating that the cysteine was not detected. *H*, comparison of the hit rate (percentage of total compounds screened that engage a cysteine in CysDB) to the number of liganding events reported by CySP3-96, with a max of 21 liganding events for the 16 compounds screened, including dose response assessments. For panels *E–H*, Jurkat whole cell lysate (300 μg) in 96-well plate treated with either compound (500 μM unless otherwise stated) or vehicle followed by IAA (200 μM), clicked to either light or heavy biotin-azide, and processed *via* chemoproteomics workflow v2.0, n = 2 per condition. All MS data is available in Supplemental Table S6.

Raw data collected by LC-MS/MS were searched with MSFragger and FragPipe (version 19.0) (69, 70, 71). MSFragger isoDTB-ABPP workflow, with the following parameters and all other settings as default. Protease was set to strict trypsin with cut sites at K and R with no cuts if P prior. Precursor and fragment mass tolerance was set as 20 ppm. Missed cleavages were allowed up to 2. Peptide length was set 7 to 50 and peptide mass range was set 500 to 5000. MS1 intensity ratio of heavy and light labeled cysteine peptides and compiled for individual proteins were reported. Chemoproteomics datasets were searched with variable modifications for both carbamidomethylation (+57.02146), and for biotin azide reagents, MS1 labeling quant was enabled with light set as C+463.2366 and heavy set as C+469.2742. For isoTOP-ABPP experiments with TEV reagents, MS1 labeling quant was enabled with light set as C+521.3074 and heavy set as C+527.3213. For multicysteine-containing peptides, MSFragger modification localization sites were reported (72). For chemoproteomic experiments with sCIP reagents, MS1 labeling quant was enabled with light set as C+493.3007 and heavy set as C+499.3145. MS1 intensity ratio of heavy- and light-labeled cysteine peptides was reported. Peptide and protein level FDR were set to 1%. Database searches were conducted against a reverse concatenated UniProtKB Human database corresponding to the 18432 CCDS release with a release date of 01-01-2020 with 18,555 entries and 18,555 decoys. Calibrated and deisotoped spectrum files produced by FragPipe were retained and reused for this analysis. RAW files were searched for initial coverage studies with MSConvert (73, 74) used to generate .mzML files for ratio-based analysis with IonQuant (71). For experiments utilizing MS1 quantitation (*e.g.*, isoTOP-ABPP), custom python scripts were implemented to compile labeled peptide and protein datasets. Unique proteins, unique cysteines, and unique peptides were quantified for each dataset. Unique proteins were established based on UniProtKB protein IDs. Unique peptides were found based on sequences containing a modified cysteine residue. Unique cysteines were classified by an identifier consisting of a UniProtKB protein ID and the amino acid number of the modified cysteine (ProteinID_C#); residue numbers were found by aligning the peptide sequence to the corresponding UniProt protein sequence. When there are multiple cysteines in one peptide, all the cysteine residue numbers will be reported as ProteinID_C#_C# though it is undistinguishable on which cysteine the modification is. Cysteine residue identifiers in this format were compared to CysDB v1.5 (68) where stated. Ligandability was assessed based on Log_2_(H/L) ratios of ≥2 unless otherwise stated. The total ligandable cysteines stated excluded cysteine residues liganded by SO56 and SO59 due to their high proportion of “ligandable” cysteines (*i.e.*, apparent high reactivity). For Figure 2*H* analysis, percentages report the fraction of CysDB ligandable cysteines out of the total cysteines liganded by each condition (21 total conditions). Higher percentages indicate more promiscuously liganded compounds in CysDB. Statistical values including the exact n, statistical test, and significance are reported in the Figure Legends. Statistical significance was defined as *p*-value < 0.05 and unless indicated otherwise determined by an unpaired 2-tailed Student’s *t* test.

### Statistics

Statistical analysis was performed using GraphPad Prism (v10.1.0) for Mac (GraphPad Software). Statistical values including the exact n, statistical test, and significance are reported in the Figure Legends. Statistical significance was defined as *p*-value < 0.05 and unless indicated otherwise determined by an unpaired 2-tailed Student’s *t* test.

### Experimental Design and Statistical Rational

**Fig. 3:**
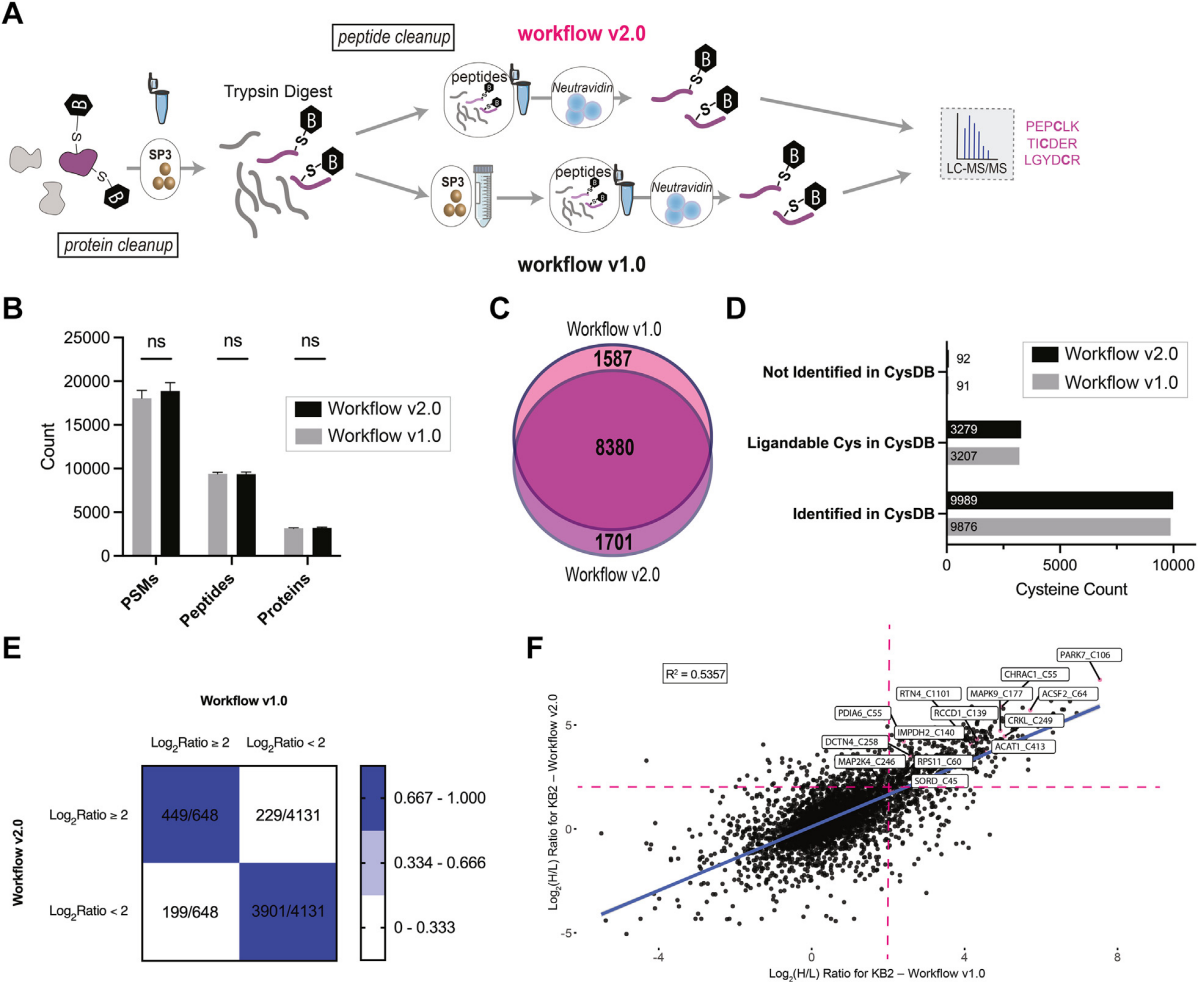
**Skipping peptide cleanup results in comparable sample coverage and a more streamlined sample enrichment.***A*, shows comparison of v1.0 to v2.0 workflows, with the latter omitting SP3 peptide cleanup post-digestion. Instead, in v2.0, the tryptic digest is immediately enriched onto avidin beads without extra cleanup steps. For the head-to-head comparison of workflows v1.0 and v2.0, the digest volumes were maintained at 150 μl. *B*–*D*, Jurkat whole cell lysate (400 μg) in microcentrifuge tubes labeled with IAA (200 μM) and clicked to biotin-azide processed using either workflow. n = 3 per condition. *B*, comparison of labeled PSM, peptide, and protein coverage between workflow v1.0 and workflow v2.0. *C*, aggregate unique and shared cysteines across three replicates for each workflow. *D*, comparison of coverage to CysDB, including both identified and ligandable unique cysteines. *E* and *F*, Jurkat whole cell lysate (400 μg) in microcentrifuge tubes treated with either KB2 (500 μM) or vehicle followed by IAA (200 μM), clicked to biotin-azide, and processed using either workflow, n = 4 per condition. *E*, concordance plot comparing distribution of Log_2_(H/L) ratios for workflows v1.0 and 2.0 for KB2-treated Jurkat whole cell lysates. Cysteine identifiers found in more than one replicate in both workflows considered. *F*, concordance of ligandability ratios for each workflow for KB2-treated Jurkat whole cell lysates with highlighted known KB2 liganded cysteines. *Pink dashed lines* indicate threshold for cysteine ligandability (Log_2_ HL ratio ≥2). Cysteine identifiers found in more than 1 replicate in both workflows considered. For *B*, data represent mean values and SD. Statistical significance was calculated with unpaired Student's t-tests, ns, not significant *p* > 0.05. All MS data is available in Supplemental Table S5.

**Fig. 4:**
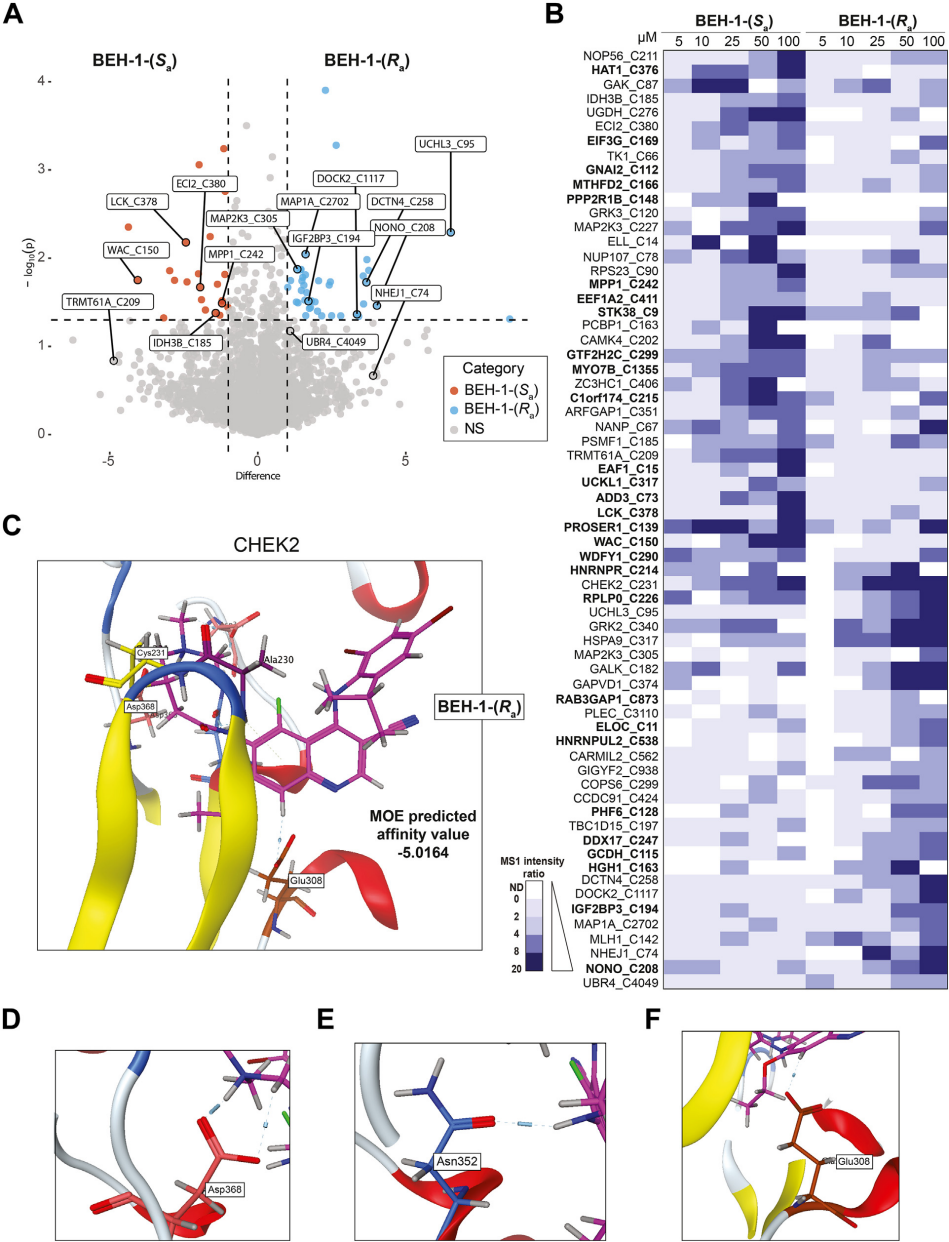
**Concentration screen of two atropisomers BEH-1-(*S***_**a**_**) and BEH-1-(*R***_**a**_**) illustrates stereoselectivity across targets.***A* and *B*, Jurkat whole cell lysate (300 μg) in 96-well plate treated with either BEH-1-(*S*_a_) and BEH-1-(*R*_a_) at the indicated concentration or vehicle followed by IAA (200 μM), clicked to either light or heavy biotin-azide, and processed *via* chemoproteomics workflow v2.0, n = 2 per condition. *A*, volcano plot of the fold change difference of the Log_2_(H/L) ratios for Jurkat whole cell lysates treated with BEH-1-(*S*_a_) and BEH-1-(*R*_a_) (100 μM) followed by cysteine chemoproteomic analysis. *B*, heatmap of atropisomer targets across a range of concentrations between 5 and 100 μM. Bolded sites indicate those not previously identified as ligandable in CysDB. *C*, CHEK2 (PDB ID: 2XBJ) Cys231 docked to BEH-1-(*R*_a_) with an MOE-predicted affinity value of −5.0164. Four out of five poses observed with BEH-1-(*R*_a_) atropisomer. *D*, a zoom in of *C*: Asp368 in the DFG motif of CHEK2 making hydrogen bonding interactions with the dimethylamino moiety of BEH-1-(*R*_a_) as well as its adjacent CH_2_ group. *E*, a zoom in of *C*: Asn352 in the hinge region of CHEK2 making a hydrogen bond with the hydrogen of the amide group in the covalent linker moiety of BEH-1-(*R*_a_). *F*, a zoom in of *C*: Glu308 in the hinge region of CHEK2 making a hydrogen bond with the C8 hydrogen of the quinoline moiety of BEH-1-(*R*_a_). All docking figures generated with CHEK2 PDB structure 2XBJ in MOE 2020. All MS data is available in Supplemental Table S6. CHEK2, serine/threonine-protein kinase Chk2.

**Fig. 5:**
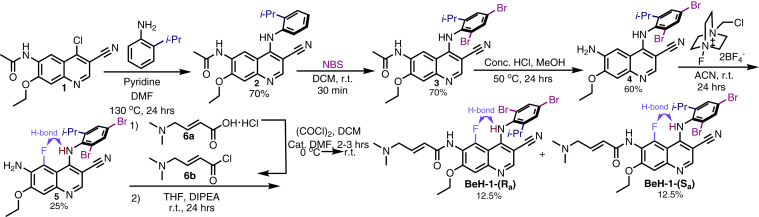
Synthesis of atropisomeric compounds.

All experiments were performed with at least n = 2 replicates. Figure 1*B* consisted of two replicates and Figure 1, *C–E* as well as Figure 3, *B–D* consisted of three replicates which was deemed sufficient for the assessment of our modified workflow and its effect on peptide/protein/cysteine coverage. For Figures 1, *C–E* and 2, *B–D*, a student’s unpaired *t* test was used to determine statistical significance, where applicable. Controls employed within these experiments are methodological controls with already published and established workflows performed side-by-side with the newly modified workflow as a benchmark. The experiment corresponding to Figure 3, *E* and *F* consisted of n = 4 replicates which was deemed necessary for assessing the reliability of the ratios obtained from both workflows in the context of electrophilic fragment screening. For the assessment of ratio correlation, only cysteines identified in two or more replicates in both experiments were considered for assessing correlation as to increase the confidence in the data. For experiments corresponding to the plate screen in Figures 2 and 4, each compound was screened in duplicate with a DMSO control in each H/L pair. These should be considered biological replicates as the lysate treatments were all done separately. The filtering criteria for these datasets to assess ligandability was based on Log2(H/L) ratios of ≥2 unless otherwise stated. Cysteine identifiers per compound needed to have H/L ratios in both replicates. Data was further filtered so that ligandability criteria required both replicates to have a Log2(H/L) ratio of ≥2 if the SD exceeded 2. The total ligandable cysteines stated excluded cysteine residues liganded by SO56 and SO59 due to their high proportion of “ligandable” cysteines (*i.e.*, apparent high reactivity). For Figure 5 experiments, duplicates were deemed sufficient for benchmarking the low-cost magnetic beads to the standard SP3 magnetic beads (control) in terms of coverage.

## Results

### Increased Tryptic Digest Concentration Fails to Enable Lower Volume Sample Preparation

The first step to transition our cysteine chemoproteomic workflow into a more high throughput platform was to scale down the volumes of key steps of our established method. In our previous SP3-enabled cysteine chemoproteomic sample preparation workflow (Fig. 1*A*), the maximum volume reached during SP3 cleanup is 4 ml, which far exceeds the maximum volume available for a 96-well plate (2 ml for a 96-well deep well plate)—this large volume is required during the acetonitrile (>95% v/v) peptide-binding step that occurs after the on-SP3 tryptic digest (Supplemental Table S1). Furthermore, the shaking steps required for peptide binding to beads also limits the volume allowed in a deep-well plate, with excess volume leading to inadvertent mixing between wells during shaking. Thus, to transition our method from 1.5 ml microcentrifuge tubes to a plate format, we first focused on the scaledown of this step. We first postulated that scaledown could easily be achieved by implementing an ultra-high concentration and low volume tryptic digest (15 μl). We tested the impact of this scaledown for cysteine chemoproteomic samples prepared using our v1.0 workflow (Fig. 1*A*) with varying trypsin digest volumes, for Jurkat whole cell lysate (200 μg total material) labeled with IAA followed by click conjugation to biotin azide (Supplemental Fig. S1). Disappointingly, but consistent with prior reports of high protein concentration negatively impacting proteomic coverage (75), we observed a decrease in peptide recovery with increased digest concentration (Fig. 1*B*). Coverage of enriched biotinylated cysteine peptide spectral matches and unique cysteines identified also decreased dramatically (∼50% loss) when the digest protein concentration exceeded 4 mg/ml (Fig. 1, *C* and *D*). Alongside this decrease in cysteine peptide coverage, we also observed an increased capture of background peptides lacking cysteine-biotin modifications for higher digest concentrations (Fig. 1*E*).

### Bypassing Peptide Cleanup Step Enables High Coverage and Low Volume Chemoproteomics

While our prior studies had indicated that SP3-cleanup is highly beneficial for increasing coverage by removing trace biotin contaminant (41, 42), we were inspired by prior studies that have shown that the SP3 peptide-level cleanup step can be skipped for various downstream applications, including phosphoproteomics (62) and TMT labeling (4, 63, 76, 77, 78). Therefore, we next opted to test whether the peptide cleanup step could be omitted while maintaining high coverage (Fig. 3*A*). Gratifyingly, no significant change in coverage was observed for samples prepared with the omission of the peptide cleanup step (Fig. 3*B*). We term this new cysteine chemoproteomic workflow, which omits the peptide cleanup step on SP3 prior to avidin enrichment, workflow v2.0, and the original workflow outlined in Yan *et al.* 2021, as workflow v1.0. Further illustrating comparable performance, a substantial overlap for cysteines identified was observed when both workflows were done side by side (Fig. 3*C*), and similar coverage when compared to CysDB annotations of identified cysteines (68) (Fig. 3*D*). Notably, peptide neutravidin eluants were directly analyzed with no further cleanup—to ensure that samples contained no substantial residual contaminants from the tryptic digest, we incorporated additional resin washes during the neutravidin capture step (see Experimental Procedures). We observed no significant difference in the proportion of enriched modified peptides between the two workflows (Supplemental Fig. S3). The elimination of a peptide-cleanup step on SP3 beads cuts the sample preparation time by at least 90 min and minimizes sample loss by reducing sample transfer steps. To ensure successful implementation of the v2.0 workflow, we recommend maintaining the digestion protein concentration within a range of 2.67 to 4 mg/ml, which corresponds to a volume ∼100 to 150 μl, and avoids excessively concentrated protein digests that can negatively impact digest efficiency, as reported previously (75).

### Streamlined SP3 Cleanup is Compatible with Small Molecule Screening

Pleased with the overall coverage of our newly modified and more streamlined SP3-chemoproteomic sample preparation method, we next turned to testing its utility for covalent ligand discovery. Following the workflow shown in Supplemental Fig. S4, we subjected Jurkat whole cell lysates to our benchmarking scout fragment KB2 (Supplemental Fig. S2), for which we (3, 40, 79) and others (4, 21, 22, 68) have generated many high coverage prior datasets. For quantitative chemoproteomic target engagement analysis, we utilized isotopically labeled (heavy and light) biotin azide enrichment tags that can be easily clicked to IAA (Supplemental Fig. S1), enriched on neutravidin resin, and eluted from the resin under mild acid conditions. These tags have been shown to provide reliable ratios when used for quantification (38). We tested both our biotin azide-based activity protein profiling (ABPP) under the workflow v1.0 cysteine chemoproteomic conditions (Fig. 1*A*) and our newly optimized workflow v2.0 (Fig. 3*A*) in parallel to further evaluate the scope of our newly modified cleanup strategy in the context of cysteine chemoproteomics (Supplemental Fig. S4). We observe similar coverage to our prior studies for both methods, with high overlap in coverage (74% of identified cysteines shared) (Supplemental Fig. S5) as well as similar ligandability profiles (Supplemental Fig. S6, *A* and *B*) and types of cysteines captured (as annotated by CysDB) (Supplemental Fig. S6, *C* and *D*). We observe generally strong concordance between liganded cysteines identified between both workflows (Fig. 3*E*) and those previously reported as engaged by KB2, both for our group's prior datasets and for those generated more broadly, as reported by CysDB (68) (Fig. 3*F* and Supplemental Fig. S6).

To assess whether our newly modified SP3 workflow is also compatible with other reagents besides our isotopically tagged biotin azide capture reagents, we compared them against isoTOP-ABPP TEV cleavable biotin reagents (5) and MS1 compatible sCIP reagents (40) using the same electrophile KB2. Both reagents require streptavidin enrichment followed by either TEV or acid-assisted cleavage from the resin. We observed similar cysteine coverage across all reagents (Supplemental Fig. S7), illustrating the generalizability of this workflow to various custom capture reagents and enrichment types.

### Transitioning Our Enhanced SP3 Method into a 96-Well Plate Format

The key overarching goal of our study was to achieve streamlined well-plate sample preparation, with a particular focus on compatibility with large sample inputs that are not amenable to the state-of-the-art SP3-TMT methods (21, 39). Therefore, our next step was to transition our method from 1.5 ml microcentrifuge tubes to a 96-well plate. To enable compatibility with >200 μg sample input, we selected a 2 ml deep well plate as the optimal container to match anticipated workflow volumes (Supplemental Table S2). We then purchased two models of plate (round U-bottom and conical V-bottom) and compared capture of SP3 resin by two widely used magnets (80, 81, 82), Magnetic Stand-96 *versus* DynaMag 96. We find that the conical bottom plates paired with the Magnetic Stand-96 afford improved ease of handling and similar coverage as compared to other options assessed (Supplemental Fig. S8). Visual inspection rationalized the increased performance of the Magnetic Stand-96, which features round magnets, compared to the DynaMag 96 side skirted plate magnet, with the former affording improved SP3 resin capture (Fig. 2, *A–C* and Supplemental Fig. S8). Lastly, our choice of plate was also guided by compatibility with reusable plate seals that are compatible with the vigorous shaking steps in the SP3 workflow.

Benchmarking against our microcentrifuge-based approach revealed comparable cysteine chemoproteomic coverage for our newly established well-plate–based platform (Supplemental Fig. S9, *A–C*). Further illustrating the robustness of our method, we find that similar coverage was observed for the enrichment on magnetic neutravidin, with magnet-based resin cleanup, compared to conventional neutravidin, with centrifugation-based cleanup (Supplemental Fig. S9, *A–C*). Notably, this advance allows for full plate-based sample preparation, which substantially minimizes both manual sample manipulation and preparation time.

Showcasing the versatility of our approach, we observe high coverage of cysteine peptides across a range of protein inputs, spanning 100 to 800 μg, with a slight decrease in coverage at lower 50 μg input amounts (Supplemental Fig. S10*A*). While this plate-based platform is compatible with larger volumes, for cysteine chemoproteomics, 200 μg proved sufficient to achieve high coverage (Supplemental Fig. S10, *A* and *B*). We note that the compatibility of our method with larger volumes could be useful for studies using less reactive probes (66). These findings align with our prior report for down-scaling of microcentrifuge-based sample preparation (41), and we expect that, when paired with isobaric multiplexing, further scale down should be feasible, consistent with the recent report by Gygi *et al.* (39). Additionally, when compared to more dilute chemoproteomic samples, we observed that higher concentration and lower volume samples afforded a modest increase in coverage (Supplemental Fig. S10*B*), which further highlights the value of our reduced volume approach.

### CySP3-96 Enables Rapid Screening of Focused Sets of Cysteine-Reactive Electrophilic Compounds

**Fig. 6:**
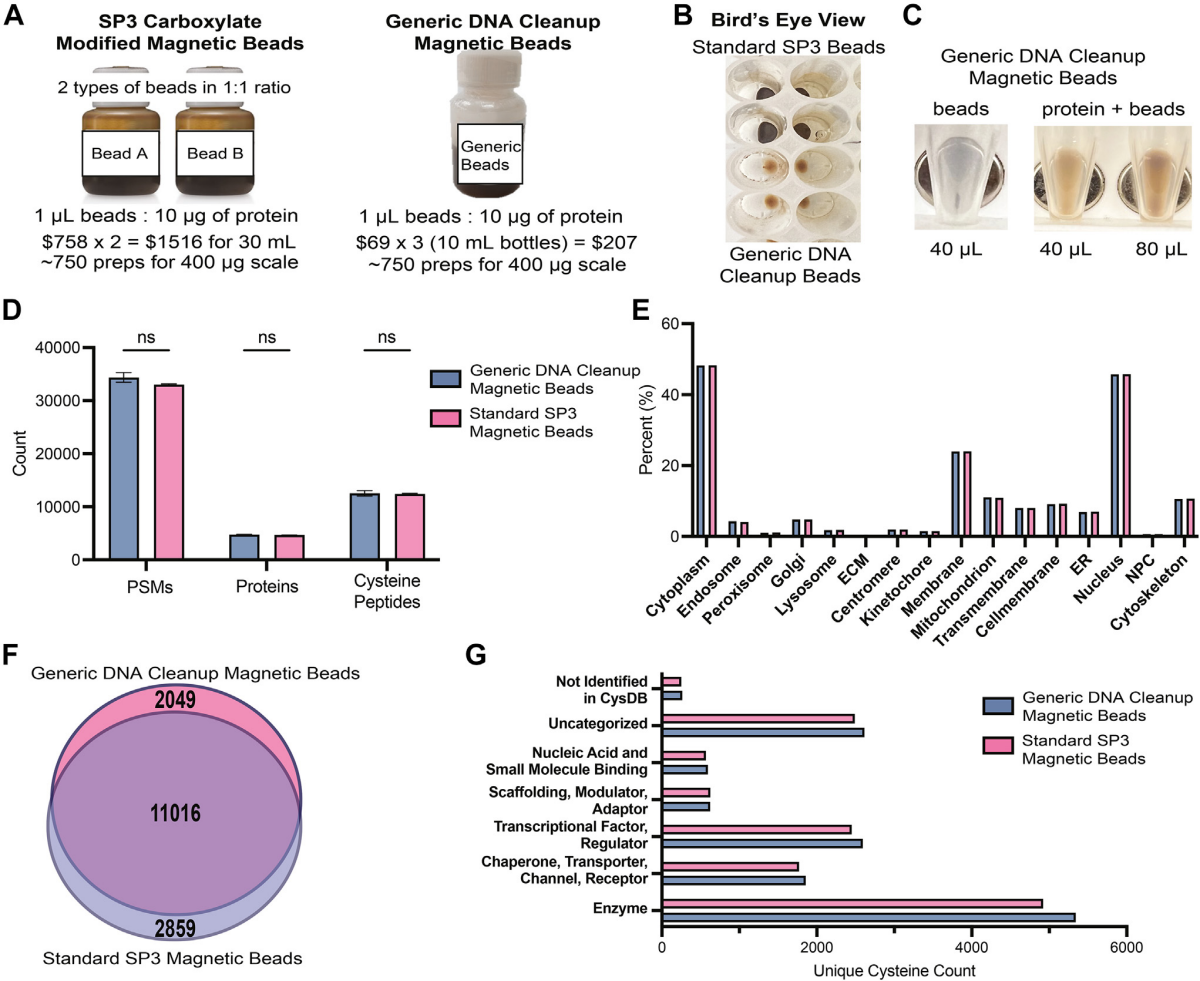
**Identifying a low-cost magnetic option for SP3.***A*, cost analysis of using SP3 resin (standard SP3 beads) *versus* commercially available magnetic beads (generic DNA cleanup beads) usually used for oligonucleotide purification. *B*, shows deep-well plate setup with generic DNA cleanup magnetic beads (bottom 2 rows) or standard SP3 beads (top two rows) to show differences in bead volume. Smaller bead volumes allow for more free space for tips when aspirating washes. *C*, shows generic DNA cleanup beads without protein and when protein is immobilized onto beads. *D*, comparison of PSM, protein, and cysteine coverage between the same volume of generic magnetic beads as compared to standard SP3 beads, n = 2. *E*, classes of proteins detected, based upon UniProtKB annotations, from sample preparations using the magnetic beads or SP3 beads. *F*, overlap of unique cysteines identified by each bead type. *G*, categorization of each cysteine identifier from CysDB for each bead type. Statistical significance was calculated by performing an unpaired Student’s *t* test. *p* < 0.05 for significance cutoff. All MS data is available in Supplemental Table S7.

With our plate-based method established, we next opted to deploy our approach to screen a focused library of cysteine reactive electrophiles in Jurkat whole cell lysates. We assembled a panel of two previously screened electrophilic compounds, the aforementioned KB2 together with KB7 (3, 40, 79), and 12 newly synthesized fragment-like molecules that span a range of electrophilic chemotypes (Supplemental Fig. S1). We additionally included two more elaborated atropisomeric compounds into our screening library (BEH-1-(*S*_a_) and BEH-1-(*R*_a_)), which were designed based on the covalent receptor tyrosine-protein kinase erbB-2 (HER2) and epidermal growth factor receptor kinase inhibitor neratinib that is used to treat HER2+ breast cancer (91) and synthesized in 25% yield (Fig. 6).

Then, following the scheme shown in Figure 2*D* (detailed workflow shown in Supplemental Fig. S11), we subjected Jurkat whole cell lysates to each compound or DMSO vehicle. Following compound incubation and click conjugation to isotopically enriched biotin-azide capture reagents, the samples were combined pairwise across the plate to generate 48 unique samples for competitive chemoproteomic analysis (Supplemental Fig. S4 workflow). To further showcase the versatility of our workflow and to better pinpoint high affinity targets, we also generated dose-response datasets, screening BEH-1-(*S*_a_) and BEH-1-(*R*_a_) at a range of compound concentrations spanning 5 μM to 100 μM. In total, across all datasets, 12,265 unique cysteines from 4375 proteins were quantified. This coverage is comparable to that reported for other recent high coverage chemoproteomic screening platforms (21, 83). Coverage was highly consistent across all wells and compounds analyzed (Fig. 2, *E* and *F* and Supplemental Fig. S12), illustrating that the CySP3-96 workflow does not introduce plate position-based biases or other significant sources of variability.

Prior studies have revealed that apparent cysteine-reactivity can vary considerably across different electrophiles and compounds of varying electronics and lipophilicities (1, 3). Therefore, we next assessed the relative compound reactivity, as inferred from the fraction of cysteines liganded (Log_2_ (H/L) ≥ 2). We find that the topmost reactive compounds are SO56 and SO59, which have the same scaffold and adduct 23% and 28% of the identified cysteines (Fig. 2*F*). By contrast, most other compounds (other than KB2 and KB7) showed attenuated reactivity <12%, including the two atropisomeric compounds. To further corroborate these findings, we performed gel-based analysis and assessed the relative competition of IARho labeling by each compound, which revealed labeling patterns generally consistent with our calculated proteomic reactivity (Supplemental Fig. S13). Thus, CySP3-96 can capture the relative reactivity of different cysteine reactive electrophiles across the proteome.

To further vet our CySP3-96 screening data, we compared the liganded cysteines (Log2(H/L) ≥ 2) identified for widely used compounds, KB2 and KB7, to those identified previously. We observe a number of shared targets, including peroxiredoxin-5 mitochondrial (PRDX5) Cys 204 and cyclin-G-associated kinase (GAK) Cys 190 for KB2 as well as mitogen-activated protein kinase 9 (MAPK9) Cys177 for KB7 and KB2 (3, 4) (Supplemental Fig. S14). Looking more broadly across our data, we identified 2280 total ligandable cysteines across all compounds screened. A number of highly ligandable cysteines stand out, including Cys1101 in reticulon-4 (RTN4) and Cys114 in fructose-2,6-bisphosphate TIGAR (TIGAR) (Supplemental Fig. S14), which are also identified as very ligandable by prior studies (3, 68). These examples illustrate the capacity of CySP3-96 to identify bona fide cysteine labeling sites. Additionally, we found generally good concordance for the ratios measured for KB2 samples prepared in microfuge tubes using workflow v1.0 to the KB2 samples generated in the 96-well plate (Supplemental Fig. S15).

Looking beyond these well-characterized cysteines, we were also pleased to observe that our dataset captured 607 cysteines not previously identified in CysDB, including Cys 120 in G protein-coupled receptor kinase 3 (GRK3), Cys 835 in nuclear pore complex protein Nup98-Nup96 (NUP98), and Cys 403 in ubiquitin carboxyl-terminal hydrolase 48 (USP48) (Fig. 2*G*). Consistent with bona fide liganding events, we note that many of these newly observed cysteines observed SAR across the compound library. For example, while Cys 2 in transport and golgi organization protein 2 homolog (TANGO2) was generally insensitive to most compounds tested, this cysteine was sensitive to both of the highly structurally related compounds SO60 and SO68 (Fig. 2*G*). Similarly, for SO60 and SO68, both were observed to engage Cys 319 in protein CBFA2T3 (CBFA2T3) and Cys 192 in zinc finger C4H2 domain-containing protein (ZC4H2). Furthermore, the structurally similar compounds SO237 and SO238, which showed very attenuated proteome-wide reactivity, both liganded Cys 36 in serine/threonine-protein kinase MRCK beta (CDC42BPB) (Fig. 2*G*). Analysis of the cysteines not previously reported in CysDB revealed that 24% of the new liganded cysteines (engaged by 2 or more compounds) belong to the transcription factor and/or regulator tough-to-target class of proteins (Supplemental Fig. S16). The next most liganded groups were the proteins classified under the enzyme group, the scaffolding, modulator, adaptor group, and the uncategorized group, each containing 17% of the cysteines in the group to be liganded by two or more of the compounds used for screening (Supplemental Fig. S16) We ascribe newfound ligandability both to the novel compounds in our screening library together with the robust coverage afforded by our CySP3-96 platform. To further understand how our dataset compares to prior studies, we compared the relative ligandability (fraction of compounds screened that label a cysteine) for CysDB data compared to our CySP3-96 screen. Gratifyingly, the relative ligandability trends corroborate similar performance of our platform to prior studies, with greater ligandability in CysDB matching that observed in our CySP3-96 screen (Fig. 2*H*).

In addition to these newly ligandable cysteines, we also note the presence of cysteines that show compound-induced increased labeling by IAA, as indicated by negative log2(H/L) ratios. While we cannot exclude the possibility that some of these IDs could stem from some degree of stochasticity, we rationalize these negative values as likely stemming from so-called “anti-ligandable” cysteines, which are cysteines that show an increase in IAA labeling concurrent with a parallel liganding event at another cysteine in the same protein, as reported recently by Takahashi *et al.* (4). Consistent with this likelihood, we note a number of anti-ligandable cysteines are shared between our KB2 dataset and the prior report, including Cys 249 in small ribosomal subunit protein RACK1 (RACK1), Cys 40 in UMP-CMP kinase 2 mitochondrial (CMPK2), Cys 111 in ubiquitin-conjugating enzyme E2 D2 (UBE2D2), and Cys 98 in 14-3-3 protein epsilon (YWHAE).

Taken together, these examples illustrate the capacity of CySP3-96 to identify established and novel ligandable cysteines and to enable high coverage screening of focused electrophilic libraries.

### Atropisomeric Electrophiles Show Distinct Proteome-wide SAR

As atropisomers feature conformationally stable enantiomers (84), we hypothesized that BEH-1-(*S*_a_) and BEH-1-(*R*_a_) compounds would each engage unique cysteines. To test this hypothesis, we first stratified the cysteine ligandability ratios across the concentration range screened for each compound and compared the ratios obtained for the BEH-1-(*S*_a_) and BEH-1-(*R*_a_) compounds (Fig. 4*A*). Consistent with our hypothesis, we find that 66 total cysteines are preferentially labeled by one atropisomer. Of these, 30 cysteines had not been found to be ligandable in CysDB, which are highlighted with bolded identifiers in Figure 4*B*. As the general reactivity of each compound was observed to be comparable (Fig. 2*F*), we expected that these differences were reflective of bona fide SAR.

These newly liganded cysteine proteins include DNA/RNA-binding proteins, such as Cys208 in non-POU domain-containing octamer-binding protein and Cys169 in eukaryotic translation initiation factor 3 subunit. We were also pleased to observe that both atropisomers engaged many kinase cysteines, including residues such as Cys317 in uridine-cytidine kinase-like 1 (S_a_), Cys9 in serine/threonine-protein kinase 38 (S_a_), and Cys340 in beta-adrenergic receptor kinase 1 (R_a_). This observation aligns with the nature of these compounds as analogs of known kinase inhibitors. Further highlighting enantioselective SAR reported by our platform, we find that BEH-1-(*S*_a_) shows increased labeling of transferase/kinase annotated proteins as compared to BEH-1-(*R*_a_) (Supplemental Fig. S17). Illustrating this activity, we see that the active-site adjacent calcium/calmodulin-dependent protein kinase type IV Cys202 was preferentially engaged by BEH-1-(*S*_a_) (Fig. 4*B*). Highlighting possibilities for distinct binding modes, we observed that Cys231 in CHEK2 was preferentially engaged by BEH-1-(*R*_a_), particularly at lower compound concentrations (Fig. 4*B*). These compelling examples illustrate the utility of screening atropisomeric compounds for target hunting. We do also note that we did not identify cysteines from the neratinib targets epidermal growth factor receptor and HER2, likely due to very low levels of protein expression in Jurkat cells and the lack of inhibition of these kinases observed for BEH-1-(*S*_a_) and BEH-1-(*R*_a_), in an established kinase activity assay (Supplemental Fig. S18).

Intrigued by the preferential labeling of CHEK2 by the *R* atropisomer, we next subjected CHEK2 (PDB ID: 2XBJ) to covalent molecular docking using MOE software, with the goal of assessing whether the chemoproteomic data could be corroborated in silico. Consistent with the chemoproteomic-preference for BEH-1-(*R*_a_), we find that most (4/5) of the binding poses observed for BEH-1 adopt the R conformation (Fig. 4*C* and Supplemental Fig. S19). The best R enantiomer-binding pose was also observed to exhibit the lowest overall binding energy, with an MOE predicted affinity score of −5.0164 (Fig. 4*C*). In addition to the covalent linkage, multiple contacts were observed between CHEK2 and the BEH-1-(*R*_a_) atropisomer within this pocket (Fig. 4, *D*, *E* and *F*). Taken together, these docking studies corroborate the chemoproteomic reported enantioselective labeling at CHEK2.

### Establishing a Low-Cost Option for Magnetic Bead-Based Sample Cleanup

Achieving cost savings was a key goal of our study. Therefore, we next sought to identify budget friendly SP3 resin. We were inspired by recent studies that had shown, for protein cleanup, low cost glass beads function comparably to higher cost magnetic resin (85), albeit using centrifugation to isolate the resin. Other studies have also shown that the surface chemistry of beads is largely irrelevant to sample preparation, which is facilitated by protein precipitation rather than true binding to beads (86). Thus, we hypothesized that other low-cost magnetic resins would function comparable to the widely used SP3 resin (standard SP3 beads). To test this hypothesis, we selected a low-cost, commercially available resin (generic DNA cleanup beads) as a reagent for oligonucleotide cleanup (Fig. 5*A*). Visual inspection of the appearance of each resin slurry revealed that the low cost resin appeared more dilute, which was confirmed by the lower compacted bead volume (approximately 1/5th the size for the same volume taken, upon visual inspection) (Fig. 5, *B* and *C*). Analysis of each bead type revealed that the standard SP3 beads had a concentration of ∼5 × 10^8^ beads per mL of resin slurry while the generic DNA cleanup beads had a concentration of ∼1 × 10^8^ beads per mL of resin slurry, corresponding to approximately 4.4× difference in concentration. Thus, we opted to adjust the volume of slurry used to match the compacted bead volume between the two resins for our initial proteomic sample preparation. Gratifyingly, head-to-head chemoproteomic coverage comparison revealed no significant difference in coverage between the two resins (Fig. 5*D*).

As the scaled-up slurry volume would diminish the potential cost-savings, we also tested the performance of lower slurry volumes (Supplemental Fig. S20). We find the coverage is unchanged when the magnetic beads were used in the same ratio as the SP3 beads: 1 μg of protein to 10 μl of slurry (Fig. 5*D* and Supplemental Fig. S20). Additional inspection of the size of the beads revealed that the standard SP3 beads (∼4.23 μm) are slightly larger than the generic DNA cleanup beads (∼3.11 μm) (Supplemental Fig. S21). We hypothesize that the slightly smaller size of the generic DNA cleanup beads allows for more surface area for proteins to bind and/or aggregate onto, enabling the use of smaller bead volume. Visual inspection of these samples revealed a striking advantage of the low-cost resin: the smaller bead volume of the magnetic beads allows for more space within the well for aspirating supernatant throughout this workflow (Fig. 5*B*). The low bead volume also afforded a visual cue of efficient protein capture, namely an increase in the whitish hue of resin that occurs after protein precipitation (Fig. 5*C*). As a note of caution, we do acknowledge that ultra-low resin volumes (<40 μl bead slurry), particularly for small volume samples, may result in protein pellets that are challenging to visualize. Therefore, we do not recommend using bead slurry volumes of less than 40 μl.

To further vet this resin, we also compared coverage of cysteines identified. We find that >80% of cysteines are captured by both resins with no significant difference in the classes of proteins observed for each resin (Fig. 5, *E* and *F*). We ascribe the handful of cysteines preferentially identified by each resin to the stochastic nature of data-dependent acquisition, although we cannot rule out differences in resin affinities. Lastly, we also assessed whether this new resin could also prove useful for peptide-level sample cleanup post-tryptic digest—while not required for the CySP3-96 method, peptide cleanup is highly useful for other methods, such as our redox proteomic platform SP3-Rox (43). We find comparable peptide recovery is achieved for both resins analyzed (Supplemental Fig. S22). In sum, these data provide compelling evidence of the general utility of the generic DNA cleanup resin, which affords savings of >85% across 750 sample preparations equates to over $1300 (Fig. 5*A*).

## Discussion

In this study, we developed a 96-well SP3 workflow, CySP3-96, for the high throughput processing of chemoproteomic samples. Taken together, CySP3-96 chemoproteomics performed comparably to other cutting-edge platforms, identifying in aggregate 12,265 unique cysteines, of which 607 had not been previously captured by CysDB.

To establish CySP3-96, we first address the scalability limitation of SP3, to enable sample preparations with large amounts of input material. We expect that this methodological innovation should prove particularly useful for applications where coverage is tightly linked to sample amount, such as chemoproteomics using less reactive probes. Likely the scalability of our approach will also prove beneficial in addition to workflows that pool samples at the protein level, thereby increasing sample amount per experiment. Such applications include for cysteine redox proteomics (SP3-Rox) (43) and for isobaric labeling studies using our sCIP reagents (40).

After extensive benchmarking to ensure the robustness of our plate-based workflow, we deployed our platform to screen a focused library of cysteine-reactive electrophilic compounds. Our screening library contains both previously reported and novel electrophilic compounds, including an elaborated pair of atropisomeric analogs of neratinib. In aggregate, we identify 2280 ligandable cysteines, including 67 residues with atropisomeric selectivity. As demonstrated by recent work using stereoprobe libraries (45), we expect that atropisomeric compounds should prove similarly useful in guiding the discovery of high confidence and actionable cysteine-compound pairs.

The increased sample throughput offered by CySP3-96 (between 1.5 h to 12 h decreased active sample manipulation time, Supplemental Fig. S23) facilitates testing dose-response across a range of compound concentrations. As shown for the pair of atropisomeric compounds tested here, testing a range of compound concentrations is valuable for the delineation of high *versus* low affinity labeling events. We expect that dose ranges should also prove useful for delineating on-target labeling from compound-induced changes to protein abundance caused by generalized cell stress—of note, our recent work revealed that many electrophilic compounds activate cellular stress responses, including stress granules and aggresomes when compound concentrations exceed low micromolar concentrations (87).

The many available, reliable, high performance, and high-throughput SP3 platforms, including for cysteine chemoproteomics (21, 39), highlight the burgeoning enthusiasm for SP3-based screening methods. Our CySP3-96 method is not intended to supplant these other impactful methods but rather to provide several innovations that are highly complementary to these existing platforms. Thus, we encourage broad consideration of the relative strengths of different approaches for those interested in establishing such SP3-based screening methods. We see our platform working well for those looking to reduce cost and for applications in which increased protein input is beneficial, for example, applications using less reactive probes in which scaled up protein input along with incorporation of offline fractionation will likely boost coverage. We envision a key area where the CySP3-96 method could prove impactful is for streamlining sample preparation *via* pooling of samples at the protein level, as has been recently used with custom, enrichable isobaric tags (40, 88) which are methods that necessitate larger-scale SP3 cleanup. Our method also incorporates plate-based avidin enrichment, which offers the opportunity for a fully automatable cleanup and enrichment in the future. We do also acknowledge the cumbersome pairwise sample pooling is a clear drawback of the current implementation of CySP3-96, which we hope to address *via* either the utilization of the aforementioned isobaric protein capture reagents or through implementation of label-free quantification strategies, most notably data independent acquisition.

Looking beyond chemoproteomics, we expect that our low-cost magnetic resin–based scalable high throughput cleanup workflow should prove useful for a wide range of applications that require increased sample input as well as increased number of samples. Our envisioned applications include for SP3-based phosphoproteomics workflows (62), SP3-based immunopeptidomics (89), and for upscaling for microflow-based sample separation (55, 67). Lastly, building on prior reports that revealed the surface chemistry flexibility of bead-based peptide cleanup, we anticipate that a range of magnetic resins will likely function comparable for both protein- and peptide-based sample decontamination.

## Data Availability

The MS data have been deposited to the ProteomeXchange Consortium *via* the PRIDE partner repository (90) with the dataset identiﬁer PXD054043. File details can be found in Supplemental Table S8.

## Code Availability

The scripts used for this work are available at https://github.com/BackusLab.

## Supplemental data

This article contains supplemental data (5, 40, 41, 68, 91, 92).

## Conflict of interest

K. M. B. is a member of the advisory board at Matchpoint Therapeutics. All other authors declare no competing interests.
