# Supplementary material for: CySP3-96 Enables Scalable, Streamlined, and Low-Cost Sample Preparation for Cysteine Chemoproteomic Applications
Source: Mol Cell Proteomics. 2024 Dec 18;24(9):100898. doi: 10.1016/j.mcpro.2024.100898 (PMC12529508; doi:10.1016/j.mcpro.2024.100898)
Supplement: Supporting Information [file mmc1.docx]

# Supplementary Figures


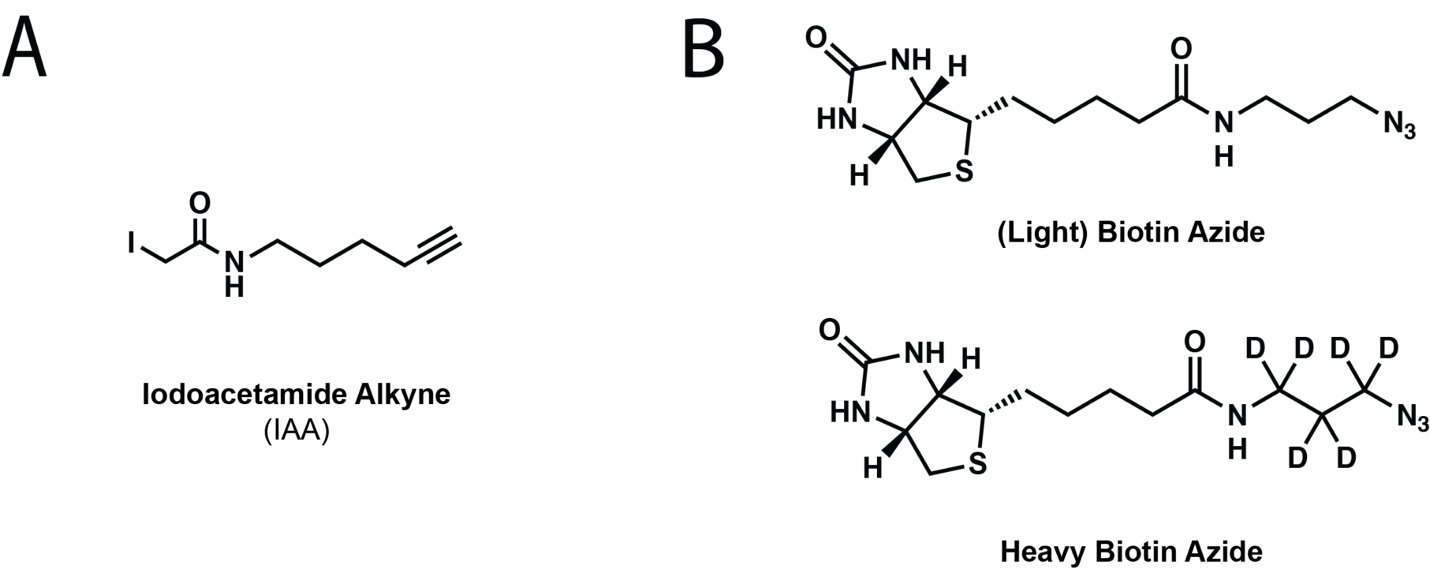


**Figure S1. Structures of capture reagents used in this study.** A) Structure of pan-reactive iodoacetamide alkyne (IAA)[^1^](https://sciwheel.com/work/citation?ids=1241563&pre=&suf=&sa=0). B) Structures of biotin azide enrichment handles[^2,3^](https://sciwheel.com/work/citation?ids=14615806,11898890&pre=&pre=&suf=&suf=&sa=0,0&dbf=0&dbf=0). Biotin azide and light biotin azide refers to top structure and is used in all experiments. Heavy biotin azide is used for ABPP experiments.


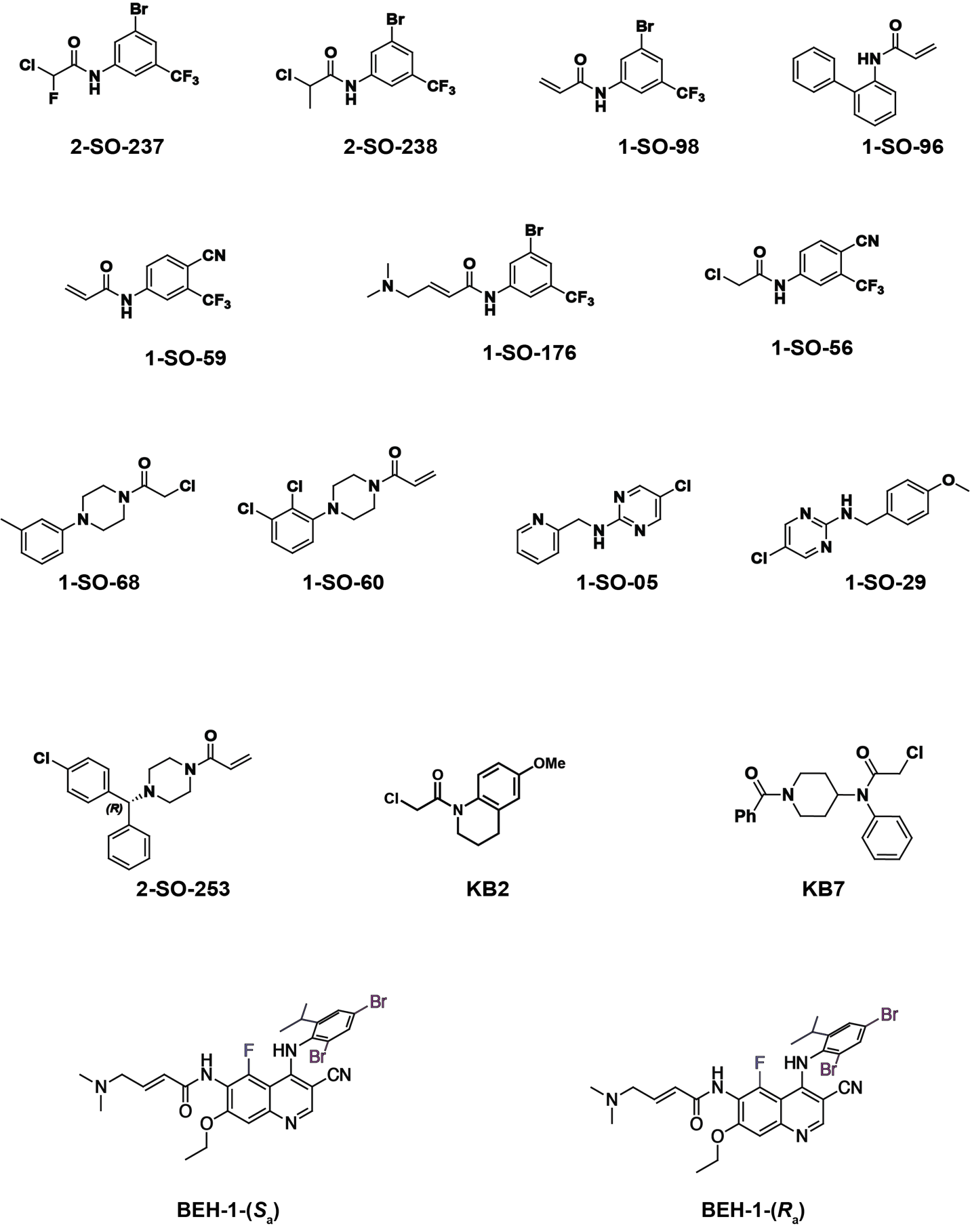


**Figure S2. Structures of electrophilic compounds used in this study.**


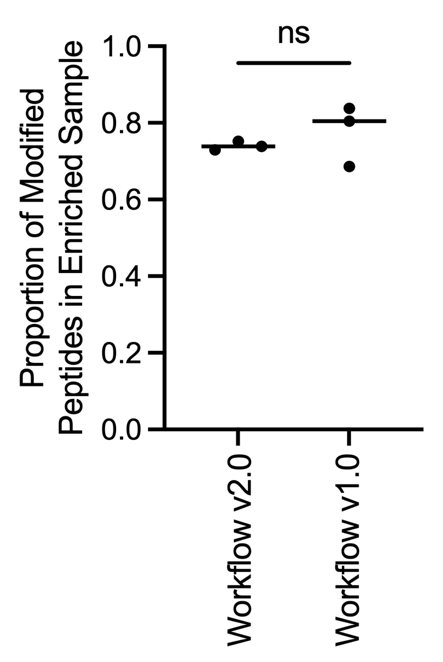


**Figure S3. Proportion of enriched modified peptides.** Analysis for proportion of modified peptides captured by enrichment for each workflow. Statistical significance was calculated by performing an unpaired Student’s t-test. p < 0.05 for significance cutoff, n=3. All MS data is available in **Table S4.**


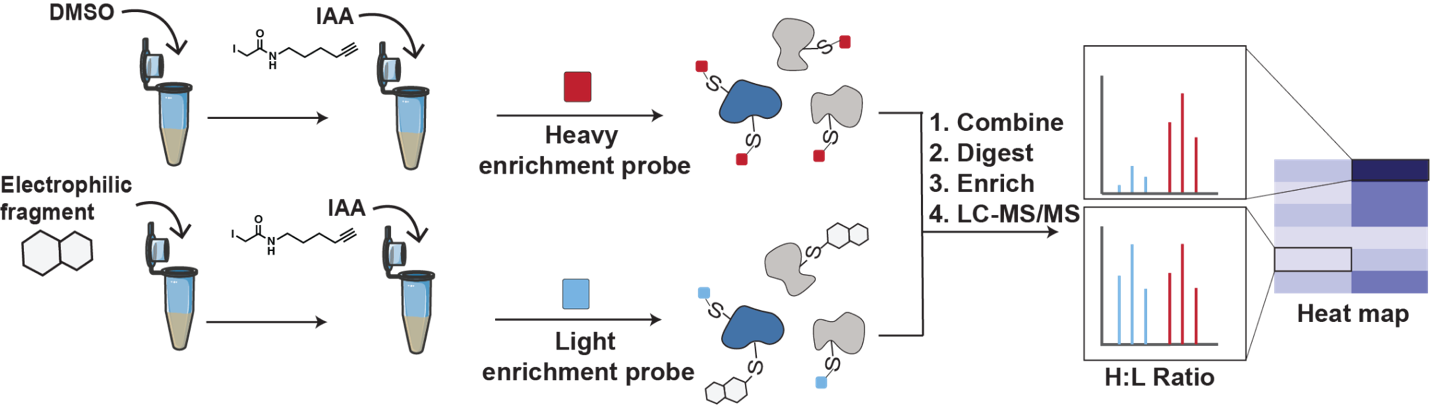


**Figure S4. Cysteine chemoproteomic electrophilic compound screening workflow.** Jurkat cell lysates are treated with either vehicle (DMSO) or electrophile-containing compound, for a short incubation period (1h) followed by labeling (1h) with the pan-cysteine reactive probe iodoacetamide alkyne (IAA). After alkyne functionalization, the samples are subjected to click conjugation with isotopically enriched biotin azide capture agents, pooled, digested with trypsin, enriched on neutravidin resin, and captured peptides analyzed by LC-MS/MS.


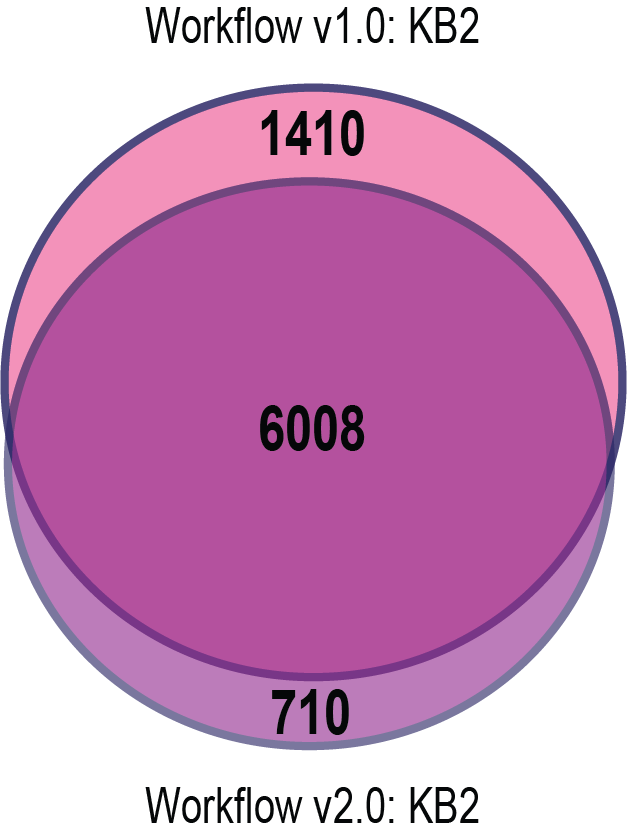


**Figure S5. Comparison of coverage obtained using v1.0 versus v2.0 workflows for cysteine chemoproteomics ligandability studies.** **A)** Analysis of unique cysteine identifier coverage between both workflows for KB2 (500 µM) competitive chemoproteomic analysis in whole Jurkat cell lysate (n=4). All MS data is available in **Table S5.**


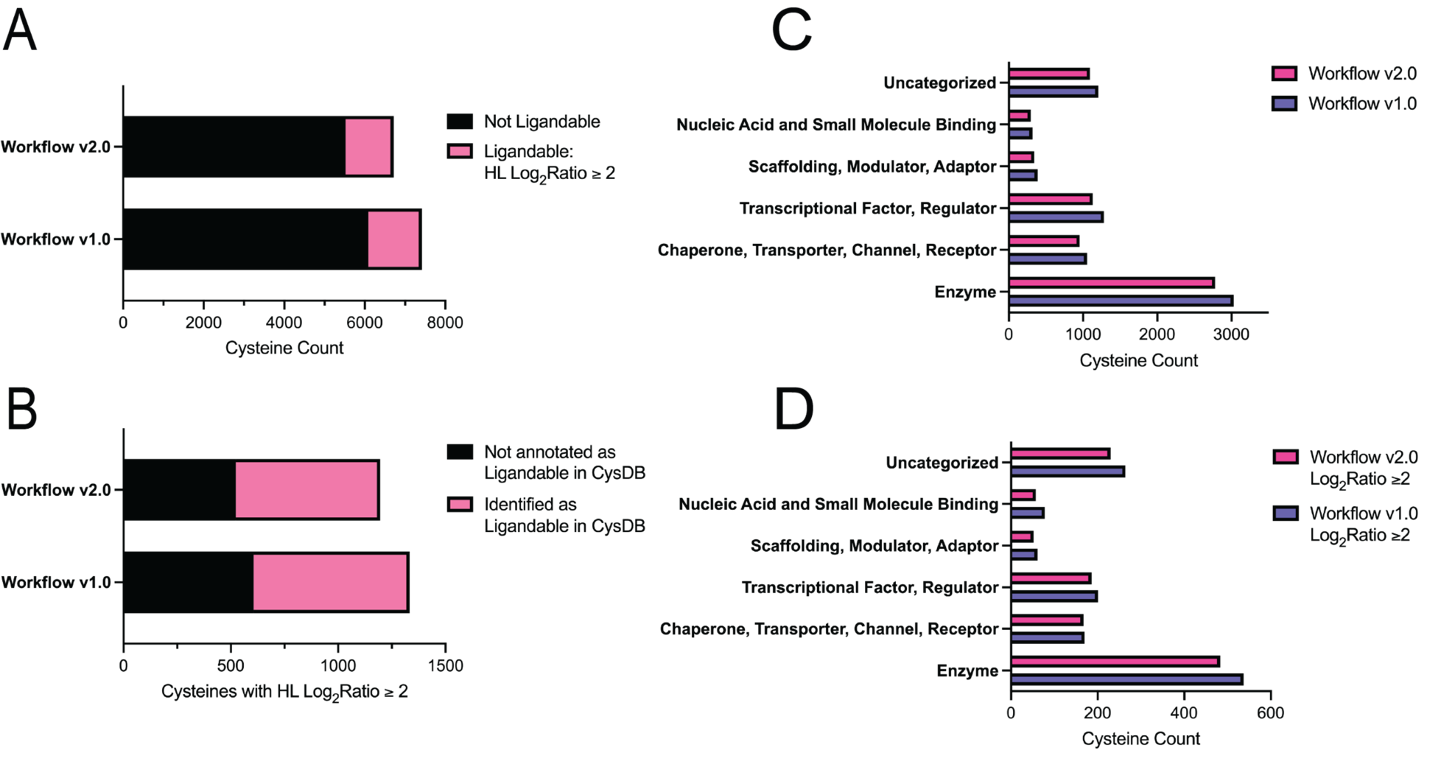


**Figure S6. Comparison of workflows for cysteine chemoproteomics ligandability studies with model compound KB2.** **A)** Analysis for proportion of unique cysteines showing a Log_2_(H/L) Ratio of 2 or greater for each workflow in relation to all unique cysteines identified in each experiment. **B)** Analysis for proportion of cysteines annotated in CysDB[^4^](https://sciwheel.com/work/citation?ids=14904677&pre=&suf=&sa=0) as ligandable for each workflow in relation to all ligandable cysteines (Log_2_(H/L) Ratio ≥ 2) identified in each experiment. **C)** Analysis for proportion of cysteine types as categorized by CysDB for both workflow conditions. **D)** Analysis for proportion of ligandable cysteine types as categorized by CysDB for both workflow conditions. Dataset used for these analyses are of KB2 (500 µM) treated competitive chemoproteomic analysis in whole Jurkat cell lysate, n=4 for each group. All MS data is available in **Table S5.**


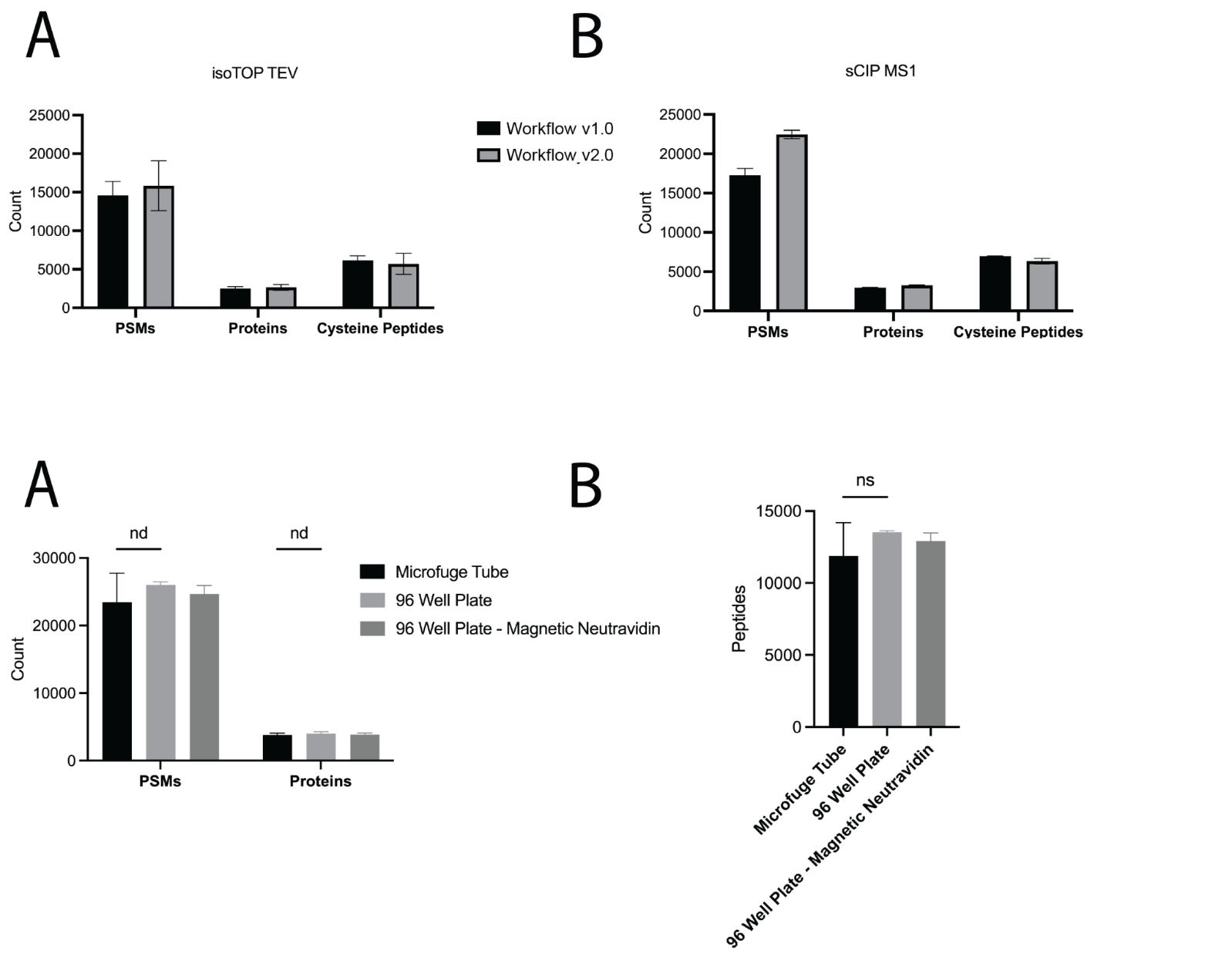


**Figure S7. Cysteine chemoproteomic workflow is compatible with multiple enrichment-handle reagents.** **A,B)** Comparison of coverage for samples prepared with either v1.0 versus v2.0 workflows for Jurkat cell lysates treated with KB2 (500 µM) or vehicle (DMSO), n=2, with A) samples prepared using previously reported SP3-cleanup[^5^](https://sciwheel.com/work/citation?ids=11898879&pre=&suf=&sa=0) enabled workflow for isoTOP-ABPP[^1^](https://sciwheel.com/work/citation?ids=1241563&pre=&suf=&sa=0) using TEV-protease cleavable biotin-azide (TEV tags), with enrichment on streptavidin resin and cleaved off by TEV protease. **B)** Samples prepared using silane-based cleavable isotopically labeled proteomics (sCIP) reagents[^6^](https://sciwheel.com/work/citation?ids=15549866&pre=&suf=&sa=0) with enrichment on streptavidin resin followed by low pH elution. All MS data is available in **Table S5.**


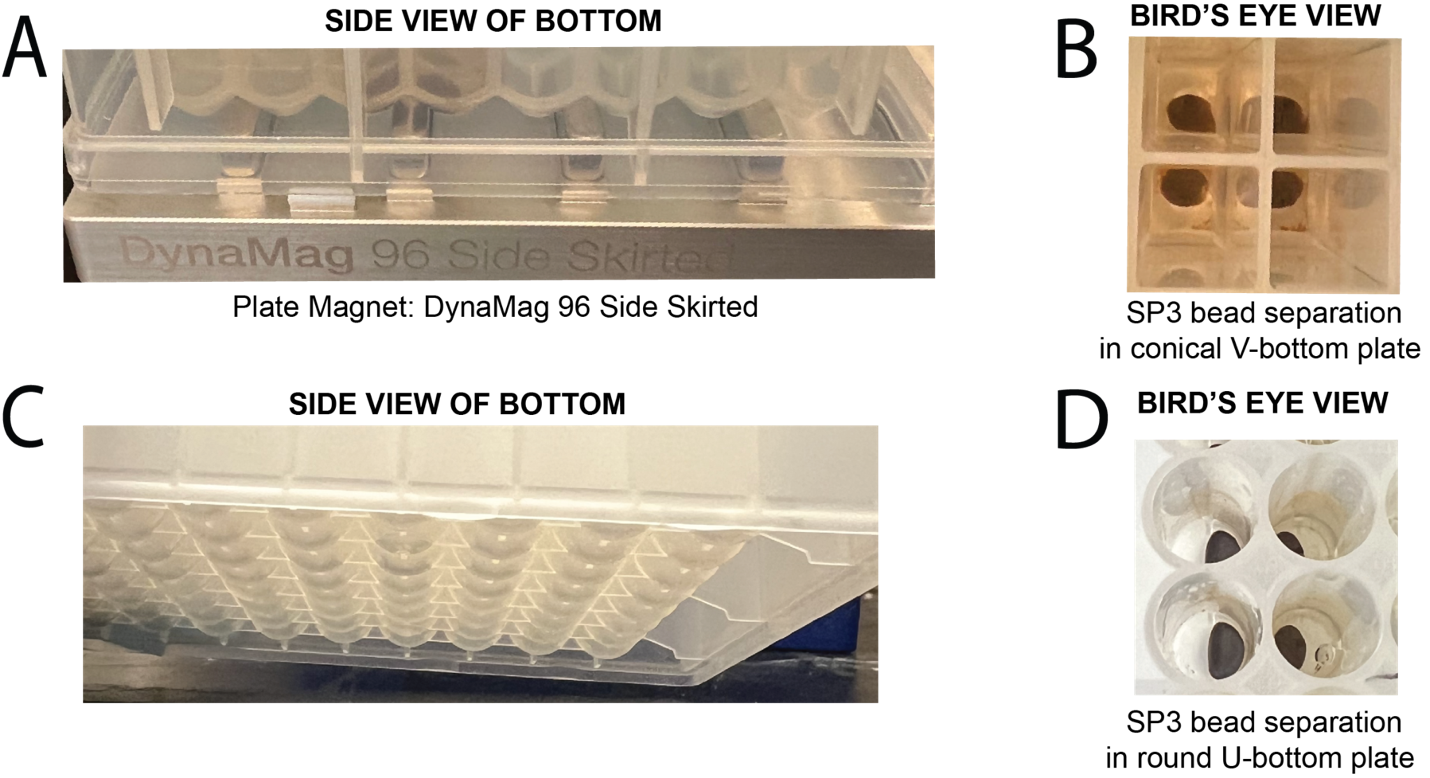


**Figure S8. Comparison of plates and magnets for CySP-96. A)** Conical V-bottom 96-well plate with a DynaMag 96 side skirted plate magnet (InVitrogen Cat. No. 12027). **B)** Birds eye view of the bead separation in a conical V-bottom plate using a Dynamag 96 side skirted plate magnet. **C)** Round U-bottom 96-well plate (MilliporeSigma™ Supelco™ SPE 96-Deep Square Well Collection Plate, Cat. No. 11-100-3690). **D)** Birds eye view of the bead separation in a round U-bottom plate using a Dynamag 96 side skirted plate magnet.


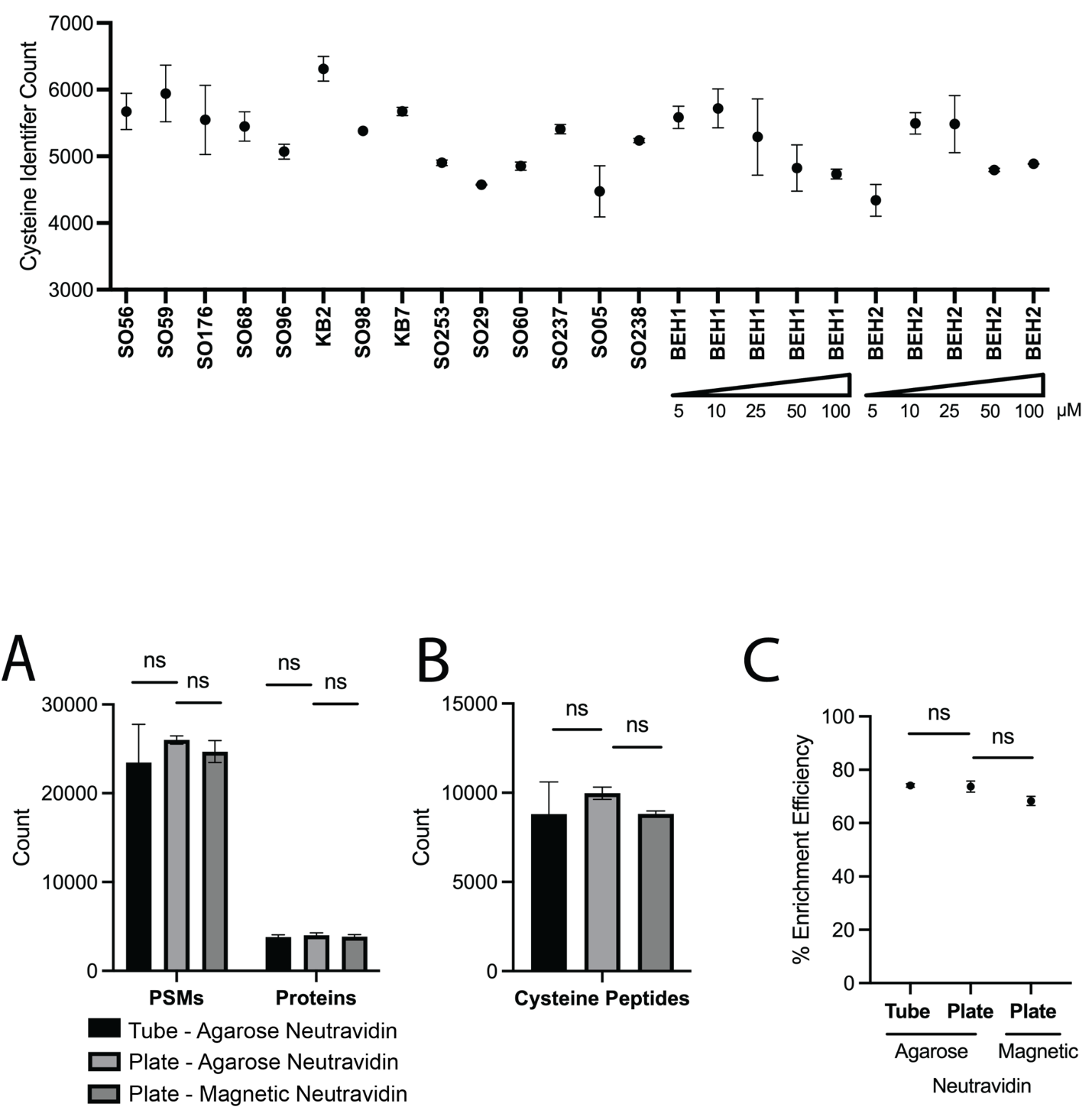


**Figure S9. Comparing coverage for samples prepared in microcentrifuge tubes versus 96-well plate format, including for neutravidin enrichment.** **A-C)** Comparison of coverage obtained using the indicated workflows, with “tube” corresponding to samples prepared with workflow v2.0 in 1.5 mL snap cap microcentrifuge tube and “plate” corresponding to samples prepared using v2.0 workflow in a 96-well plate. To test the impact of also performing neutravidin enrichment in the 96-well plate, samples were also prepared comparing enrichment plate-based sample preparation followed by neutravidin enrichment in snap cap microcentrifuge tube versus complete sample prep in the 96-well plate, including enrichment using magnetic neutravidin. **A)** Comparison of PSMs and proteins identified, **B)** Comparison of biotinylated cysteine peptides. **C)** Comparison of enrichment efficiency. Statistical significance was calculated by performing an unpaired Student’s t-test. p < 0.05 for significance cutoff, n=2. All MS data is available in **Table S6.**


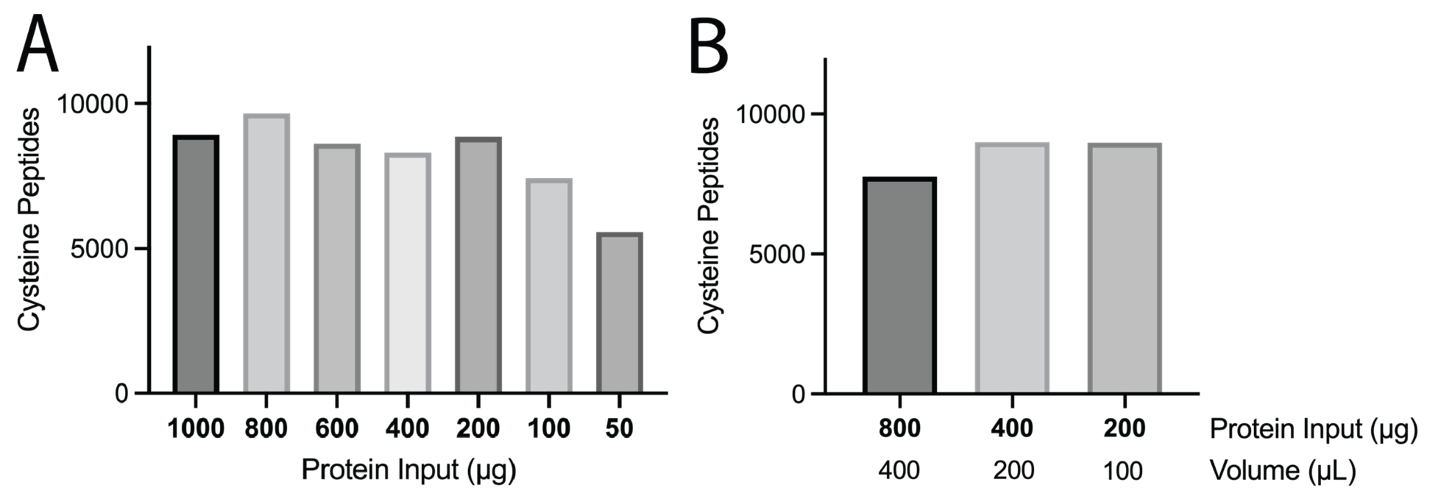


**Figure S10. Assessing the impact of protein input on cysteine coverage. A)** Coverage comparison of cysteine peptides enriched, for cysteine chemoproteomic samples prepared using the indicated protein input (Jurkat whole cell lysates, 50-1000 µg) at constant volume (200 µL) were labeled with IAA (200 µM) followed by click conjugation to biotin azide conjugation and chemoproteomic sample preparation analyzed for cysteine coverage, n=1. **B)** Coverage comparison of cysteine peptides for samples prepared using the indicated protein input (Jurkat whole cell lysates, 200-800 µg) while varying the input volume to maintain constant protein concentration (2 mg/mL). Input labeled with IAA (200 µM) followed by click conjugation to biotin azide conjugation and chemoproteomic sample preparation analyzed for cysteine coverage, n=1. All MS data is available in **Table S6.**


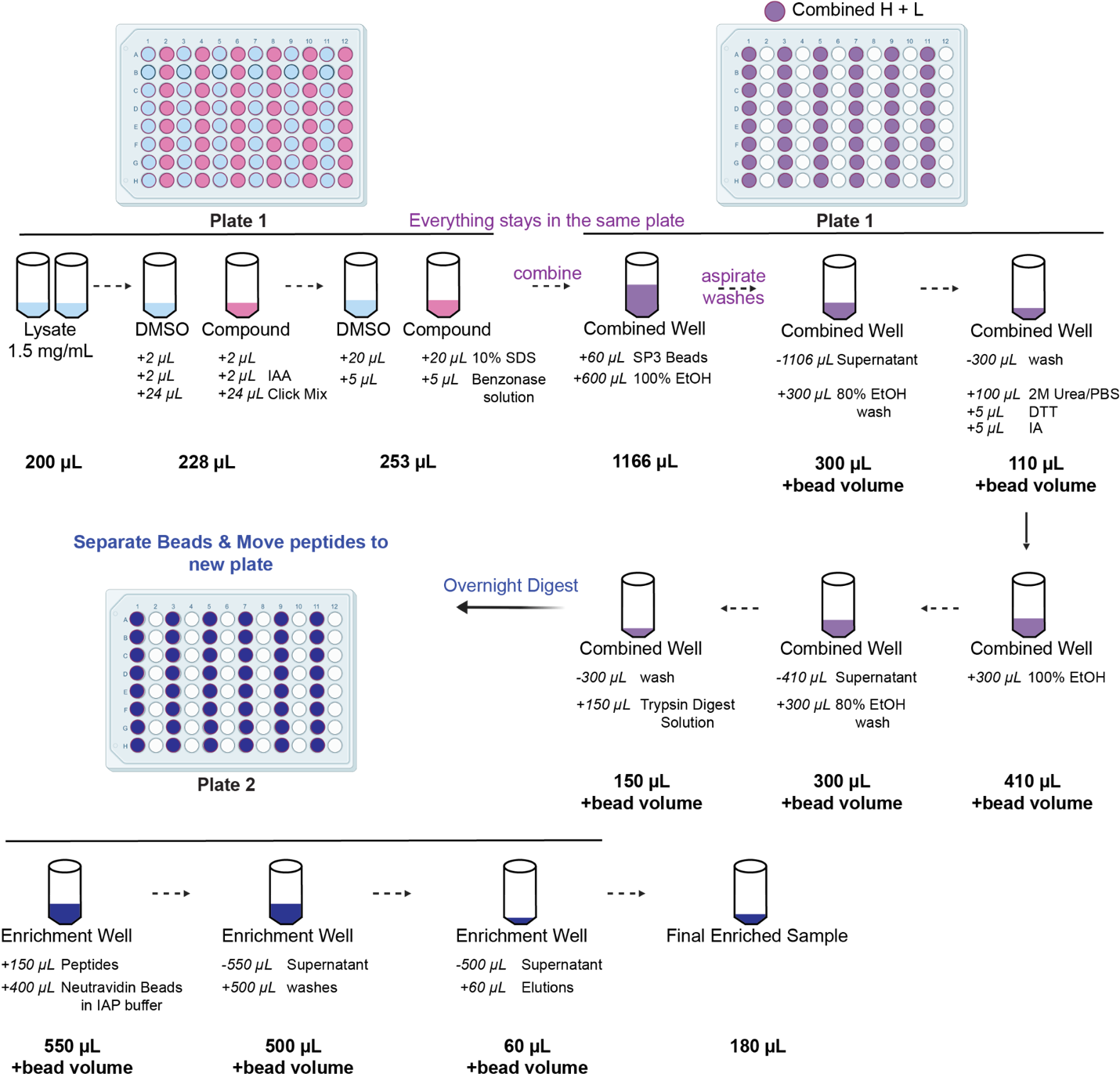


**Figure S11. Detailed workflow for CySP3-96 in plate.** Total volume in the well is shown for each step in bold and plate changes are noted where they occur. Note that for multiple washes & for multiple elutions only one is shown to represent the volume used per wash/elution.


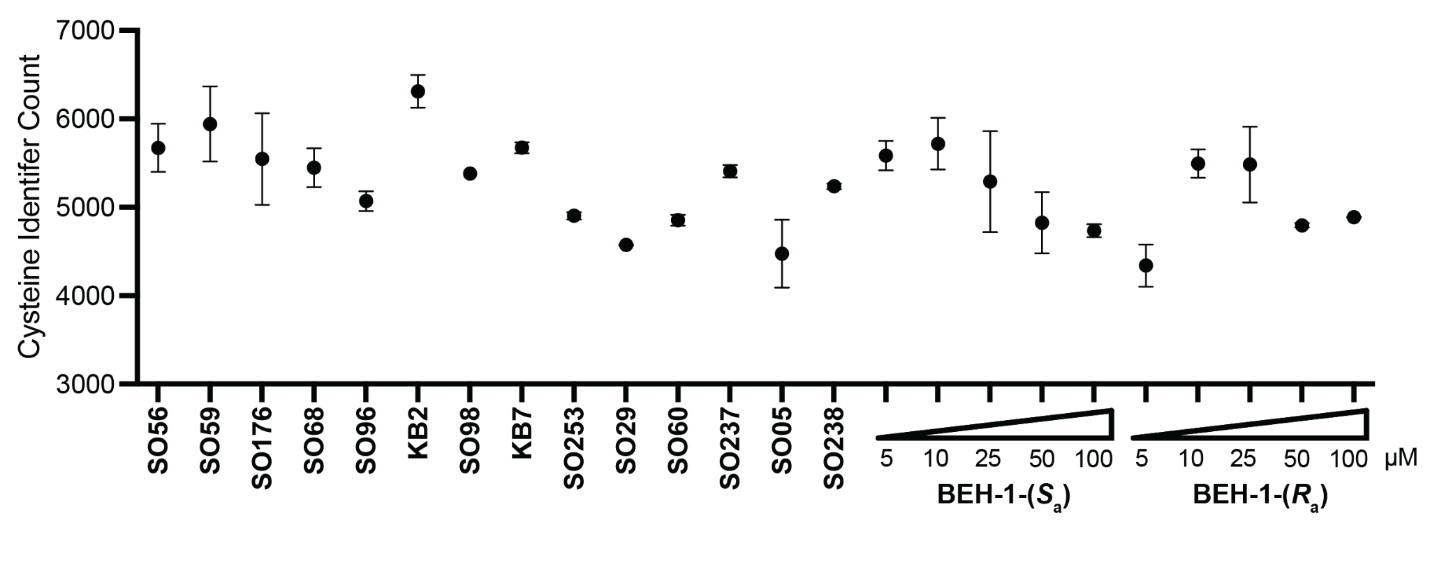


**Figure S12. Coverage and variability of unique cysteine identifiers for all compounds screened in plate.** All compounds screened at 500 µM (unless otherwise stated) in Jurkat whole cell lysates in replicates of 2. All MS data is available in **Table S6.**


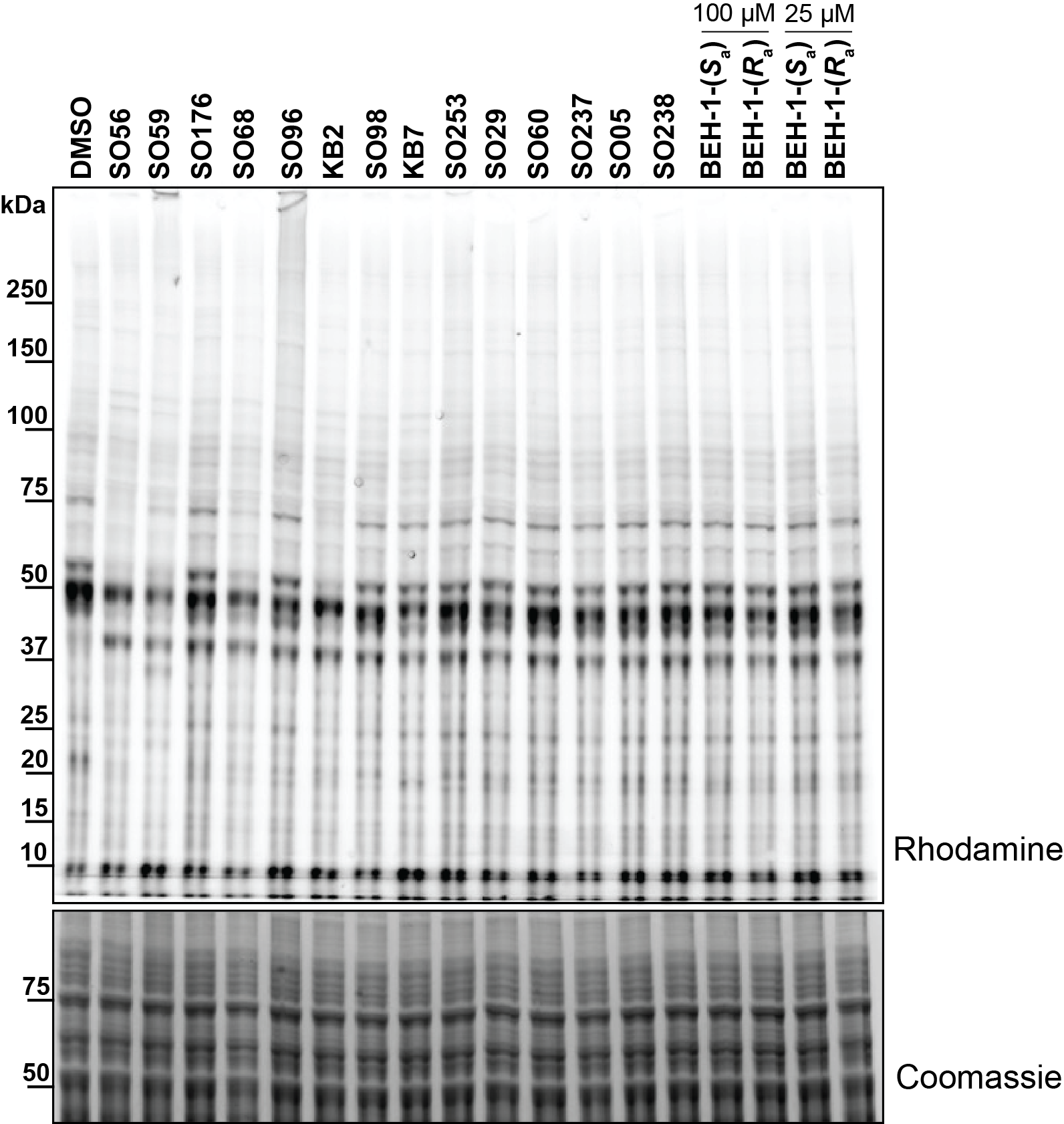


**Figure S13. General reactivity of each screened compound as assessed by in-gel fluorescence.** Jurkat whole cell lysates were subjected to the indicated compounds (500 µM, 1h, unless otherwise stated), followed by IA-Rhodamine (5 µM, 20 min), separation by SDS-PAGE gel and visualization by in-gel fluorescence and coomassie InstantBlue stain.


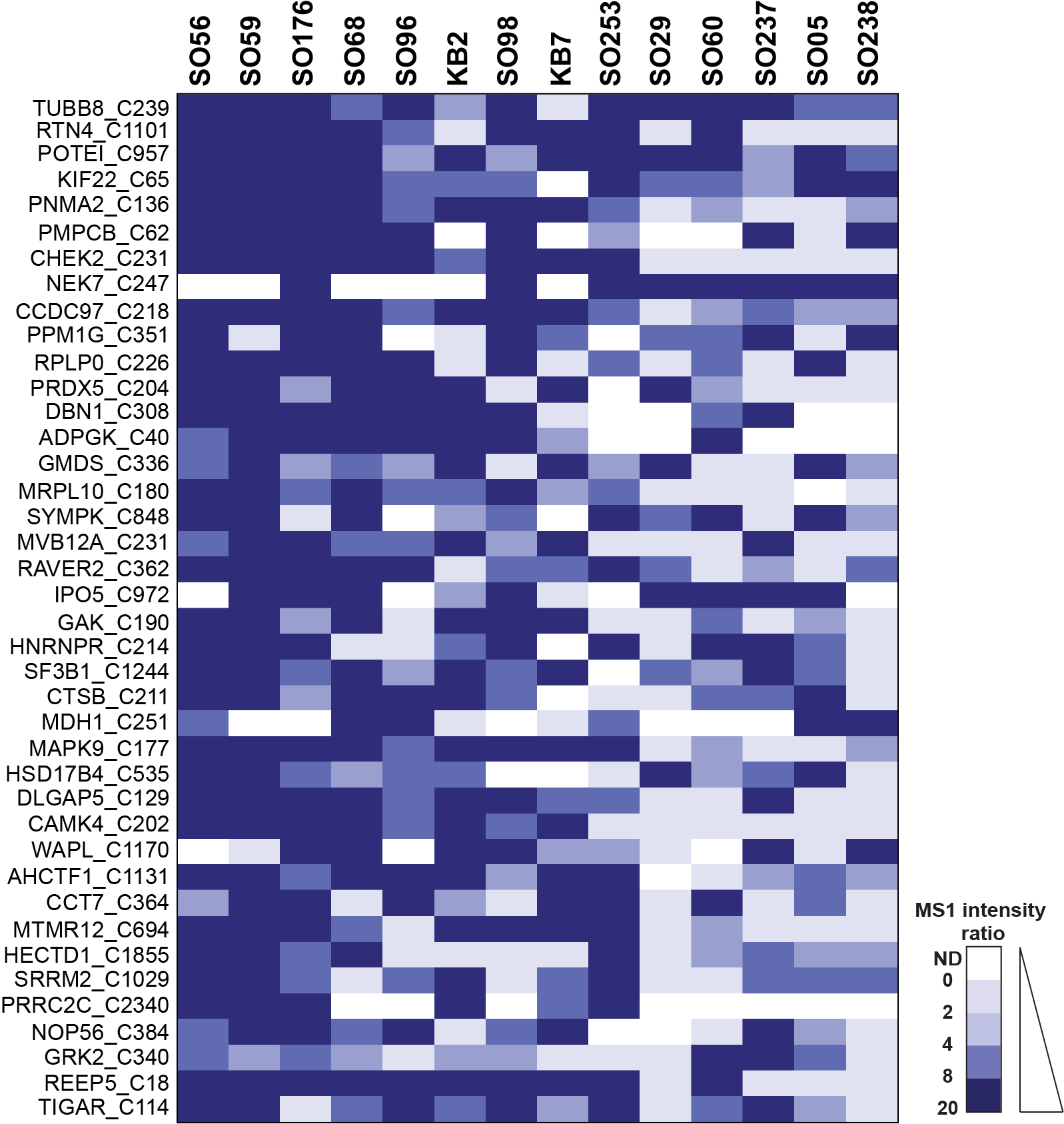


**Figure S14**. **Top reactive cysteines across compounds screened in plate.** All compounds treated at 500 µM (unless otherwise stated) for 1 hour in Jurkat whole cell lysates. Jurkat whole cell lysates were subjected to the indicated compounds (500 µM, 1h) followed by chemoproteomic sample preparation. All MS data is available in **Table S6.**


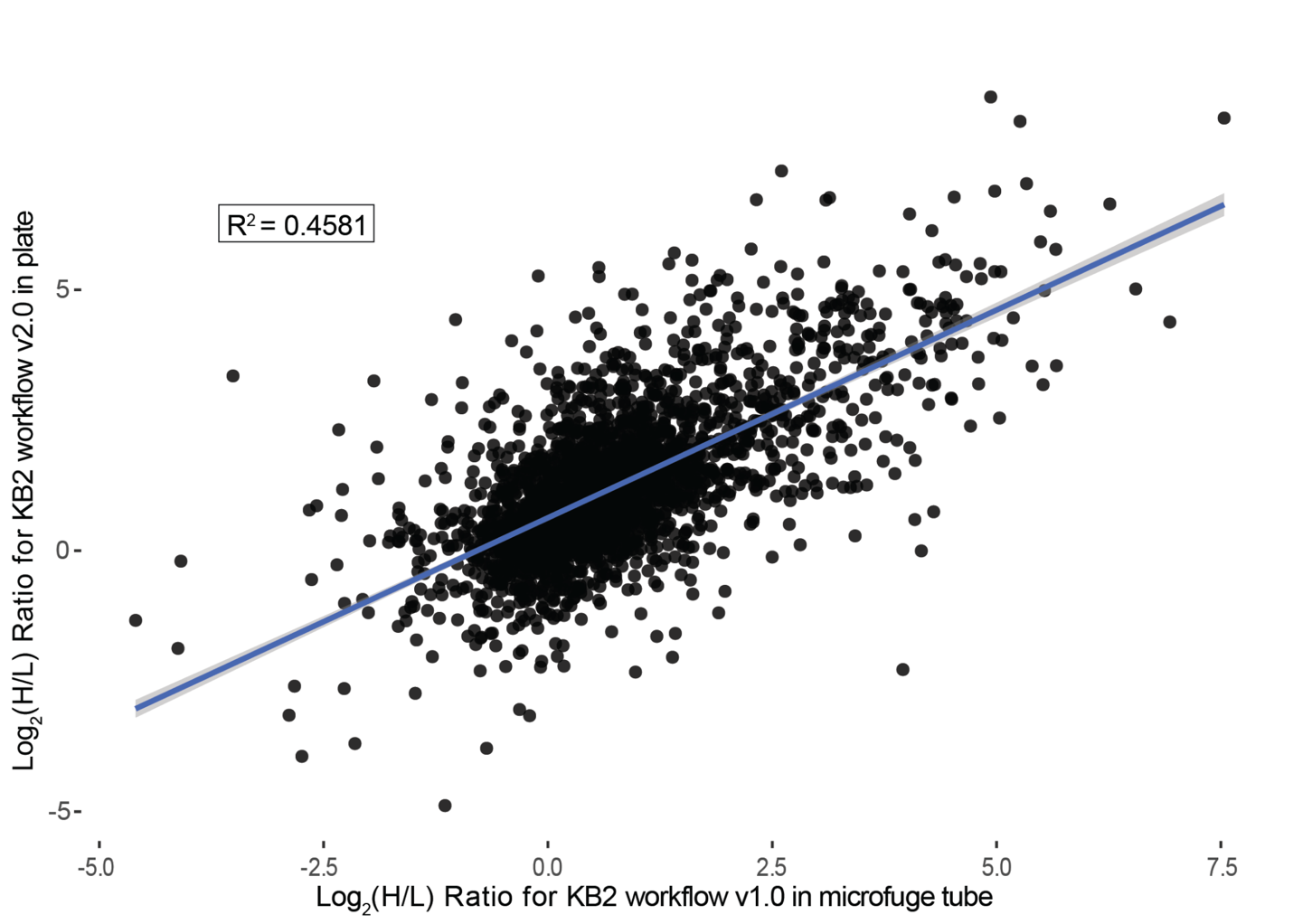


**Figure S15**. **Assessing concordance of ratios for samples prepared with plate workflow v2.0 versus microcentrifuge tube with workflow v1.0.** Jurkat cell whole cell lysate treated with KB2 (500 µM), comparing plate-generated data to workflow v1.0 in microfuge tube dataset previously shown in Figure 2. Data was filtered for cysteine identifiers present in both replicates and for each cysteine identifier ratio with a standard deviation of less than two. For workflow v1.0 prepared in a microcentrifuge tube, n=4. For workflow v2.0 in plate n=2. All MS data is available in **Table S5** (microfuge tube) and **Table S6** (plate).


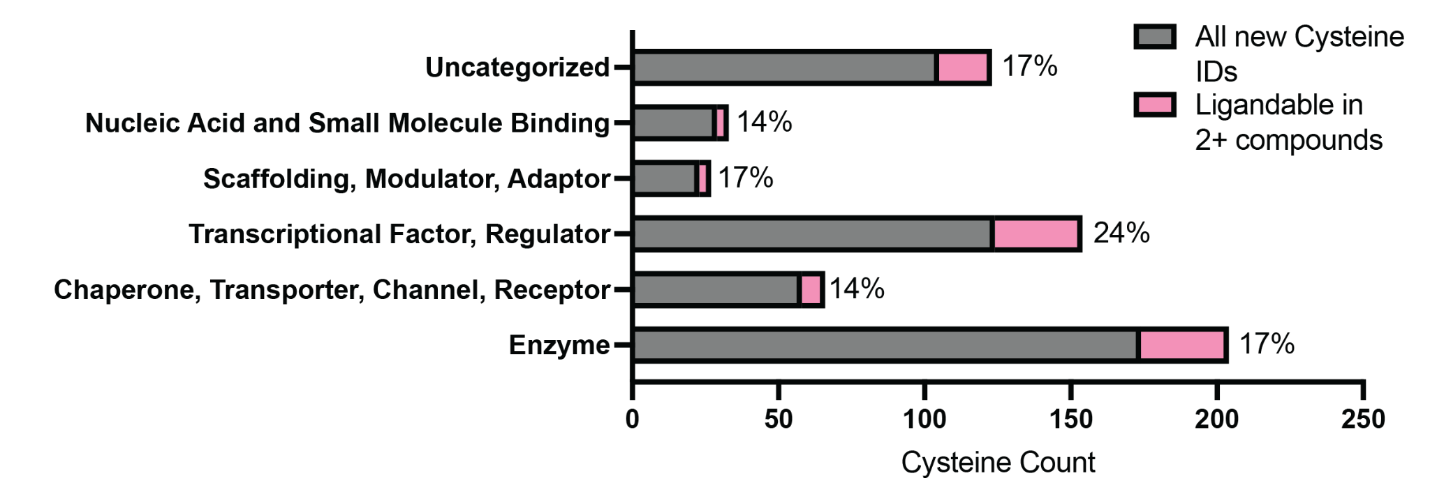


**Figure S16**. **Stratification of protein categories for cysteines not previously identified in CysDB**[^4^](https://sciwheel.com/work/citation?ids=14904677&pre=&suf=&sa=0)**.** Protein categorization was conducted as reported previously[^4^](https://sciwheel.com/work/citation?ids=14904677&pre=&suf=&sa=0) for all newly identified cysteines and the subset that are liganded (Log_2_(H/L) ratio ≥ 2) by 2 or more compounds. All MS data is available in **Table S6.**


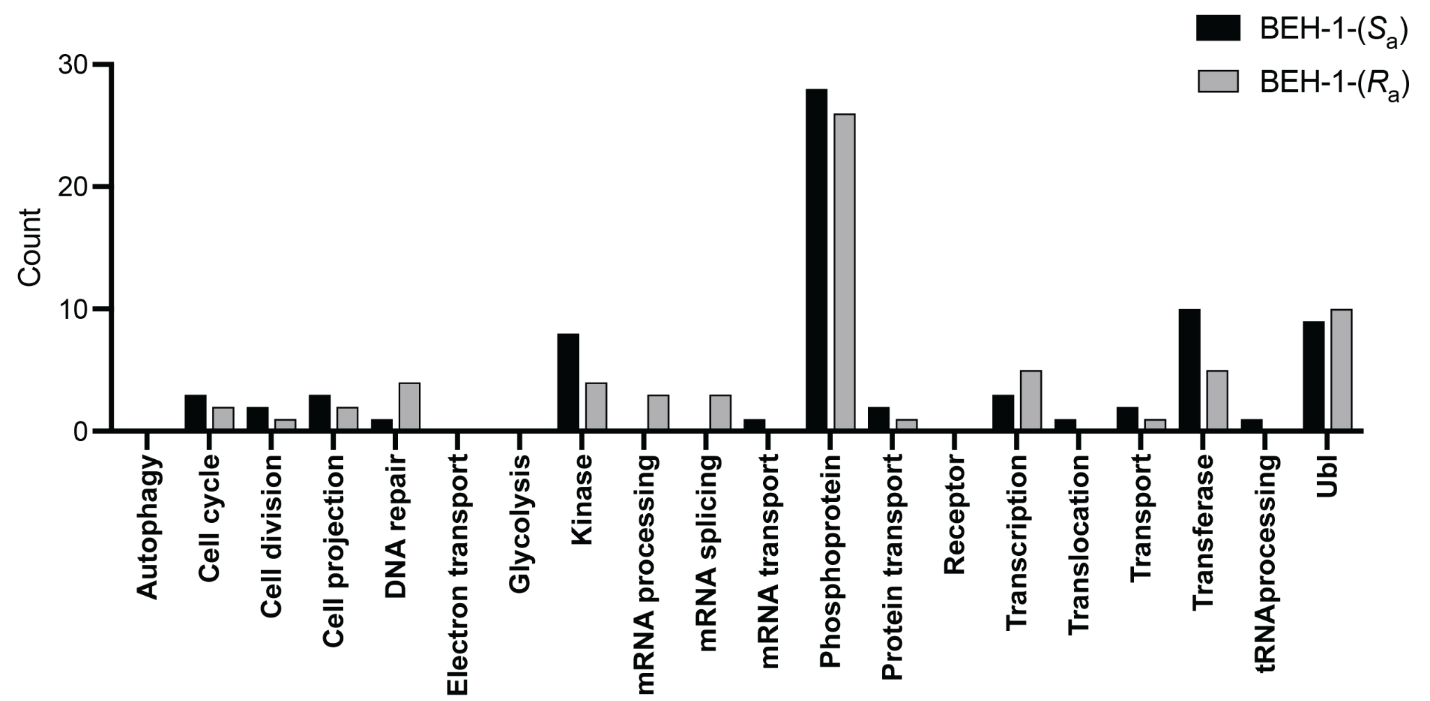


**Figure S17** **. UniProtKB annotations for types of proteins showing stereoselectivity with atropisomers BEH-1-(*S*_a_) and BEH-1-(*R*_a_).** Protein categorization based on UniprotKB annotations for cysteines liganded by either BEH-1-(*S*_a_) (black bars) or BEH-1-(*R*_a_) (gray bars). All MS data is available in **Table S6.**

**
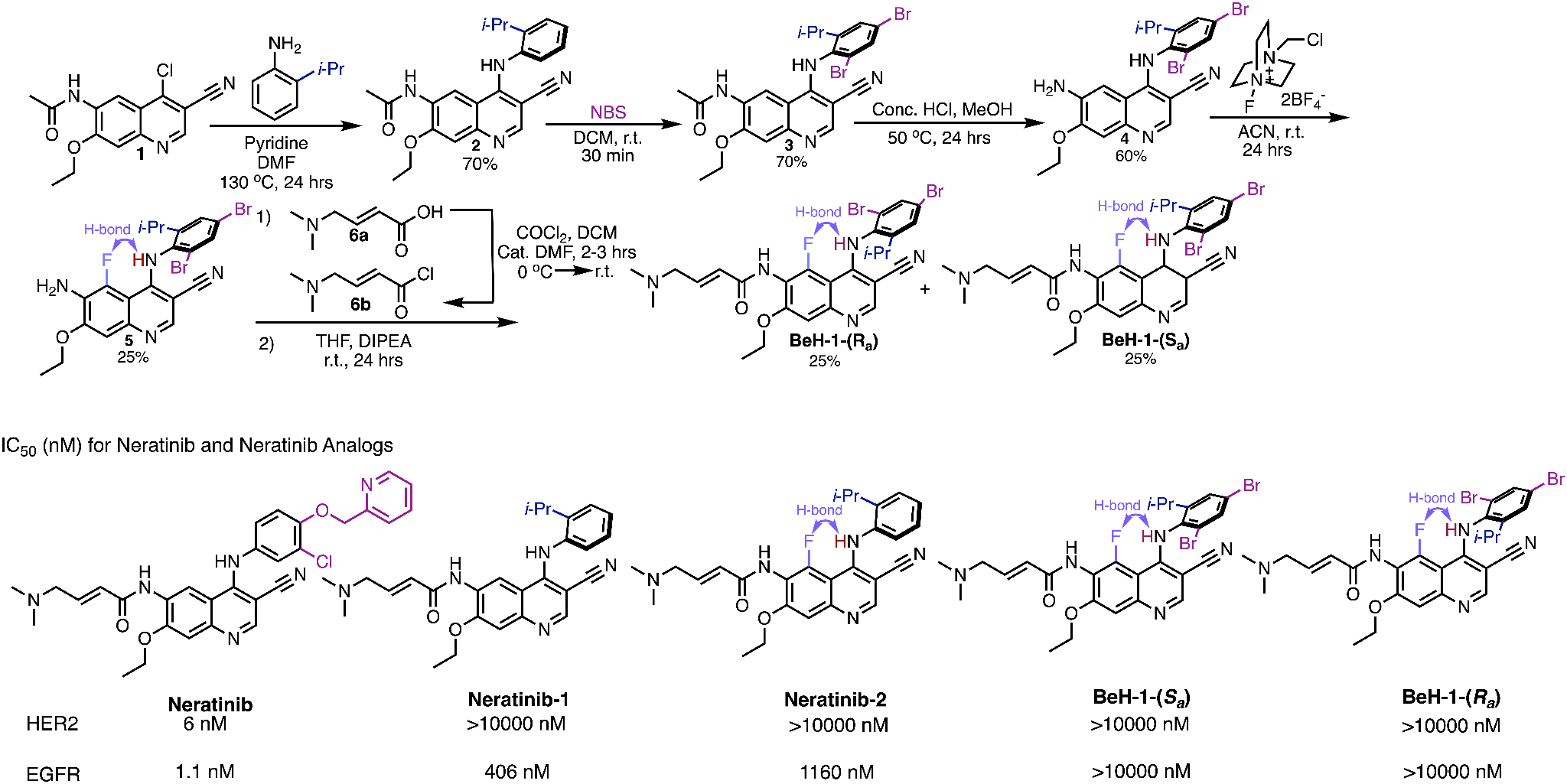
**

**Figure S18**. **IC_50_ values for Neratinib and analogs for known kinase targets.** The IC_50_ values for Neratinib’s primary targets were determined from the literature[^7^](https://sciwheel.com/work/citation?ids=11109253&pre=&suf=&sa=0). The rest of the IC_50_ values were obtained from Thermo Fisher Scientific using their Z^’^-LYTE assay.


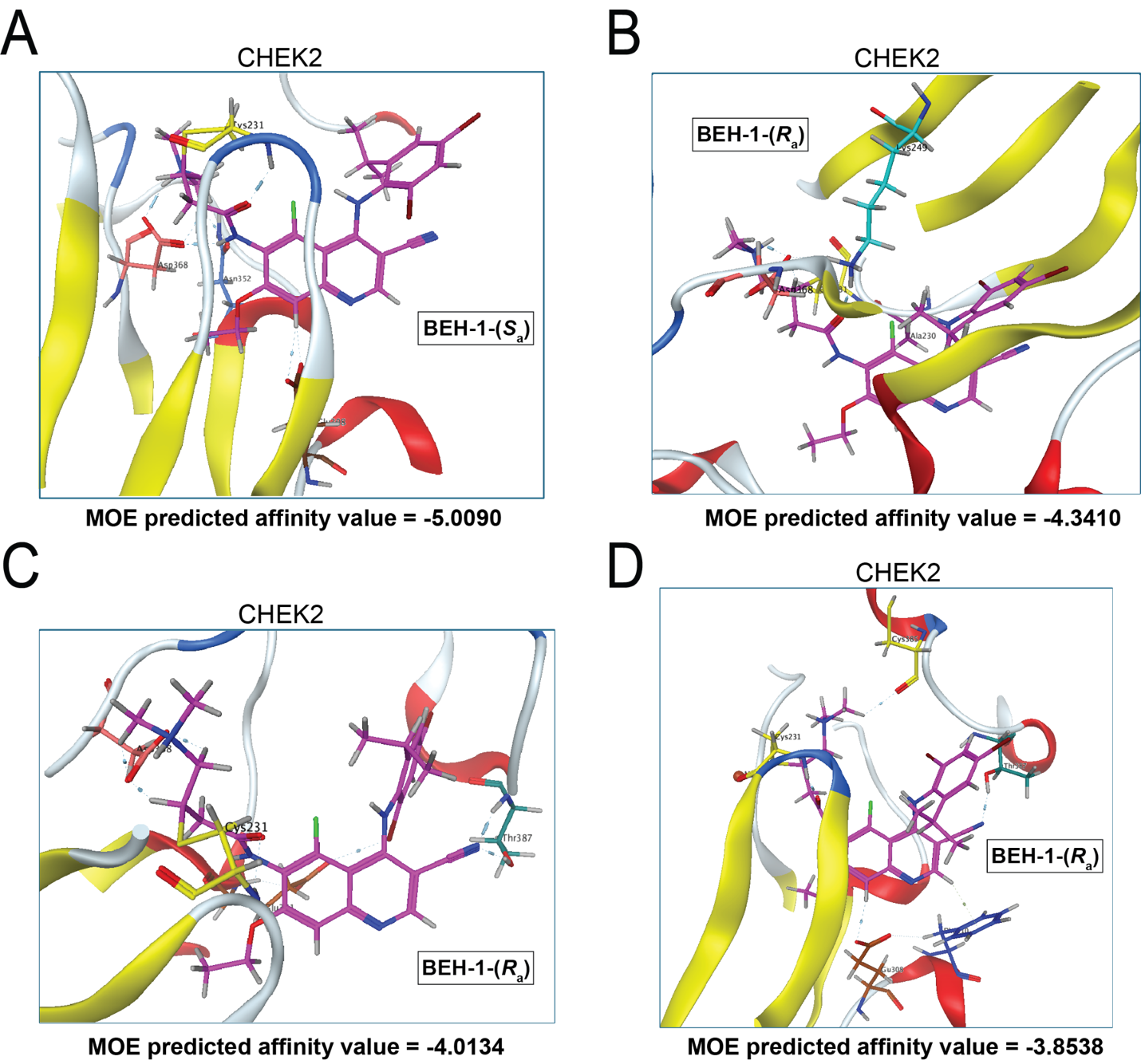


**Figure S19**. **Binding poses for the BEH-1 atropisomeric compound.** A) Binding pose showing the S atropisomer compound with an MOE predicted affinity value of -5.0090. BEH-1-(S_a_) is making a covalent bond with Cys231 in the active site (Glycine-rich loop) of CHEK2 along with various other hydrogen-bonding interactions in the hinge region and active site of the kinase. B) Binding pose showing the R atropisomer compound with an MOE predicted affinity value of -4.3410. BEH-1-(R_a_) is making a covalent bond with Cys231 in the active site of CHEK2 along with various other hydrogen-bonding interactions in the DFG motif and glycine-rich loop of the kinase. C) Binding pose showing the R atropisomer compound with an MOE predicted affinity value of -4.0134. BEH-1-(R_a_) is making a covalent bond with Cys231 in the active site of CHEK2 along with various other hydrogen-bonding and electrostatic interactions in the hinge region and active site of the kinase. D) Binding pose showing the R atropisomer compound with an MOE predicted affinity value of -3.8538. BEH-1-(R_a_) is making a covalent bond with Cys231 in the active site of CHEK2 along with various other hydrogen-bonding interactions in the DFG motif and active site of the kinase. All docking figures generated with CHEK2 PDB structure 2XBJ in MOE 2020.


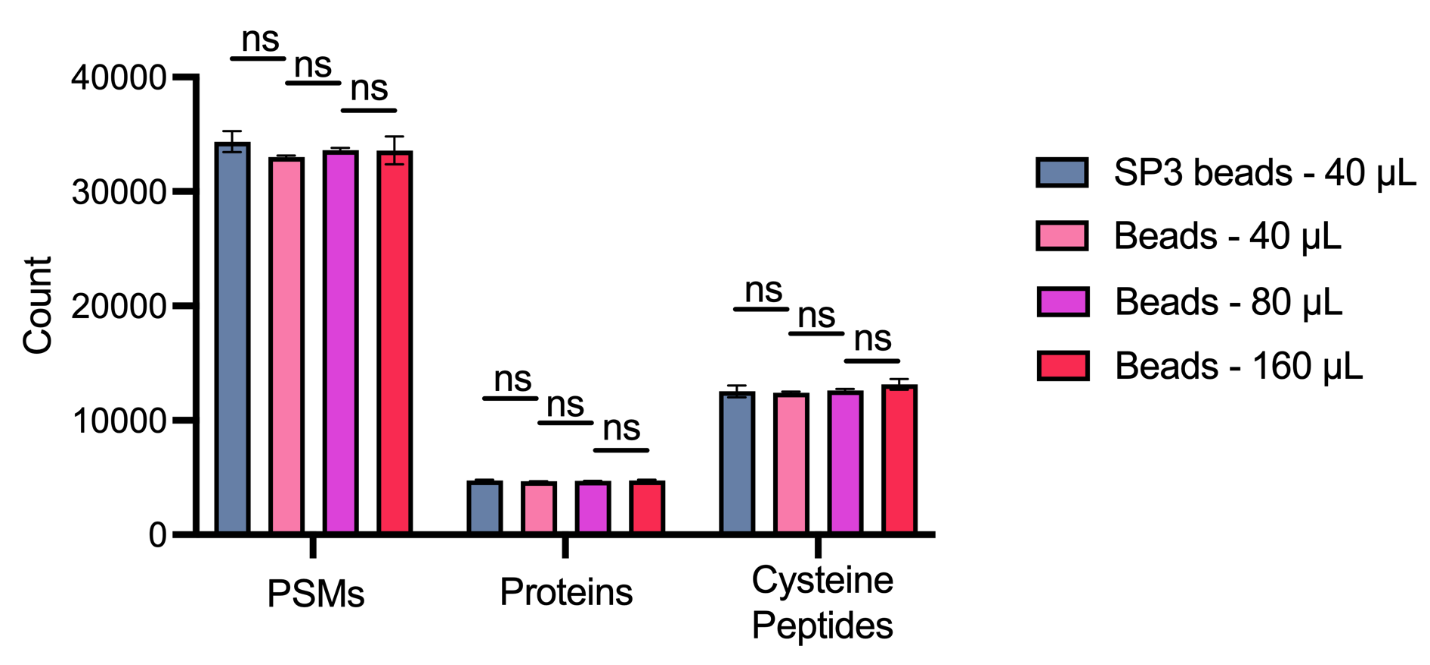


**Figure S20**. **Comparing cysteine chemoproteomic coverage obtained with standard SP3 resin versus generic DNA cleanup magnetic** **beads.** Chemoproteomic samples were prepared using Jurkat whole cell lysates capped with IAA (200 µM) clicked to biotin azide were subjected to chemoproteomic sample preparation in microcentrifuge tubes (n=2/condition) using the indicated volumes of resin, following the workflow v2.0, with digested peptides directly enriched on neutravidin beads after and resin protein cleanup. Statistical significance was calculated by performing Student’s t-test. p < 0.05 for significance cutoff, n=2. All MS data is available in **Table S7**.


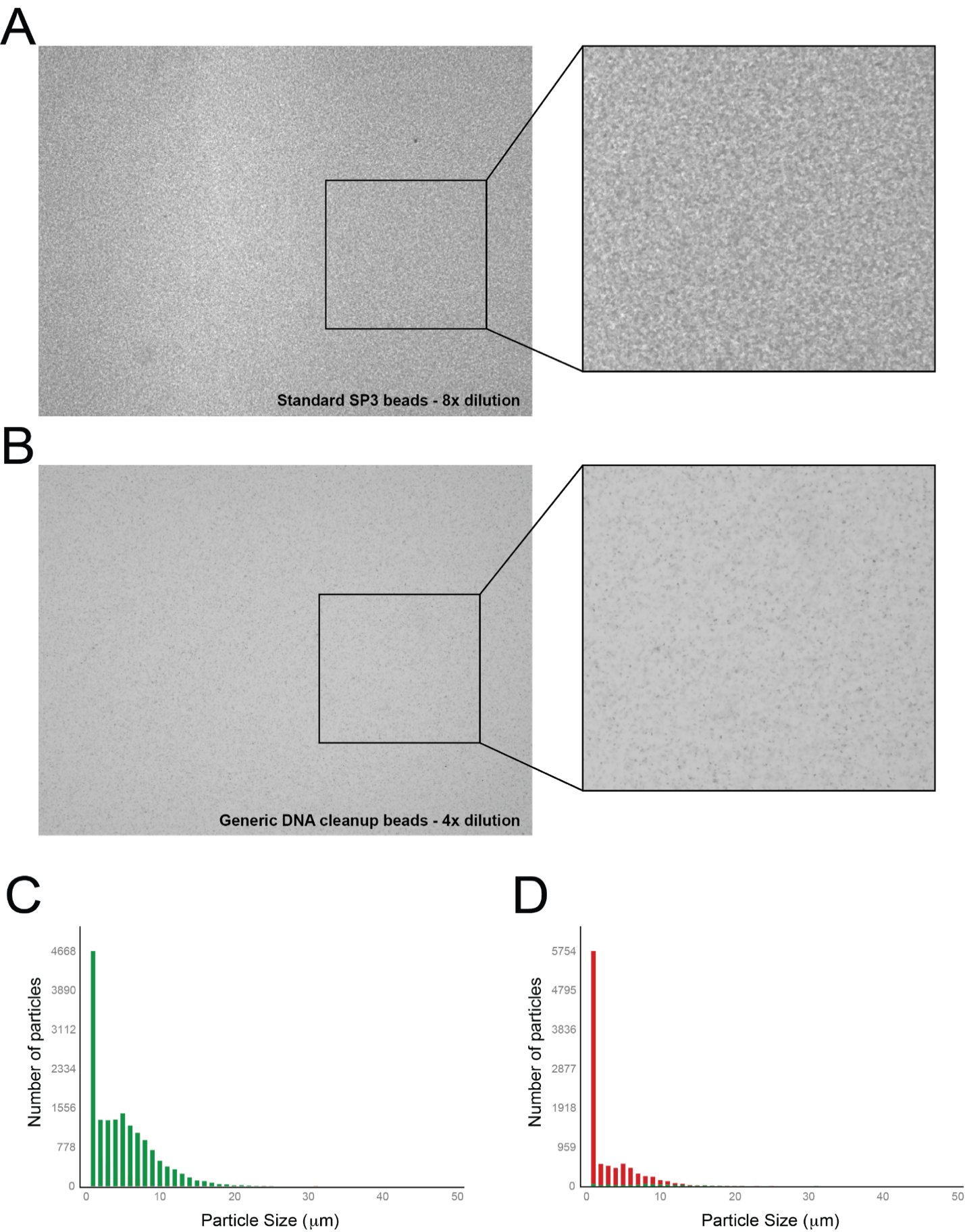


**Figure S21. Comparison of bead size and concentration using Countess.** A) Standard SP3 beads diluted 8x (10 µL bead slurry in 80 µL water) and imaged using Countess. B) Generic DNA cleanup beads diluted 4x (10 µL bead slurry in 40 µL water) and imaged using Countess C) Particle size analysis for standard SP3 beads diluted 8xas generated by Countess. D) Particle size analysis for generic DNA cleanup beads diluted 4x as generated by Countess.


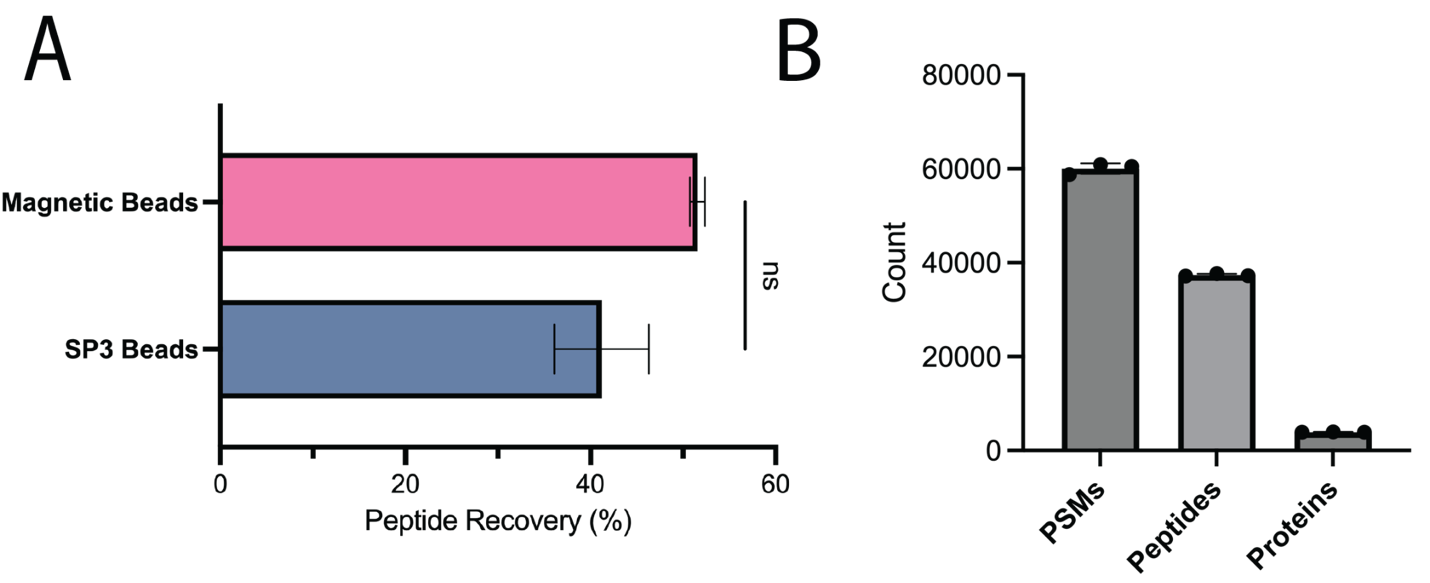


**Figure S22**. **Coverage for protein and peptide cleanup done with generic DNA cleanup magnetic beads.** A) Jurkat whole cell lysates (200 µg) subjected to protein and peptide cleanup with standard SP3 magnetic beads and generic DNA cleanup magnetic beads. Percent recovery calculated based on total µg of peptides measured post-digest and pre-peptide cleanup. Peptide concentrations were measured pre- and post-cleanup for calculation of peptide recovery, using the Pierce Quantitative Peptide Concentration Assay. Statistical significance was calculated by performing Student’s t-test. p < 0.05 for significance cutoff, n=2. B) HEK293T whole cell lysates subjected to protein- and peptide-cleanup with on-resin digest, using generic DNA cleanup magnetic beads, followed by LC-MS/MS analysis. All MS data is available in **Table S7**.


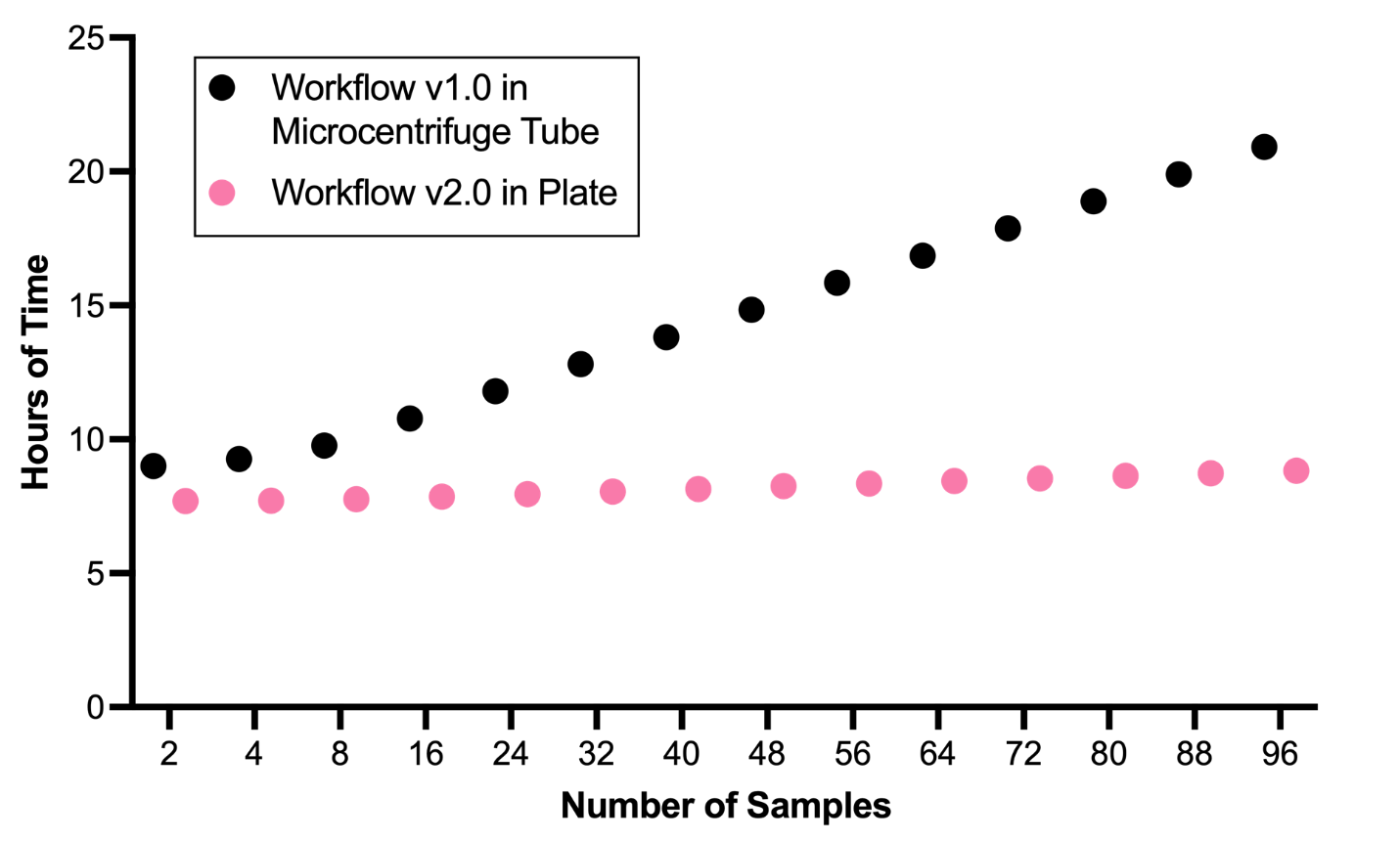


**Figure S23.** **Estimated time saving with CySP-96 as compared to cysteine chemoproteomic preparation Workflow 1.0 in microcentrifuge tubes.** Data calculated based on the time taken to prepare 2-8 samples.

# Supplementary Tables

**Table S1.** Scaling of digest volumes. Table shows the relationship of digest volume adjustments as it relates to digest concentration and subsequent acetonitrile volumes to achieve ≥95% acetonitrile v/v for optimal peptide binding to SP3 beads.

| Digest Volume (µL) | Digest Concentration (mg/mL) | Acetonitrile Volume to add (µL) | Total Volume of Peptide Binding (µL) |
| --- | --- | --- | --- |
| 75 µL | 2.67 | 1800 µL | 1895 µL |
| 50 µL | 4.00 | 1280 µL | 1350 µL |
| 25 µL | 8.00 | 640 µL | 685 µL |
| 20 µL | 10.00 | 510 µL | 550 µL |
| 15 µL | 13.33 | 380 µL | 415 µL |

**Table S2.** Total volumes for each plate step.

*Volumes should be maintained below 1200 µL, as smaller volumes improve magnetic bead separation.

| Step | Volume Range (µL) | Plate |
| --- | --- | --- |
| Lysate treatment | 100-200  (compound + IAA ~4-6µL additional) | First plate |
| CuAAC | ~130-230 |  |
| 1% SDS + benzonase | ~150-250 |  |
| Combine heavy + light | ~300-500 |  |
| Add SP3/magnetic beads + >50% EtOH | ~700-1200* |  |
| 80% EtOH washes | ~200-400 |  |
| DTT/IA | ~100 |  |
| Trypsin Digest | 100-150 |  |
| Avidin Enrichment | ~500 | Second plate |
| Washes | ~500 |  |
| Elution | 60/elution |  |

**Table S3.** Conditions of Liquid-chromatography (LC)

| Experiment Type | Parameter | Condition |
| --- | --- | --- |
| isoTOP-ABPP  Plate screen | Column | 100 μM ID fused silica capillary packed in-house with bulk C18 reversed phase resin (particle size, 1.9 μm; pore size, 100 Å; Dr. Maisch GmbH) |
|  | Mobile phase | Buffer A: water with 3% DMSO and 0.1% formic acid  Buffer B: 80% acetonitrile with 3% DMSO and 0.1% formic acid |
|  | Gradient and flow rate | 1 – 80 min, 6 – 40% B, 300 nL/min |
|  |  | 80 – 89 min, 40 – 55% B, 300 nL/min |
|  |  | 89 – 90 min, 55 – 95% B, 300 nL/min |
|  |  | 90 – 99 min, 95% B, 300 nL/min |
|  |  | 99 – 100 min, 1% B, 300 nL/min |
|  |  | 99 – 120 min, 1% B, 300 nL/min |
|  | Run time | 120 minutes |
|  | Injection volume | 5 uL |
| isoTOP-ABPP  All workflow optimization experiments | Column | 100 μM ID fused silica capillary packed in-house with bulk C18 reversed phase resin (particle size, 1.9 μm; pore size, 100 Å; Dr. Maisch GmbH) |
|  | Mobile phase | Buffer A: water with 3% DMSO and 0.1% formic acid  Buffer B: 80% acetonitrile with 3% DMSO and 0.1% formic acid |
|  | Gradient and flow rate | 0 – 5 min, 3 – 10% B, 300 nL/min |
|  |  | 5 – 15 min, 10 - 20% B, 220 nL/min |
|  |  | 15 – 64 min, 20 - 47% B, 220 nL/min |
|  |  | 64 – 70 min, 47 – 95% B, 250 nL/min |
|  | Run time | 70 minutes |
| Sergi-bead cleanup | Column | 100 μM ID fused silica capillary packed in-house with bulk C18 reversed phase resin (particle size, 1.9 μm; pore size, 100 Å; Dr. Maisch GmbH) |
|  | Mobile phase | Buffer A: water with 3% DMSO and 0.1% formic acid  Buffer B: 80% acetonitrile with 3% DMSO and 0.1% formic acid |
|  | Gradient and flow rate | 1 – 80 min, 3 – 40% B, 300 nL/min |
|  |  | 80 – 89 min, 40 – 55% B, 300 nL/min |
|  |  | 89 – 90 min, 55 – 95% B, 300 nL/min |
|  |  | 90 – 99 min, 95% B, 300 nL/min |
|  |  | 99 – 100 min, 1% B, 300 nL/min |
|  |  | 99 – 120 min, 1% B, 300 nL/min |
|  | Run time | 120 minutes |

Table S4: Figure 1 MS data

Table S5: Figure 2 MS data

Table S6: Figures 3 + 5 MS data

Table S7: Figure 6 MS data

Table S8: MS raw file annotation

# Chemical Synthesis

**General Methods.** All solution-phase reactions were performed in oven-dried glassware under an atmosphere of dry N_2_ except where water was used as a solvent. Reactions were monitored by thin-layer chromatography, and plates were visualized by fluorescence quenching under UV light or by staining with iodine. Concentration *in vacuo* refers to the removal of solvent on a Heidolph rotary evaporator under reduced pressure.

For compounds requiring purification, manual silica gel column chromatography was carried out using SiliCycle silica gel P60 as the stationary phase. Typical stationary phase column loading was a column height of 250 mm, a column diameter of 100 mm. Typical eluents used were hexanes/ethyl acetate, methanol/CH_2_Cl_2_ , petroleum ether/ethyl acetate or hexanes/CH_2_Cl_2_.

Other reagents were purchased from Sigma-Aldrich (St. Louis, MO), Alfa Aesar (Ward Hill, MA), EMD Millipore (Billerica, MA), Fisher Scientific (Hampton, NH), Oakwood Chemical (West Columbia, SC), Combi-Blocks (San Diego, CA) and Cayman Chemical (Ann Arbor, MI) and used without further purification. ^1^H NMR and ^13^C NMR spectra for characterization of new compounds and monitoring reactions were collected in CDCl_3_, CD_3_OD, or DMSO-*d_6_* (Cambridge Isotope Laboratories, Cambridge, MA) on a Bruker AV 400 MHz spectrometer or Bruker AV 500 MHz in the Department of Chemistry & Biochemistry at University of California, Los Angeles. All chemical shifts were reported in the standard notation of parts per million using the peak of residual proton signals of the deuterated solvent as an internal reference. Coupling constant (*J*) units are in Hertz (Hz) to the nearest 0.1 Hz. Splitting patterns were indicated as follows: br, broad; s, singlet; d, doublet; t, triplet; q, quartet; m, multiplet; dd, doublet of doublets; dt, doublet of triplets. Low-resolution mass spectroscopy was performed on an Agilent Technologies InfinityLab LC/MSD single quadrupole LC/MS (ESI source).

Atropisomeric compounds BEH-1-(*S*_a_) and BEH-1-(*R*_a_) and fragments SO-05 to SO-253 used in this study were all synthesized as described below:

Materials and General Methods:

^1^H and ^13^C NMR spectra were recorded on the Varian VNMRS 400 MHz and 400 MHz Bruker Avance AV1 spectrometers at 25 ⁰C. All chemical shifts were reported in parts per million (ppm) and were internally referenced to residual protio solvents unless otherwise noted. Fluorine spectra were referenced to an external TFA standard. Spectral data were reported as follows: chemical shift (multiplicity [singlet (s), doublet (d), triplet (t), quartet (q), pentet (p), and multiplet (m)], coupling constants [Hz], integration). Carbon spectra were recorded with complete proton decoupling. Conventional mass spectra were obtained using Advion Expression CMS (APCI and ASAP). All chemicals used in the synthesis of substrates were purchased from Sigma Aldrich, TCI, Frontier Scientific, Acros Organics, Combi Blocks, Oakwood, Matrix Scientific, or Fisher Scientific and were used as received without further purification, unless specifically noted. All normal phase flash column chromatography (FCC) was performed using Grade 60 Silica Gel (230-400 mesh) purchased from Fisher Scientific or performed on a Biotage Isolera One with a Biotage SNAP cartridge (KP-SIL- 10-100g). CHIRALPAK ID and semipreparative CHIRALPAK ID columns were used from Daicel Technologies Corporation to separate the final compound.

Preparation of BEH-1:

**Synthesis of *N*-(3-cyano-7-ethoxy-4-((2-isopropylphenyl)amino)quinolin-6-yl)acetamide (2)**: *N*-(4-chloro-3-cyano-7-ethoxyquinolin-6-yl)acetamide (**1**, 3 g, 10.4 mmol, 1.0 eq) was added to a 75 mL pressure vessel, followed by 2-isopropylaniline (2.2 mL, 15.5 mmol. 1.5 eq), pyridine (1.7 mL, 20.7 mmol, 2.0 eq), and DMF (34.5 mL, 0.3M) at room temperature. The reaction mixture was stirred overnight at 130 °C. The reaction mixture was allowed to cool to room temperature and was diluted with ethyl acetate (30 mL) and washed with water (30x10 mL) and brine (30 mL). The organic layer was dried with anhydrous sodium sulfate, concentrated *in vacuo*, and purified using flash silica gel column chromatography (gradient up to 65% ethyl acetate in hexanes) to give *N*-(3-cyano-7-ethoxy-4-((2-isopropylphenyl)amino)quinolin-6-yl)acetamide (**2**, 2.8 g, 70% yield) as a beige solid.

**^1^H NMR** (400 MHz, CDCl_3_) δ 9.07 (s, 1H), 8.46 (s, 1H), 8.06 (s, 1H), 7.42 (m, *J* = 4.9, 2.1 Hz, 2H), 7.38 (s, 1H), 7.24 (dd, *J* = 5.9, 2.7 Hz, 1H), 7.19 (d, *J* = 7.8 Hz, 1H), 4.31 (q, *J* = 7.0 Hz, 2H), 3.19 (m, *J* = 6.9 Hz, 1H), 2.27 (s, 3H), 1.57 (t, *J* = 6.9 Hz, 3H), 1.27 (d, *J* = 6.9 Hz, 6H).

**^13^C NMR** (101 MHz, CDCl_3_) δ 168.59, 152.38, 151.25, 150.68, 146.25, 146.00, 135.30, 128.83, 128.03, 127.79, 126.52, 126.45, 116.32, 112.18, 108.75, 108.30, 86.50, 64.90, 28.21, 24.99, 23.06, 14.35.

**MS (APCI):** Calculated for C_23_H_24_N_4_O_2_ [M+H]^+^: 389.19; Found: 389.1982 m/z

^1^H NMR of ***N*-(3-cyano-7-ethoxy-4-((2-isopropylphenyl)amino)quinolin-6-yl)acetamide (2)** in CDCl_3_


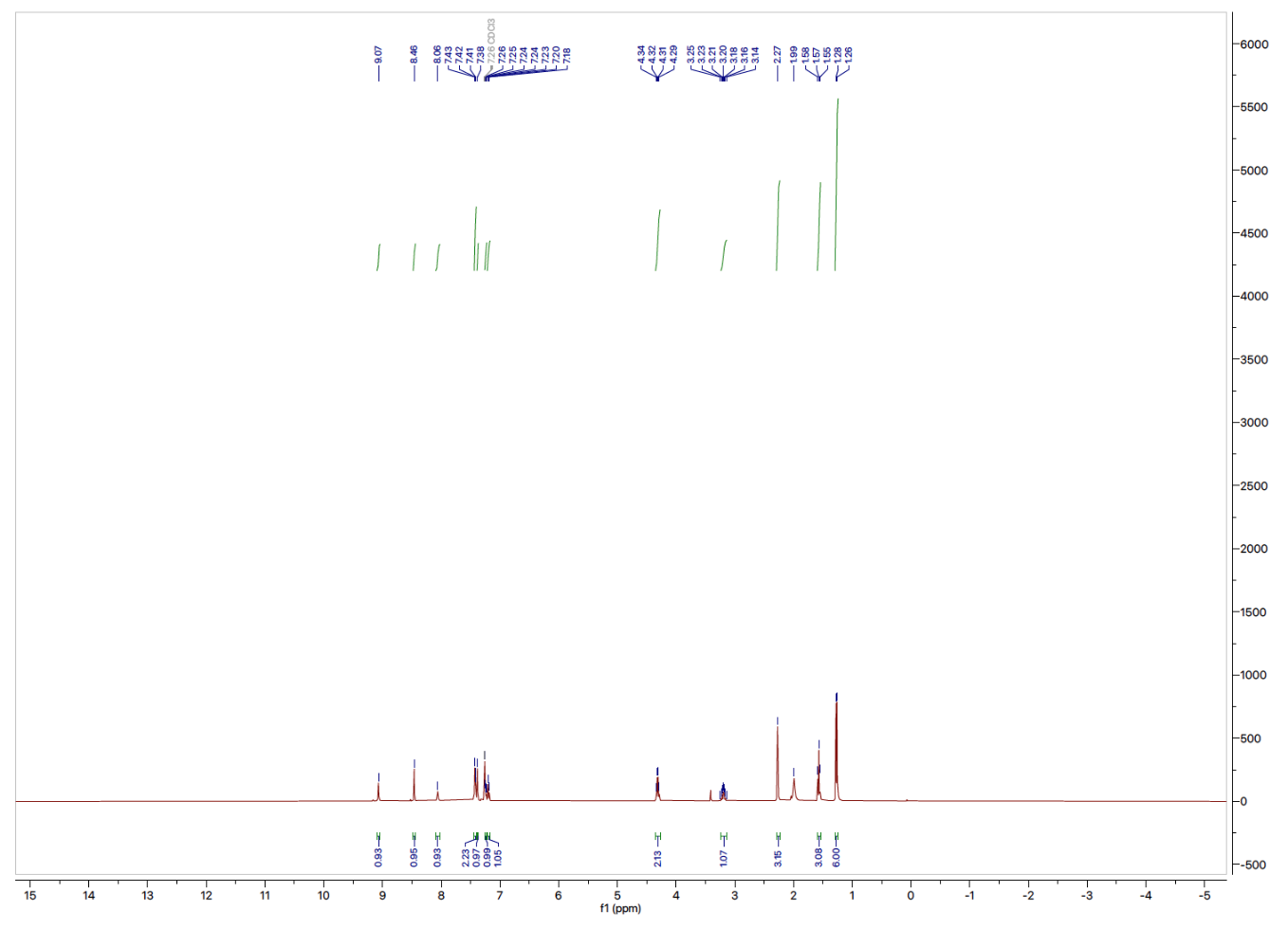


^13^C NMR of ***N*-(3-cyano-7-ethoxy-4-((2-isopropylphenyl)amino)quinolin-6-yl)acetamide (2)** in CDCl_3_


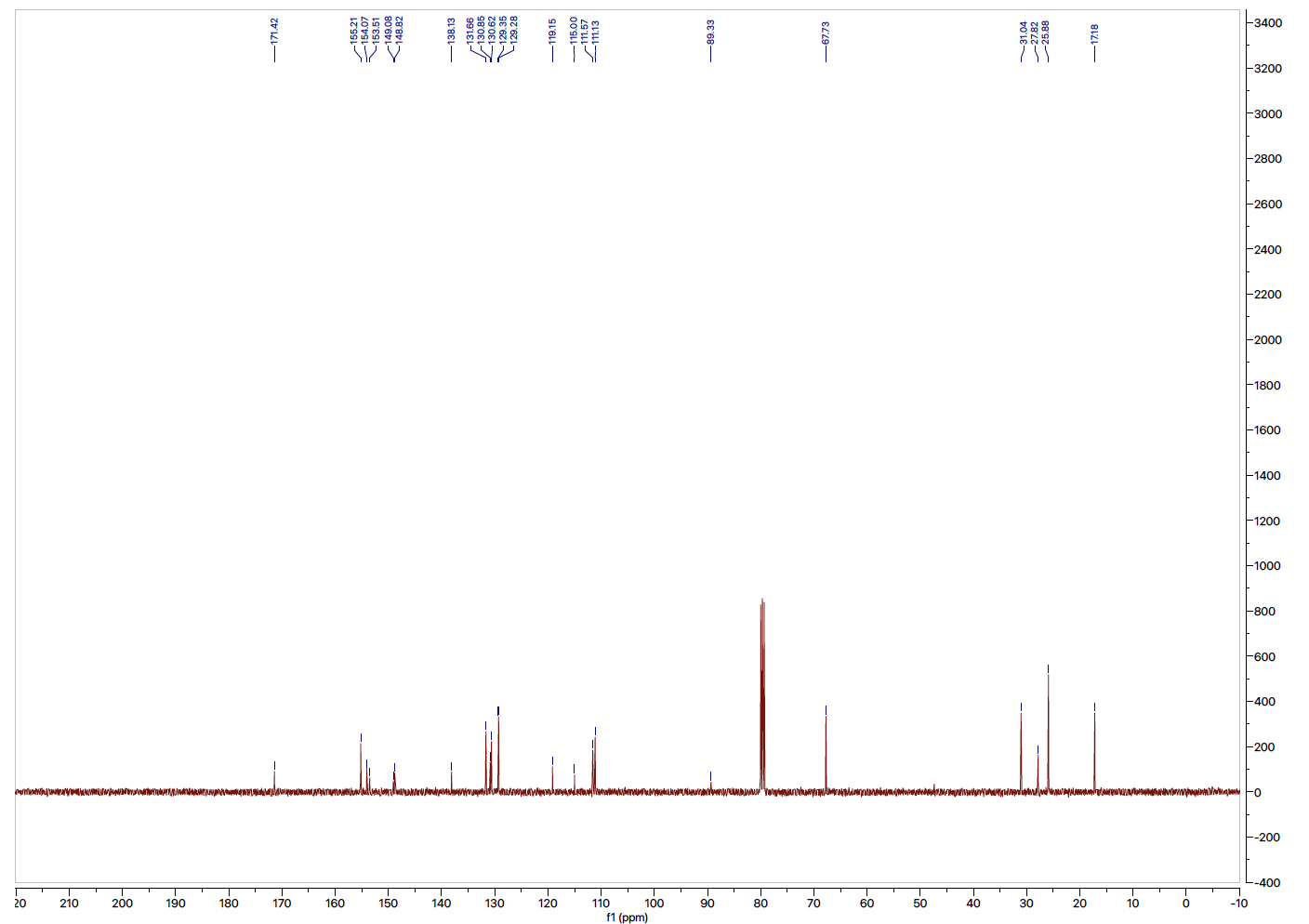


**Synthesis of *N*-(3-cyano-4-((2,4-dibromo-6-isopropylphenyl)amino)-7-ethoxyquinolin-6-yl)acetamide (3)**: *N*-(3-cyano-7-ethoxy-4-((2-isopropylphenyl)amino)quinolin-6-yl)acetamide (**2**, 800 mg, 2.1 mmol, 1.0 eq) was added to a 15 mL screw cap glass vial, followed by DCM (6.9 mL, 0.3 M) and *N*-Bromosuccinimide (366.6 mg, 4.3 mmol, 2.1 eq). The reaction was allowed to stir at room temperature for 30 minutes and was extracted with DCM (30 mL), washed with sat. NaHCO_3_ (3x30 mL), and brine (30 mL). The organic layer was dried with anhydrous sodium sulfate, concentrated *in vacuo*, and purified using flash silica gel column chromatography (gradient up to 60% ethyl acetate in hexanes) to give *N*-(3-cyano-4-((2,4-dibromo-6-isopropylphenyl)amino)-7-ethoxyquinolin-6-yl)acetamide (**3**, 560 mg, 70% yield) as a beige solid.

**^1^H NMR** (400 MHz, CDCl_3_) δ 9.14 (s, 1H), 8.48 (s, 1H), 8.09 (s, 1H), 7.69 (d, *J* = 2.3 Hz, 1H), 7.48 (d, *J* = 2.1 Hz, 1H), 7.45 (s, 1H), 4.33 (q, *J* = 7.0 Hz, 2H), 3.14 (m, *J* = 6.9 Hz, 1H), 2.29 (s, 3H), 1.58 (t, *J* = 7.0 Hz, 3H), 1.23 (d, *J* = 6.9 Hz, 3H), 1.19 (d, *J* = 6.9 Hz, 3H).

**^13^C NMR** (101 MHz, CDCl_3_) δ 171.55, 154.85, 154.52, 153.69, 153.10, 135.58, 135.36, 131.89, 131.22, 128.75, 126.56, 118.65, 114.48, 111.05, 111.10, 88.66, 67.87, 32.43, 27.85, 26.38, 25.32, 17.17.

**MS (APCI):** Calculated for C_23_H_22_Br_2_N_4_O_2_ [M+H]^+^: 547.26 m/z; Found: 547.0180 m/z

^1^H NMR of ***N*-(3-cyano-4-((2,4-dibromo-6-isopropylphenyl)amino)-7-ethoxyquinolin-6-yl)acetamide (3)** in CDCl_3_


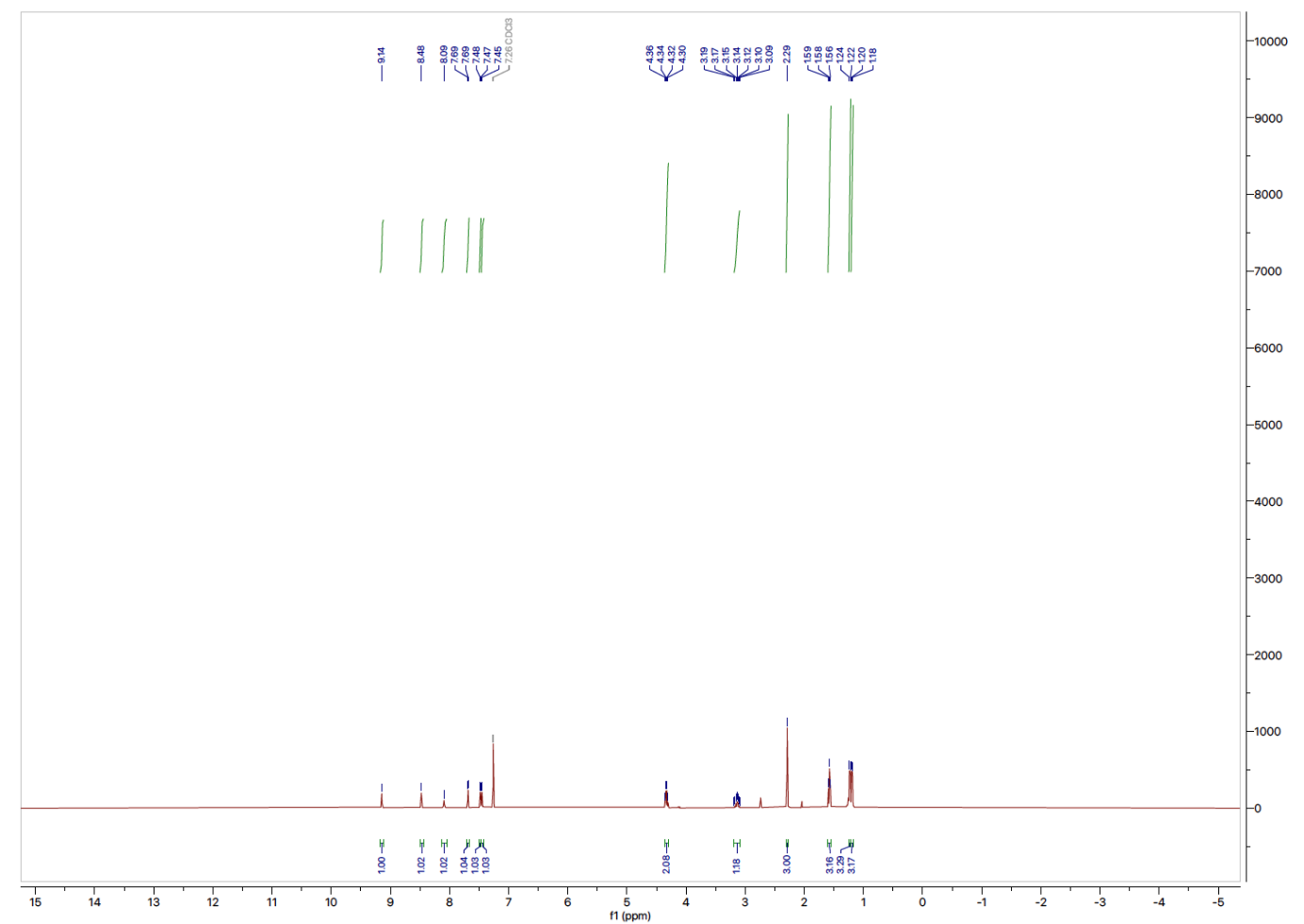


^13^C NMR of ***N*-(3-cyano-4-((2,4-dibromo-6-isopropylphenyl)amino)-7-ethoxyquinolin-6-yl)acetamide (3)** in CDCl_3_


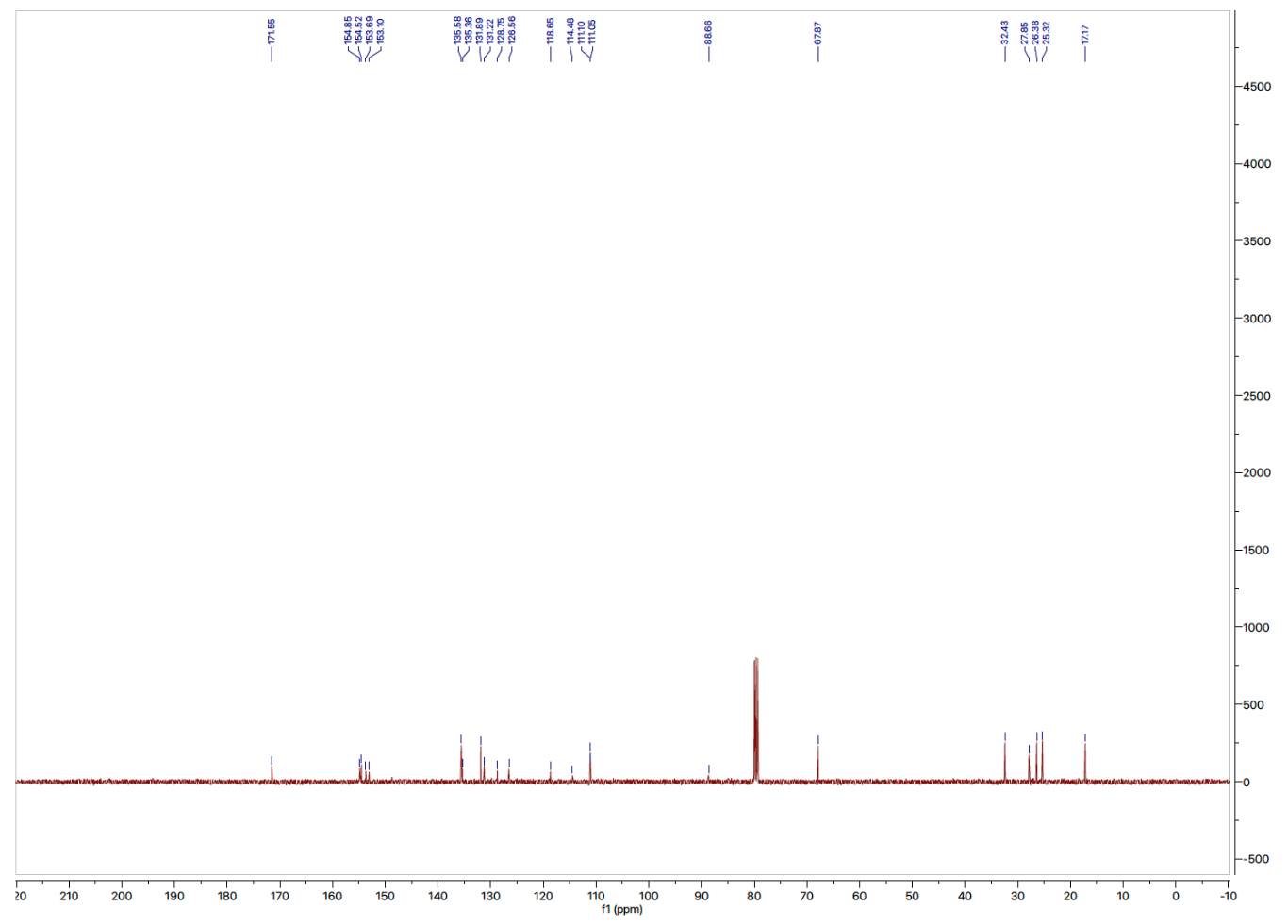


**Synthesis of 6-amino-4-((2,4-dibromo-6-isopropylphenyl)amino)-7-ethoxyquinoline-3-carbonitrile**

**(4)**. *N*-(3-cyano-4-((2,4-dibromo-6-isopropylphenyl)amino)-7-ethoxyquinolin-6-yl)acetamide (**3**, 1 g, 2.6 mmol, 1.0 eq) was added to a 100 mL round-bottom flask followed by methanol (25.7 mL, 0.1 M), and 6N HCl (4.4 mL, 0.58M). The reaction mixture was stirred overnight at 70 °C. The reaction mixture was then allowed to cool to room temperature and was extracted in ethyl acetate (30 mL), quenched with sat. NaHCO_3_ (3x30 mL), and washed with brine (30 mL). The organic layer was dried with anhydrous sodium sulfate, concentrated *in vacuo*, and purified using flash silica gel column chromatography (gradient up to 50% ethyl acetate in hexanes) to give 6-amino-4-((2,4-dibromo-6-isopropylphenyl)amino)-7-ethoxyquinoline-3-carbonitrile (**4**, 650 mg, 60% yield) as a bright yellow solid.

**^1^H NMR** (400 MHz, CDCl_3_) δ 8.38 (s, 1H), 7.69 (d, *J* = 2.1 Hz, 1H), 7.47 (d, *J* = 2.3 Hz, 1H), 7.28 (s, 1H), 6.95 (s, 1H), 6.53 (s, 1H), 4.29 (s, 1H), 4.25 (q, *J* = 7.0 Hz, 2 H), 3.12 (m, *J* = 6.7 Hz, 1H), 1.53 (t, *J* = 7.0 Hz, 3H), 1.24 (d, *J* = 6.8 Hz, 3H), 1.16 (d, *J* = 6.8 Hz, 3H).

**^13^C NMR** (101 MHz, CDCl_3_) δ 154.36, 154.11, 152.17, 151.07, 140.52, 136.05, 135.60, 131.87, 128.42, 125.96, 119.18, 115.47, 111.34, 102.86, 89.13, 67.16, 32.33, 26.57, 25.13, 17.23.

**MS (APCI):** Calculated for C_21_H_20_Br_2_N_4_O [M+H]^+^: 505.23 m/z; Found: 505.0071 m/z

^1^H NMR of **6-amino-4-((2,4-dibromo-6-isopropylphenyl)amino)-7-ethoxyquinoline-3-carbonitrile**

**(4)** in CDCl_3_


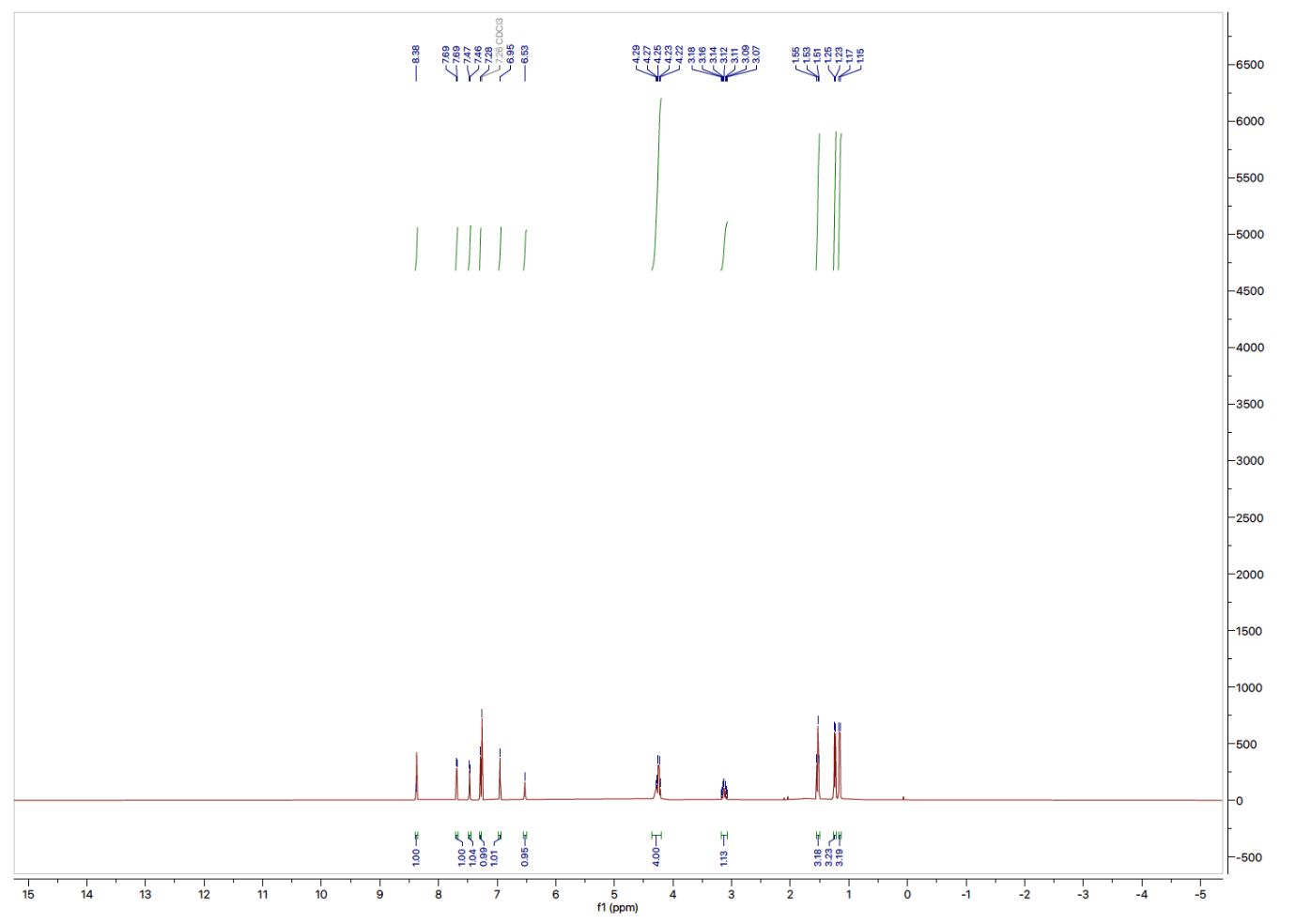


^13^C NMR of **6-amino-4-((2,4-dibromo-6-isopropylphenyl)amino)-7-ethoxyquinoline-3-carbonitrile**

**(4)** in CDCl_3_


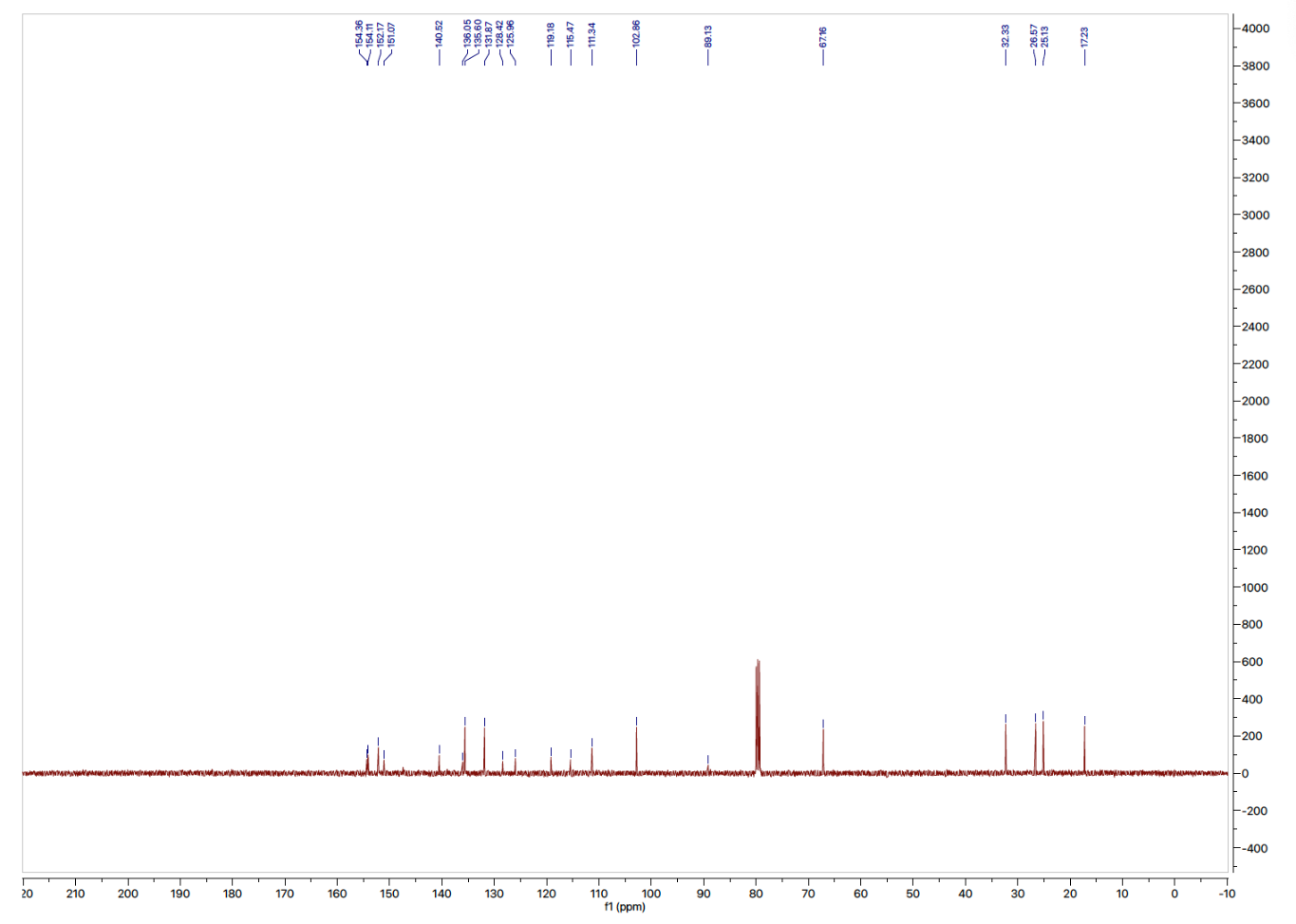


**Synthesis of 6-amino-4-((2,4-dibromo-6-isopropylphenyl)amino)-7-ethoxy-5-fluoroquinoline-3-carbonitrile (5).** 6-amino-4-((2,4-dibromo-6-isopropylphenyl)amino)-7-ethoxyquinoline-3-carbonitrile (**4**, 450 mg, 0.89 mmol, 1.0 eq) was added to a 50 mL round-bottom flask followed by selectfluor (316.3 mg, 0.89 mmol, 1.0 eq) and acetonitrile (17.8 mL, 0.05M). The reaction was allowed to stir at room temperature overnight. The reaction mixture was then diluted with ethyl acetate (30 mL), washed with water (30 mL), and brine (30 mL). The organic layer was dried with anhydrous sodium sulfate, concentrated *in vacuo*, and purified using flash silica gel column chromatography (gradient up to 30% ethyl acetate in hexanes) to give 6-amino-4-((2,4-dibromo-6-isopropylphenyl)amino)-7-ethoxy-5-fluoroquinoline-3-carbonitrile (**5**, 115.6 mg, 25% yield) as a yellow solid.

**^1^H NMR** (400 MHz, CDCl_3_) δ 8.32 (s, 1H), 7.82 (d, *J* = 27.8 Hz, 1H), 7.71 (d, *J* = 2.1 Hz, 1H), 7.48 (d, *J* = 2.1 Hz, 2H), 7.21 (s, 1H), 4.28 (q, *J* = 7.1 Hz, 2H), 4.19 (s, 2H), 3.19 (m, *J* = 6.7 Hz, 1H), 1.55 (t, *J* = 7.0 Hz, 3H), 1.28 (d, *J* = 6.9 Hz, 4H), 1.21 (d, *J* = 6.9 Hz, 4H).

**^13^C NMR** (101 MHz, CDCl_3_) δ 151.91, 151.66 (d, *J* = 11.3 Hz), 151.16, 149.10 (d, *J* = 4.9 Hz), 146.46, 144.11, 142.85, 132.87, 129.07, 126.28, 124.82 (d, *J* = 17.0 Hz), 123.87, 116.27, 105.19, 85.62, 64.97, 29.89, 23.96, 22.17, 14.52.

**^19^F NMR** (376 MHz, CDCl_3_) δ -139.99 (d, *J* = 25.2 Hz).

**MS (APCI):** Calculated for C_21_H_20_Br_2_FN_4_O [M+H]^+^:523.22 m/z; Found: 522.9975 m/z

^1^H NMR of **6-amino-4-((2,4-dibromo-6-isopropylphenyl)amino)-7-ethoxy-5-fluoroquinoline-3-carbonitrile (5)** in CDCl_3_


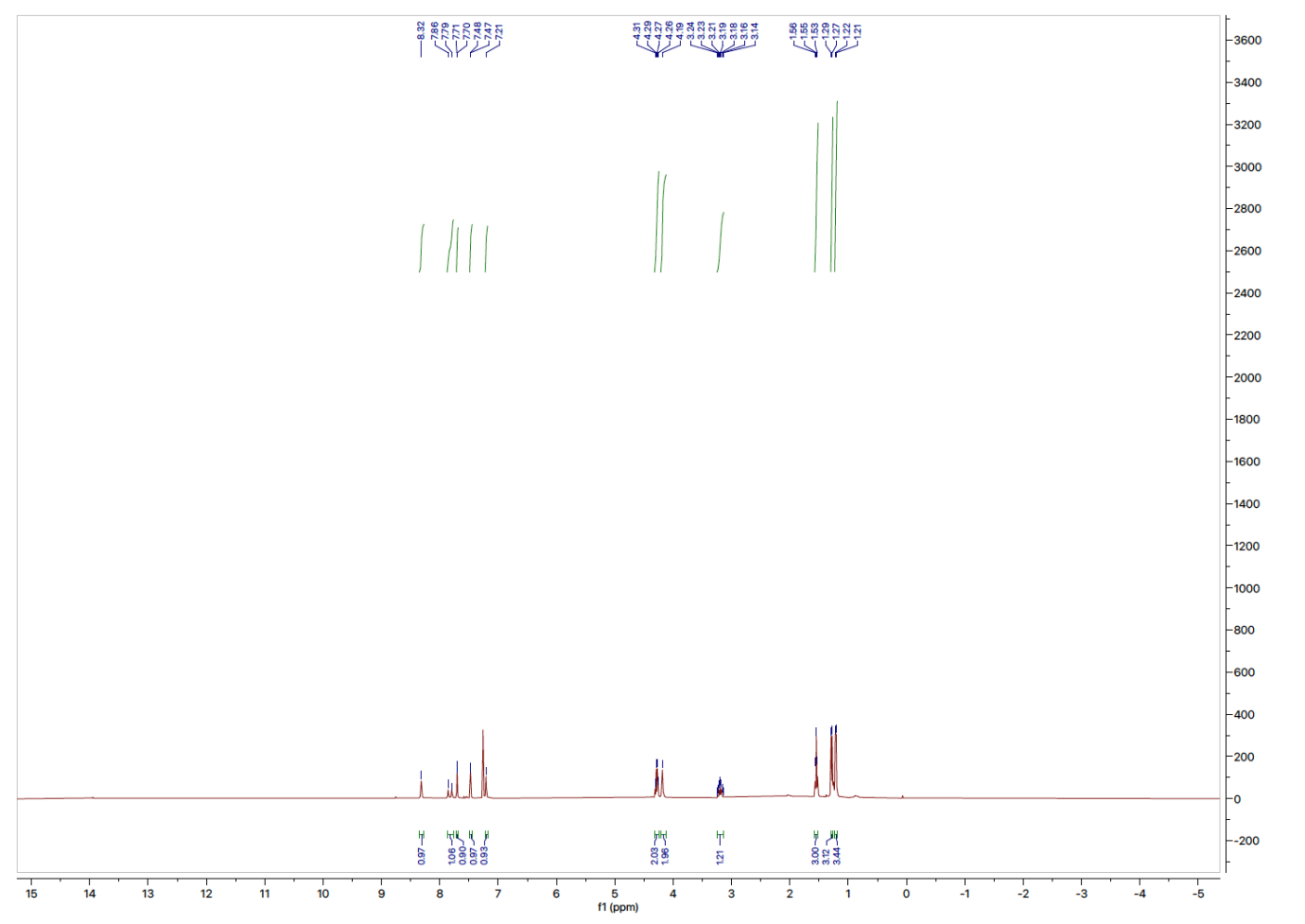


^13^C NMR of **6-amino-4-((2,4-dibromo-6-isopropylphenyl)amino)-7-ethoxy-5-fluoroquinoline-3-carbonitrile (5)** in CDCl_3_


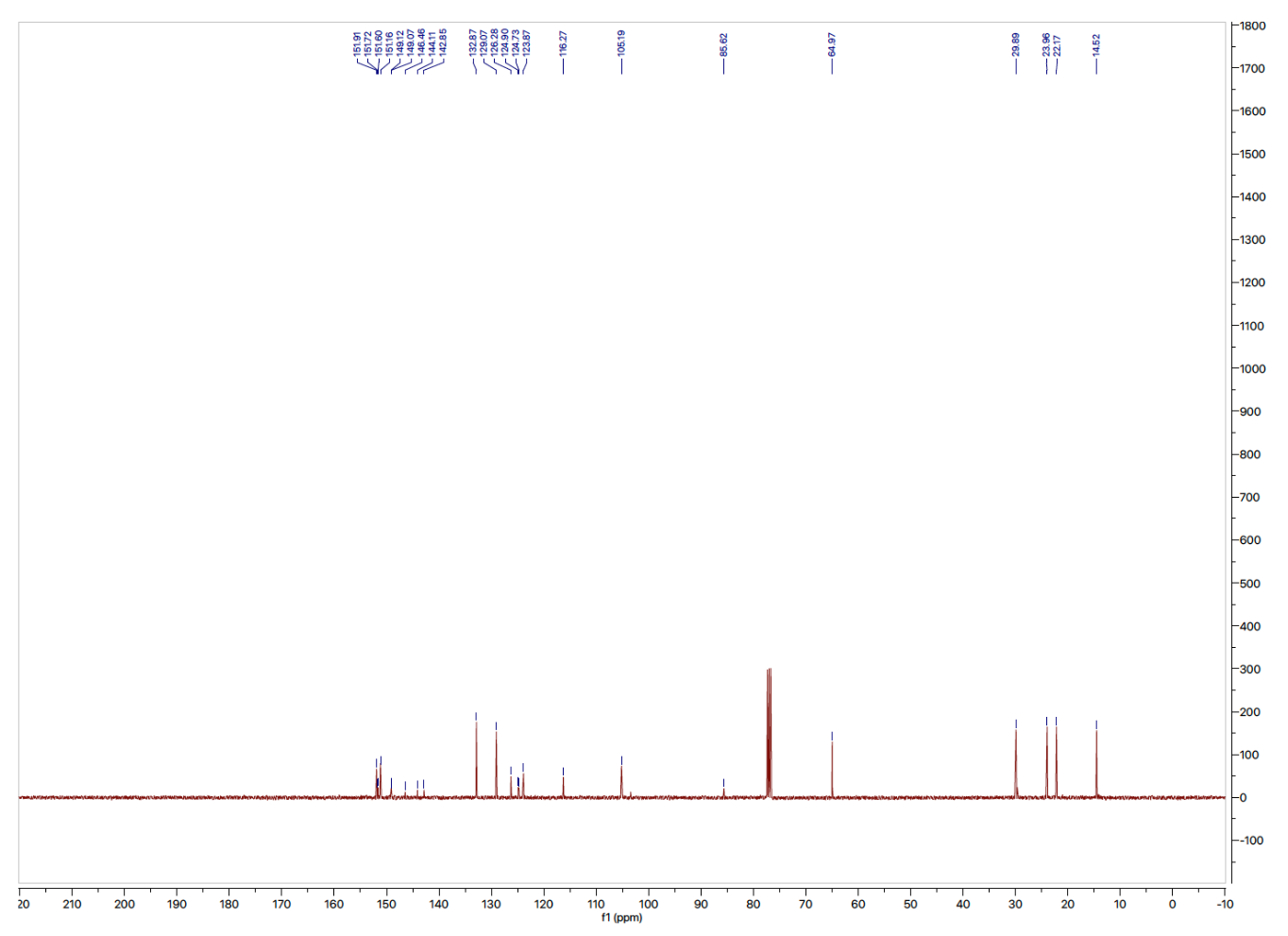


^19^F NMR of **6-amino-4-((2,4-dibromo-6-isopropylphenyl)amino)-7-ethoxy-5-fluoroquinoline-3-carbonitrile (5)** in CDCl_3_


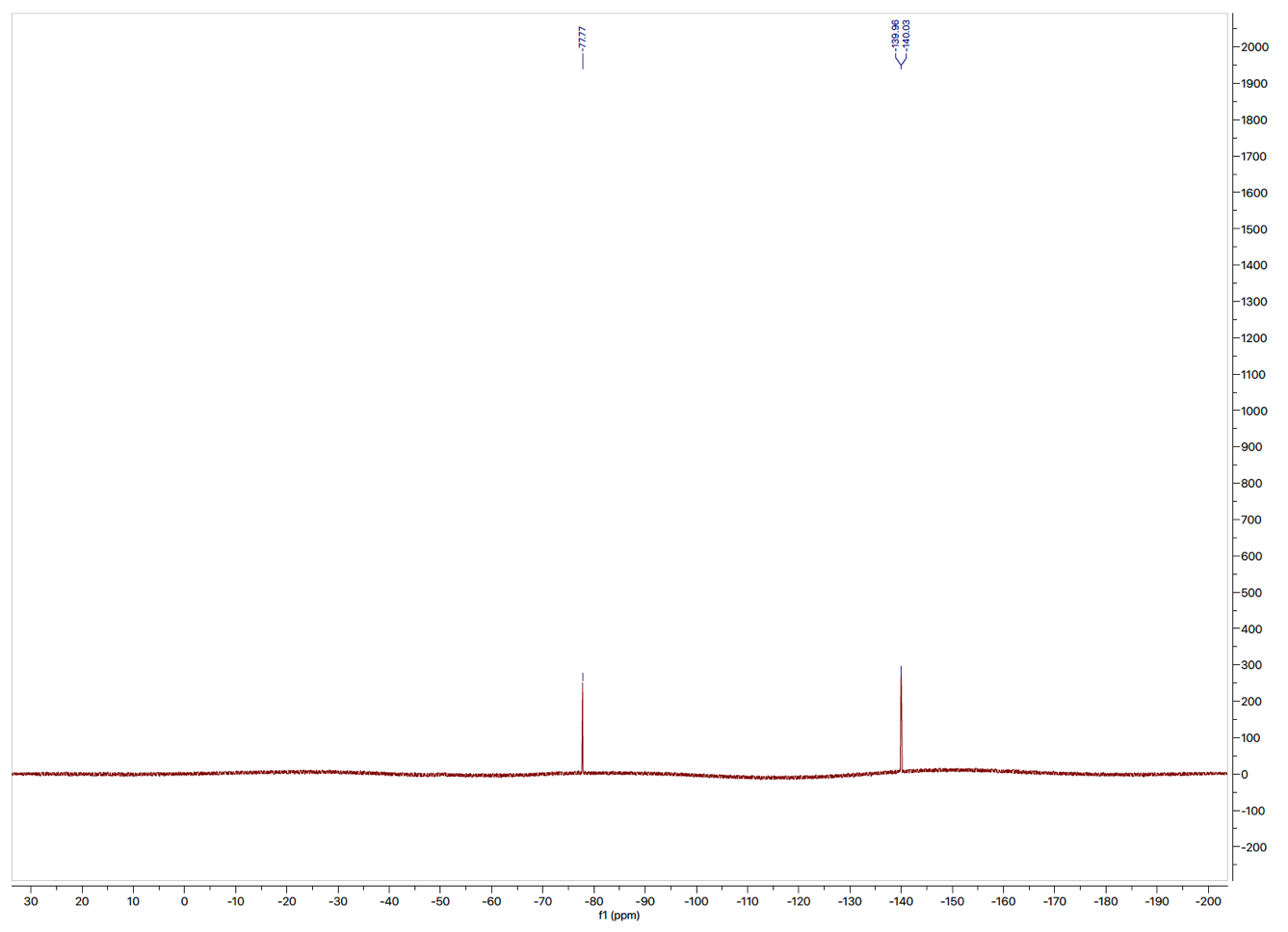


**^19^F NMR** (376 MHz, CDCl_3_)

**Synthesis of (*E*)-*N*-(3-cyano-4-((2,4-dibromo-6-isopropylphenyl)amino)-7-ethoxy-5-fluoroquinolin-6-yl)-4-(dimethylamino)but-2-enamide (BeH-1)**. First, (*E*)-4-(dimethylamino)but-2-enoic acid (43.7 mg, 0.26 mmol, 1.0 eq) was added to a 15 mL screw cap glass vial. The reaction vial was then purged with nitrogen three times, and DCM (0.4 mL, 0.66M), along with oxalyl chloride (45.2 uL, 0.53 mmol, 2.0 eq), and 1-2 drops of DMF were subsequently added at 0 °C**.** The reaction vial was allowed to warm to room temperature, and was stirred for 2 hours. The reaction mixture was then concentrated *in vacuo*, dissolved in 0.4 mL of DCM, and kept aside. In another 15 mL screw cap glass vial, **5** (138.3 mg, 0.26 mmol, 1.0 eq) was added and the reaction vial was purged with nitrogen three times. THF (0.66 mL, 0.4M) was added, and the contents were added dropwise to the crude acid chloride product at 0 °C. Afterwards, *N,N*-Diisopropylethylamine (50.4 uL, 0.29 mmol, 1.1 eq) was added dropwise and the reaction vial was allowed to warm to room temperature and was stirred overnight. The reaction mixture was then diluted with ethyl acetate (30 mL), and washed with brine (2x30 mL). The organic layer was dried with anhydrous sodium sulfate, concentrated *in vacuo*, and purified using silica gel flash column chromatography (gradient up to 10% methanol in DCM) to give (E)-N-(3-cyano-4-((2,4-dibromo-6-isopropylphenyl)amino)-7-ethoxy-5-fluoroquinolin-6-yl)-4-(dimethylamino)but-2-enamide (**6**, 41.7 mg, 25% yield) as a tan solid.

**^1^H NMR** (400 MHz, CD_3_OD) δ 8.40 (s, 1H), 7.75 (d, *J* = 2.3 Hz, 1H), 7.57 (d, *J* = 2.3 Hz, 1H), 7.25 (s, 1H), 6.97 (dt, *J* = 15.4, 6.6 Hz, 1H), 6.47 (d, *J* = 15.4 Hz, 1H), 4.30 (q, *J* = 6.9 Hz, 2H), 3.37 (d, *J* = 7.4 Hz, 2H), 3.24 (m, *J* = 6.9 Hz, 1H), 2.43 (s, 6H), 1.50 (t, *J* = 7.0 Hz, 3H), 1.27 (d, *J* = 2.5 Hz, 3H), 1.25 (d, *J* = 2.6 Hz, 3H).

**^13^C NMR** (101 MHz, CD_3_OD) δ 167.50, 160.10 (d, *J* = 7.4 Hz), 158.76, 157.25, 156.22, 155.10, 153.90 (d, *J* = 4.2 Hz), 151.62, 142.57, 136.10, 134.95, 131.40, 128.94 (d, *J* = 8.5 Hz), 126.20, 118.41, 116.37 (d, *J* = 17.3 Hz), 107.21, 87.24, 67.76, 61.96, 46.28, 32.27, 24.93, 24.29, 15.94.

**^19^F NMR** (376 MHz, CD_3_OD) δ -118.23.

**MS (APCI):** Calculated for C_21_H_20_Br_2_FN_4_O [M+H]^+^: 634.36 m/z; Found: 634.0666 m/z

^1^H NMR of **(*E*)-*N*-(3-cyano-4-((2,4-dibromo-6-isopropylphenyl)amino)-7-ethoxy-5-fluoroquinolin-6-yl)-4-(dimethylamino)but-2-enamide (BEH-1)** in CD_3_OD


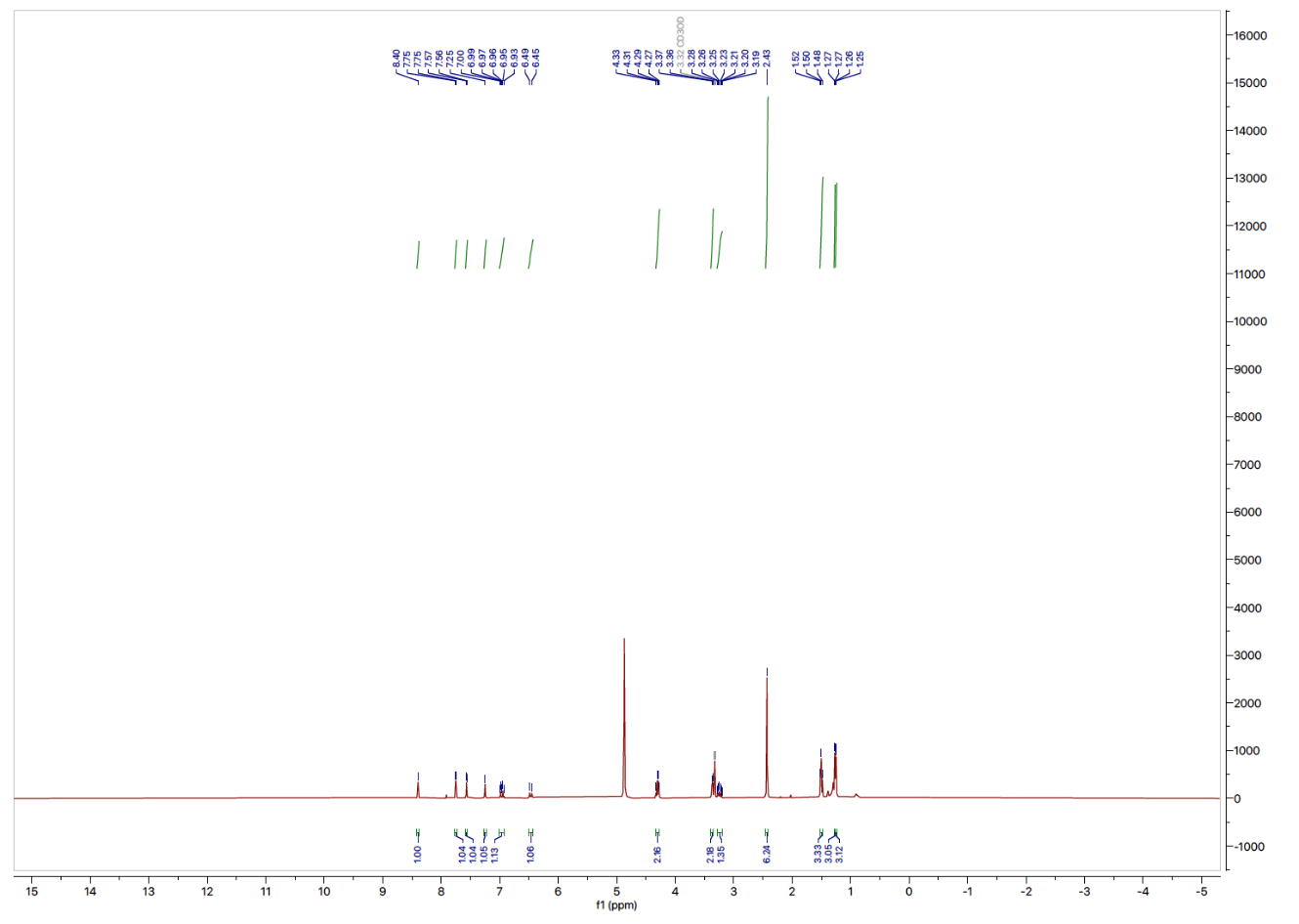


^13^C NMR of **(*E*)-*N*-(3-cyano-4-((2,4-dibromo-6-isopropylphenyl)amino)-7-ethoxy-5-fluoroquinolin-6-yl)-4-(dimethylamino)but-2-enamide (BEH-1)** in CD_3_OD

^19^F NMR of **(*E*)-*N*-(3-cyano-4-((2,4-dibromo-6-isopropylphenyl)amino)-7-ethoxy-5-fluoroquinolin-6-yl)-4-(dimethylamino)but-2-enamide (BEH-1)** in CD_3_OD

**^19^F NMR** (376 MHz, CD_3_OD)

Chiral HPLC Trace of **BEH-1**

**BEH-1** was measured with HPLC analysis using a ChiralPak ID Column in Hexanes/EtOH/DEA (70/30/0.1) with a flow rate of 1 mL/min and an injection volume of 20 uL.


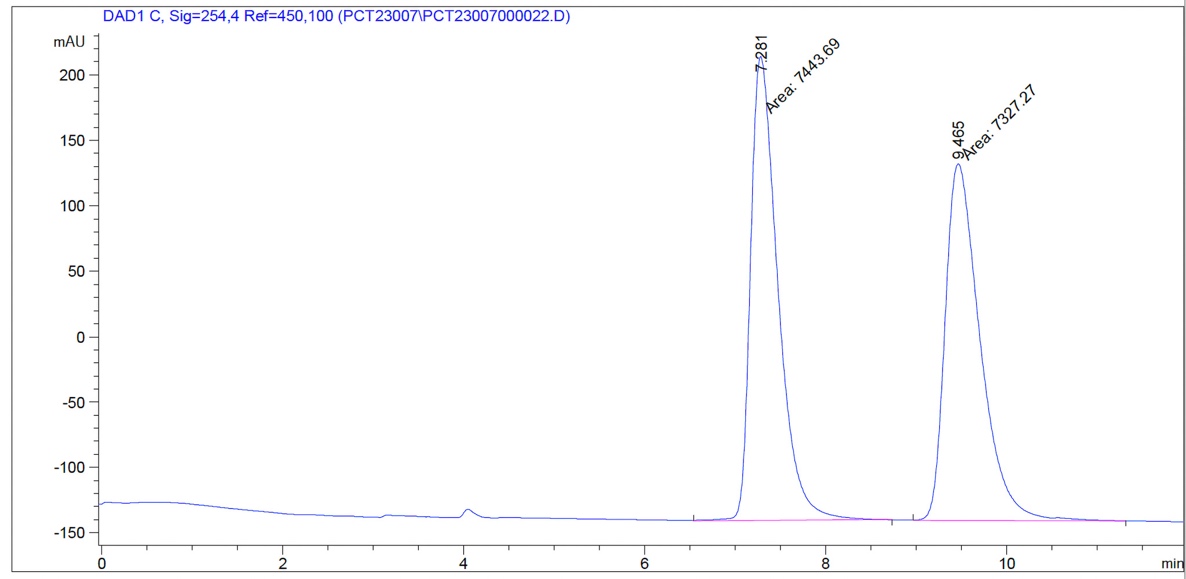

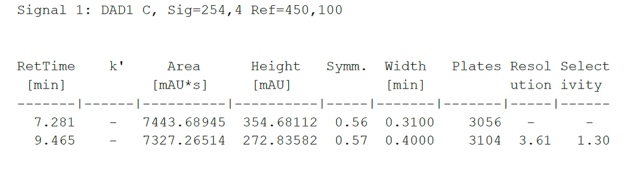

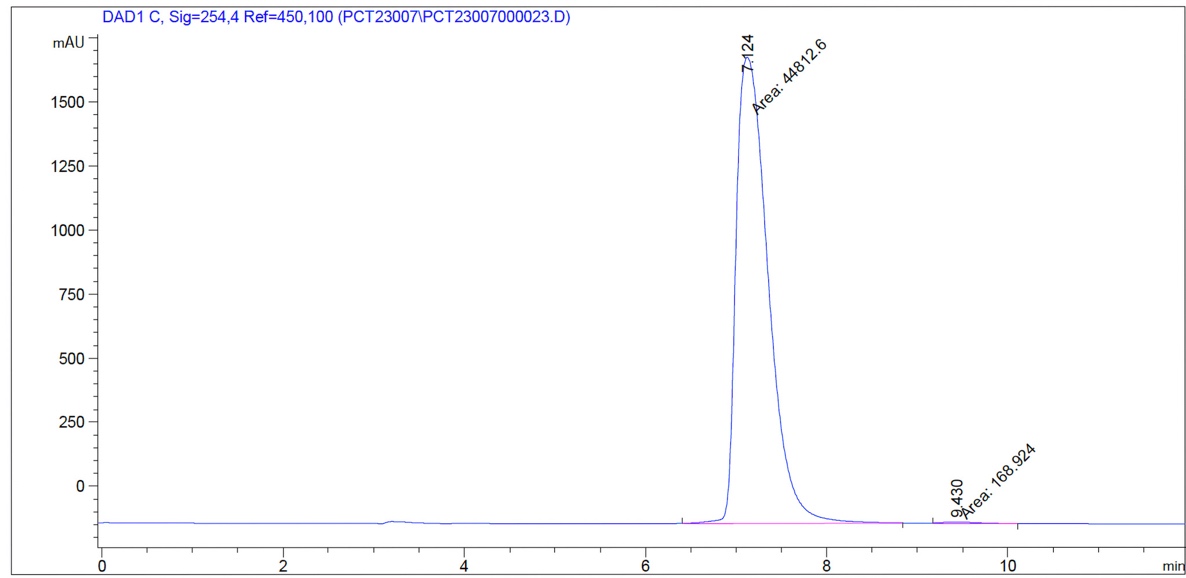

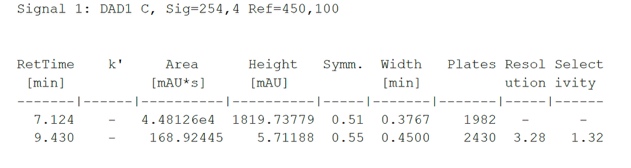

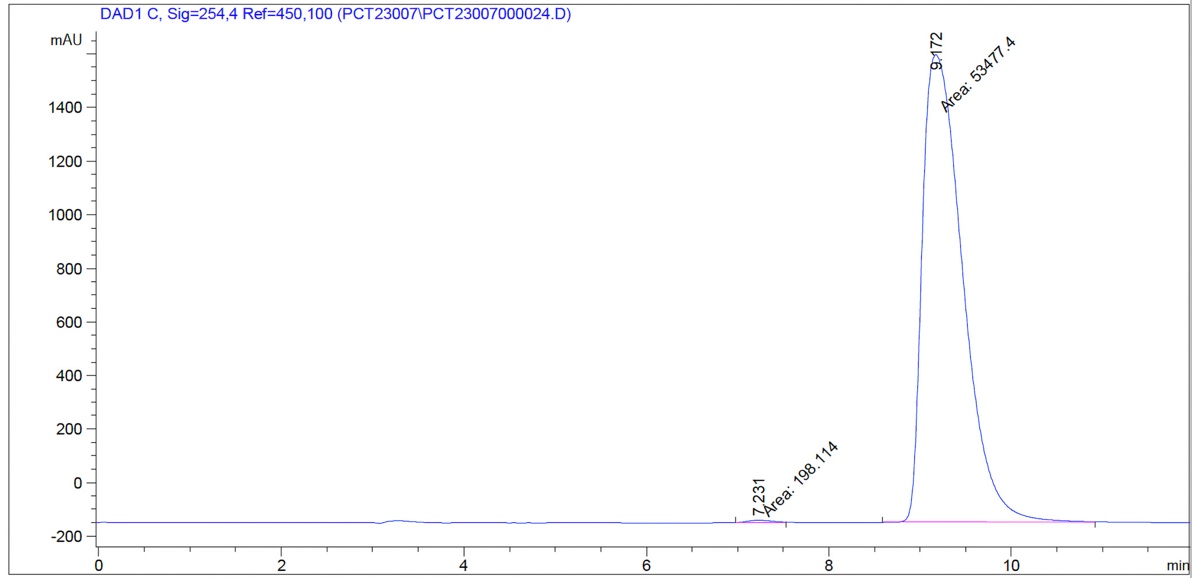

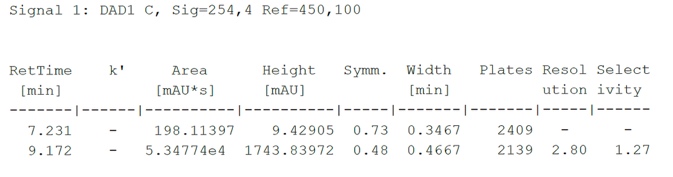


**Structures of compounds** (SO-05 to SO-253)**:**


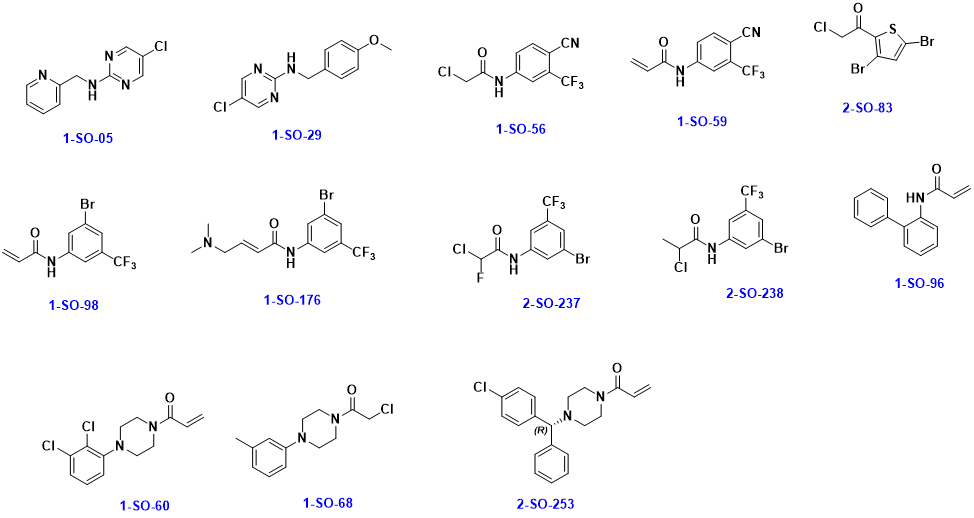


**General procedures: 1-3**

**General procedure 1:**

To a solution of the amine (1.1 equiv.) and the aldehyde (1.0 equiv.) in anhydrous MeOH (1.0 mL) was added AcOH (1.0 equiv.*)*. The reaction was stirred at 25 °C for 3 h. NaBH_3_CN (2.0 equiv*.*) was added at 0 °C. The reaction was stirred at 25 °C for another 1 h. LCMS showed the reaction was completed. The residue was poured into ice water. The aqueous phase was extracted three times with ethyl acetate. The combined organic phase was washed with brine, dried with anhydrous Na_2_SO_4_, filtered and concentrated in vacuo, and purified by silica gel flash column chromatography to give the titled compound.

**General procedure 2:**

The amine (1 equiv.) was added to a round bottom flask with a stir bar and dissolved in DCM with stirring. Triethylamine (2 equiv.) was added and the reaction was cooled to 0 ˚C. Chloroacetyl chloride (2 equiv.) was added dropwise to the flask, and the reaction was allowed to warm to room temperature as it stirred for 2 h. The reaction was washed with water, extracted with ethyl acetate, dried over Na_2_SO_4,_ and purified by silica gel flash column chromatography to give the titled compound.

**General procedure 3:**

The amine (1 equiv.) was added to a round bottom flask with a stir bar and dissolved in DCM. Triethylamine (1 equiv.) was added and the reaction was cooled to 0 ˚C. Acryloyl chloride (2 equiv.) was added dropwise to the flask, and the reaction was allowed to warm to room temperature as it stirred for 2 h. The reaction was washed with water, extracted with ethyl acetate, dried over Na_2_SO_4_, and purified silica gel flash column chromatography to give the titled compound.

**Synthesis of 5-chloro-N-(pyridin-2-ylmethyl)pyrimidin-2-amine (1-SO-05)**
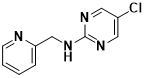


Prepared according to general procedure 1, using 5-chloropyrimidin-2-amine (266.1 mg, 2.05 mmol,1.1 equiv. ) as the amine; picolinaldehyde (200 mg,1.87 mmol, 1 equiv.) as the aldehyde. Product: off-white (356 mg, 86.4%)

**^1^H NMR (400 MHz, CDCl_3_):** δ 8.58 (s, 1H), 8.23 (s, 2H), 7.77 (s, 1H), 7.42 (d, J = 7.9 Hz, 1H), 7.33 – 7.27 (m, 1H), 6.45 (s, 1H), 4.78 (s, 2H). **^13^C NMR (101 MHz, CDCl_3_):** δ 160.11, 156.69, 156.13, 147.46, 138.00, 122.35, 119.35, 77.16, 45.88.

**LRMS** (ESI, m/z): calc’d for C_10_H_10_ClN_4_^+^ [M+H]^+^ 221.05; found 221.0

**Synthesis of 5-chloro-N-(4-methoxybenzyl)pyrimidin-2-amine (1-SO-29)**
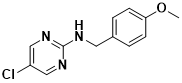


Prepared according to general procedure 1, using 5-chloropyrimidin-2-amine (266.1 mg, 2.42 mmol, 1.1 equiv.) as the amine; 4-methoxybenzaldehyde (300 mg, 2.20 mmol, 1 equiv.) as the aldehyde. Product: white powder (450 mg, 81%)

**^1^H NMR (400 MHz, CDCl_3_):** δ 8.13 (s, 2H), 7.26 (d, J = 8.7 Hz, 2H), 6.87 (d, J = 8.6 Hz, 2H), 5.76 (s, 1H), 4.51 (d, J = 5.3 Hz, 2H), 3.80 (s, 3H).**^13^C NMR (101 MHz, CDCl_3_):** δ 159.05, 156.24, 130.64, 128.95, 119.02, 114.08, 55.31, 45.33.

**LRMS** (ESI, m/z): calc’d for C_12_H_13_ClN_3_O^+^ [M+H]^+^ 250.07; found 250.0

**Synthesis of 2-chloro-N-(4-cyano-3-(trifluoromethyl)phenyl)acetamide (1-SO-56)**

Prepared according to general procedure 2, using 4-amino-2-(trifluoromethyl)benzonitrile (200 mg, 1.07 mmol, 1 equiv.) as the amine. Product: off-white solid (250 mg, 90%).
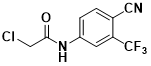


**^1^H NMR (400 MHz, CDCl_3_):** δ 8.54 (s, 1H), 8.00 (d, J = 23.5 Hz, 2H), 7.84 (d, J = 8.5 Hz, 1H), 4.24 (s, 2H).

**^13^C NMR (101 MHz, CDCl_3_)**: δ 164.53, 140.90, 135.99, 134.37, 134.05, 133.72, 123.36, 122.24, 120.64, 117.63, 115.27, 42.75.

**LRMS** (ESI, m/z): calc’d for C_10_H_5_ClF_3_N_2_O^-^ [M-H]^-^ 261.01; found 261.0

**Synthesis of N-(4-cyano-3-(trifluoromethyl)phenyl)acrylamide (1-SO-59)**
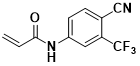


Prepared according to general procedure 3, 4-amino-2-(trifluoromethyl)benzonitrile (200 mg, 1.07 mmol, 1 equiv.) as the amine. Product: off-white solid (200 mg, 79%).

**^1^H NMR (400 MHz, Methanol-d_4_)** δ 8.30 (s, 1H), 8.04 (d, J = 10.6 Hz, 1H), 7.92 (d, J = 8.5 Hz, 1H), 6.49 – 6.39 (m, 2H), 5.86 (d, J = 11.8 Hz, 1H).

**^13^C NMR (101 MHz, Methanol-d_4_)** δ 166.65, 145.02, 137.45, 134.77, 134.55, 134.36, 131.94, 129.87, 123.65, 118.57, 118.52, 104.75, 49.00.

**LRMS** (ESI, m/z): calc’d for C_11_H_6_F_3_N_2_O^-^ [M-H]^-^ 239.05; found 239.0

**Synthesis of 2-chloro-1-(3,5-dibromothiophen-2-yl)ethan-1-one(2-SO-83)**
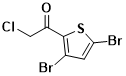


To a solution consisting of 1 g (4.133 mmol, 1.0 equiv.) 2,4-dibromo-thiophene and 560 mg (4.96 mmol, 1.2 equiv.) chloroacetyl chloride in 12 mL of absolute dichloromethane, 1.10 g (8.26 mmol, 2 equiv.) anhydrous aluminum chloride was added portionwise, that was not exceeded 15 oC (ice bath) and stirred for 3 hrs at this temperature. When deemed complete via TLC, 2 N hydrochloric acid (10mL) was added, and stirred for 15 min until the complete destruction of the complex, the phases were separated and the aqueous phase was extracted twice with dichloromethane. The collected organic phases were washed successively with saturated sodium bicarbonate solution and water, dried with sodium sulfate, treated with activated carbon, filtered, and evaporated to yield the yellow solid product 1.2g (91 %).

**^1^H NMR (400 MHz, CDCl_3_) :** δ 7.12 (s, 1H), 4.77 (s, 2H).

**^13^C NMR (101 MHz, CDCl_3_):** δ 182.65, 138.80, 135.83, 123.71, 114.30, 47.67.

**Synthesis of N-(3-bromo-5-(trifluoromethyl)phenyl)acrylamide (1-SO-98)**
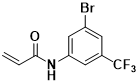


Prepared according to general procedure 3, 3-bromo-5-(trifluoromethyl)aniline (300 mg, 1.25 mmol, 1 equiv.) as the amine. Product: off-white solid (320 mg, 86%)

**^1^H NMR (400 MHz, CDCl_3_):** 1H NMR (400 MHz, Chloroform-d) δ 8.05 (s, 1H), 7.77 (s, 1H), 7.50 (s, 1H), 6.55 – 6.41 (m, 1H), 6.26 (dd, J = 16.9, 10.3 Hz, 1H), 5.85 (dd, J = 10.2, 1.0 Hz, 1H).

**^13^C NMR (101 MHz, CDCl_3_):** δ 163.91, 139.39, 132.99, 132.66, 132.33, 125.95, 124.25, 123.10, 121.52, 115.32.

**LRMS** (ESI, m/z): calc’d for C_10_H_6_BrF_3_NO^-^ [M-H]^-^ 291.97; found 291.9

**Synthesis of (E)-N-(3-bromo-5-(trifluoromethyl)phenyl)-4-(dimethylamino)but-2-enamide (1-SO-176)**
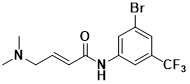


To a solution of (E)-4-(dimethylamino)but-2-enoic acid (194 mg, 1.5 mmol, 1.2 equiv.) in dichloromethane (CH_2_Cl_2_) (5 mL) at 0 °C, stirring under nitrogen, was added oxalyl chloride (317 μL, 2.50 mmol, 2 equiv.) via syringe followed by a catalytic amount of DMF (15 μL). The resulting mixture was warmed to room temperature and stirred for 4 h. Next, the reaction mixture was concentrated in vacuo to afford the crude (E)-4-(dimethylamino)but-2-enoyl chloride, which was used directly in the next step without further purification. To a stirring solution of 3-bromo-5-(trifluoromethyl)aniline (300 μL, 1.25 mmol, 1 equiv.) and Et_3_N (253 μL, 2.5 mmol, 2 equiv.) in CH_2_Cl_2_ (5 mL) at 0 °C, was slowly added a cold solution of the crude (E)-4-(dimethylamino)but-2-enoyl chloride in CH_2_Cl_2_ (5 mL). The reaction mixture was continually stirred at 25 °C, under nitrogen, for another 18 h. LCMS showed the reaction was completed. Next, the reaction was quenched with water. The aqueous phase was extracted three times with dichloromethane. The combined organic phase was washed with brine, dried with anhydrous Na_2_SO4, filtered and concentrated in vacuo, and purified by silica gel flash column chromatography (7% methanol in DCM) to give (272 mg, 62%) of (E)-N-(3-bromo-5-(trifluoromethyl)phenyl)-4-(dimethylamino)but-2-enamide.

**^1^H NMR (400 MHz, CDCl_3_) :** δ 8.70 (s, 1H), 7.99 (s, 1H), 7.77 (s, 1H), 7.42 (s, 1H), 7.06 – 6.90 (m, 1H), 6.19 (d, J = 15.3 Hz, 1H), 3.07 (d, J = 5.8 Hz, 2H), 2.23 (s, 6H).

**^13^C NMR (101 MHz, CDCl_3_) :** δ 164.81, 144.42, 140.24, 133.52, 133.19, 132.86, 132.53, 127.38, 126.47, 125.49, 124.66, 124.31, 123.33, 121.95, 115.95, 77.16, 60.63, 45.79.

**LRMS** (ESI, m/z): calc’d for C_13_H_13_BrF_3_N_2_O^+^ [M-H]^-^ 349.02; found 349.0.
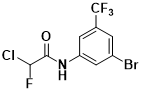


**Synthesis of N-(3-bromo-5-(trifluoromethyl)phenyl)-2-chloro-2-fluoroacetamide (2-SO-237)**

To a stirred solution of sodium chlorofluoroacetate (336 mg, 2.50 mmol, 2 equiv.), HOBt•H_2_O (338 mg, 2.50 mmol, 2 equiv.), and propylphosphonic anhydride (T3P) (50 wt. % in CH_2_Cl_2_, 1.15 g, 2.04 mmol, 2 equiv.) in dry DMF (3.0 mL) was added DIPEA (323 μL, 2.50 mmol, 2 equiv.) and 3-bromo-5-(trifluoromethyl)aniline (300 mg, 1.25 mmol, 1 equiv.) at 0 °C. After stirring overnight at ambient temperature, the reaction mixture was diluted with CH_2_Cl_2_ and saturated sodium bicarbonate. The organic layer was separated and the aqueous phase was extracted with CH_2_Cl_2_. The combined organic layers were dried over anhydrous magnesium sulfate, filtered, and concentrated *in vacuo*. The resultant crude was purified by flash column chromatography on silica gel using 7% methanol/CH_2_Cl_2_ to give the target compound (260 mg, 63% yield) as pale-yellowish oil.

**^1^H NMR (400 MHz, CDCl_3_) :** δ 8.05 (s, 1H), 7.79 (s, 1H), 7.60 (s, 1H), 6.44 (d, J = 50.9 Hz, 1H).

**^13^C NMR (101 MHz, CDCl_3_) :** δ 162.12, 161.90, 137.63, 133.66, 132.99, 126.20, 125.49, 123.38, 115.68, 95.23, 92.67.

**LRMS** (ESI, m/z): calc’d for C_9_H_6_BrClF_4_NO^+^ [M+H]^+^ 333.92; found 333.9

**Synthesis of N-(3-bromo-5-(trifluoromethyl)phenyl)-2-chloropropanamide (2-SO-238)**

To a stirred solution of 3-bromo-5-(trifluoromethyl)aniline (500 µL, 2.08 mmol) in DCM (5ml), triethylamine (232 µL, 2.29 mmol, 1.1 equiv.), 2-chloropropanoyl chloride (317 mg, 2.5 mmol, 1.2 equiv.) at 0°C was added dropwise and maintained with an ice bath. The reaction mixture was stirred at room temperature for an additional four hours. The reaction was washed with water, extracted with ethyl acetate, dried over Na2SO4, and purified by silica gel flash column chromatography (30% ethyl acetate in hexanes) to give the titled compound (560 mg, 81%).
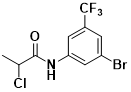


**^1^H NMR (400 MHz, CDCl_3_) :** δ 8.39 (s, 1H), 8.02 (s, 1H), 7.77 (s, 1H), 7.55 (s, 1H), 4.56 (q, J = 7.1 Hz, 1H), 1.84 (s, 3H).

**^13^C NMR (101 MHz, CDCl_3_) :** δ 168.21, 139.11, 133.91, 133.57, 133.24, 132.91, 126.32, 125.24, 124.63, 123.63, 115.85, 77.16, 56.39, 22.95.

**LRMS** (ESI, m/z): calcd for C_10_H_9_BrClF_3_NO^+^ [M+H]^+^ 329.94; found 329.9
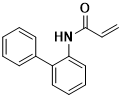


**Synthesis of N-([1,1'-biphenyl]-2-yl)acrylamide (1-SO-96)**

Prepared according to general procedure 3, [1,1'-biphenyl]-2-amine (250 mg, 1.48 mmol, 1 equiv.) as the amine. Product: white solid (240 mg, 74%)

**^1^H NMR (400 MHz, CDCl_3_) :** δ 8.44 (s, 1H), 7.49 (dd, J = 8.0, 6.5 Hz, 2H), 7.46 – 7.35 (m, 4H), 7.35 – 7.25 (m, 2H), 7.19 (t, J = 7.9 Hz, 1H), 6.27 (d, J = 17.6 Hz, 1H), 6.00 (d, J = 22.9 Hz, 1H), 5.66 (d, J = 11.4 Hz, 1H).

**^13^C NMR (101 MHz, CDCl_3_)** : δ 138.42, 135.02, 131.85, 130.51, 129.74, 128.97, 128.54, 127.79, 124.91, 121.79, 77.16.

**LRMS** (ESI, m/z): calcd for C_16_H_13_NO^+^ [M+H]^+^ 224.10; found 224.1

**Synthesis of 1-(4-(2,3-dichlorophenyl)piperazin-1-yl)prop-2-en-1-one (1-SO-60)**
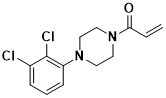


Prepared according to general procedure 2, 1-(2,3-dichlorophenyl)piperazine (200 mg, 0.865 mmol, 1 equiv.) as the amine. Product: off-white solid (151 mg, 61%)

**^1^H NMR (400 MHz, CDCl_3_) :** 5.73 (d, J = 12.5 Hz, 1H), 3.81 (d, J = 53.6 Hz, 4H), 3.03 (d, J = 4.9 Hz, 4H).

**^13^C NMR (101 MHz, CDCl_3_) :** δ 165.58, 150.56, 134.24, 128.17, 127.80, 127.59, 127.41, 125.28, 118.82, 51.80, 51.06, 46.07, 42.16.

**LRMS** (ESI, m/z): calcd for C_13_H_15_Cl_2_N_2_O^+^ [M+H]^+^ 285.05; found 285.0

**Synthesis of 2-chloro-1-(4-(m-tolyl)piperazin-1-yl)ethan-1-one (SO-68)**

Prepared according to general procedure 2, using 1-(m-tolyl)piperazine (200 mg, 1.13 mmol, 1 equiv.) as the amine. Product: off-white solid (270 mg, 94%)
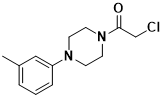


**^1^H NMR (400 MHz, CDCl_3_) :** δ 7.18 (t, J = 7.8 Hz, 1H), 6.81 – 6.71 (m, 3H), 4.11 (s, 2H), 3.83 – 3.63 (m, 4H), 3.20 (dt, J = 20.1, 5.2 Hz, 4H), 2.33 (s, 3H).

**^13^C NMR (101 MHz, CDCl_3_) :** δ 165.14, 150.80, 139.09, 129.15, 121.78, 117.71, 113.96, 49.83, 49.46, 46.28, 42.14, 40.84, 21.74.

**LRMS** (ESI, m/z): calcd for C_13_H_16_ClN_2_O^-^ [M-H]^-^ 252.10; found 252.1

**Synthesis of (R)-1-(4-((4-chlorophenyl)(phenyl)methyl)piperazin-1-yl)prop-2-en-1-one (SO-253)**
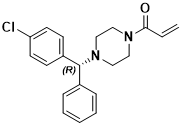


Prepared according to general procedure 3, (R)-1-((4-chlorophenyl)(phenyl)methyl)piperazine (200 mg, 0.697 mmol, 1 equiv.) as the amine. Product: pale-yellowish solid (164 mg, 69%).

**^1^H NMR (400 MHz, CDCl_3_) :** δ 7.38 – 7.33 (m, 4H), 7.29 (d, J = 7.3 Hz, 2H), 7.26 – 7.19 (m, 3H), 6.51 (dd, J = 16.8, 10.5 Hz, 1H), 6.26 (dd, J = 16.8, 1.9 Hz, 1H), 5.66 (dd, J = 10.5, 1.8 Hz, 1H), 4.22 (s, 1H), 3.61 (d, J = 54.6 Hz, 4H), 2.37– 2.38 (m, 4H).

**^13^C NMR (101 MHz, CDCl_3_) :** δ 165.32, 141.53, 140.74, 132.88, 129.14, 128.85, 128.78, 127.84, 127.80, 127.45, 127.41, 75.21, 52.06, 51.47, 45.91, 42.09.

**LRMS** (ESI, m/z): calcd for C_20_H_22_ClN_2_O^-^ [M-H]^-^ 341.13; found 341.1

**
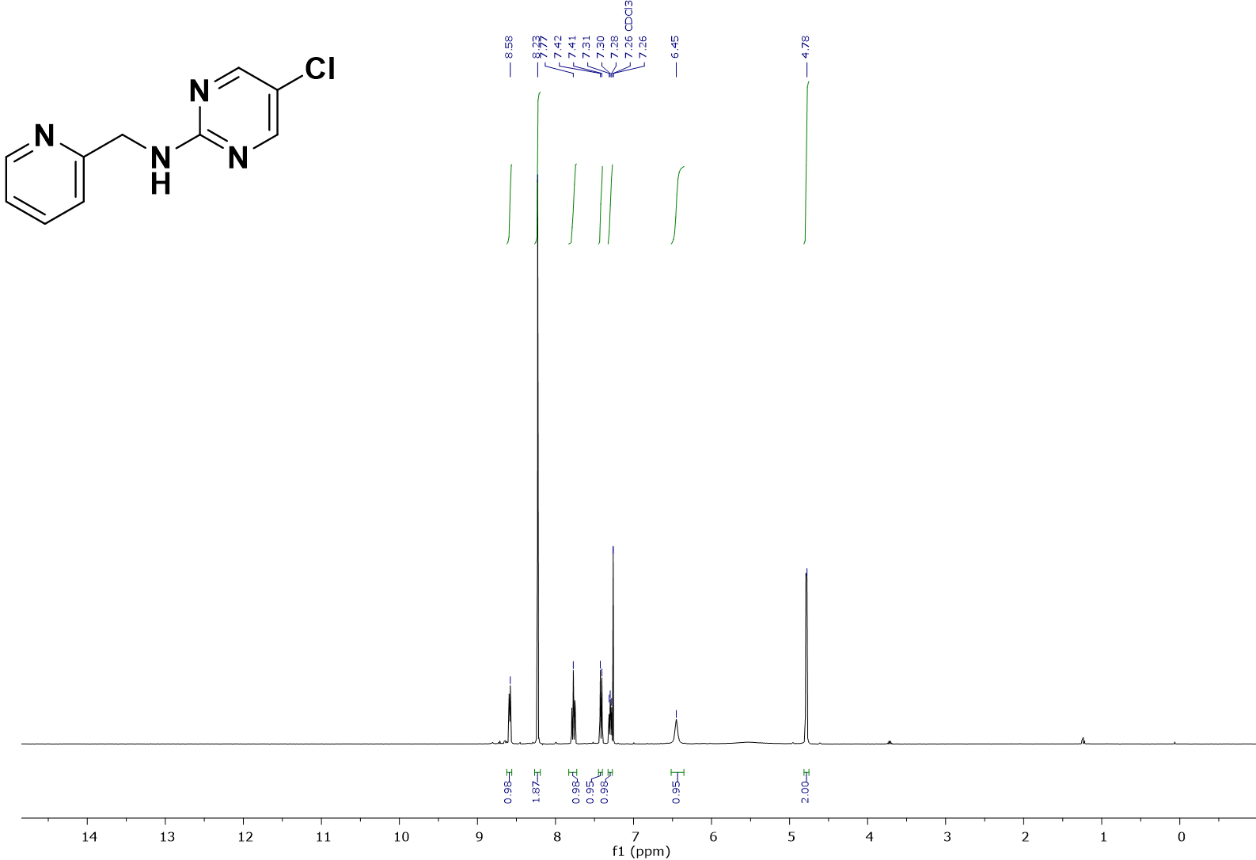
**

^1^H NMR of **5-chloro-N-(pyridin-2-ylmethyl)pyrimidin-2-amine (1-SO-05)** in CDCl_3_


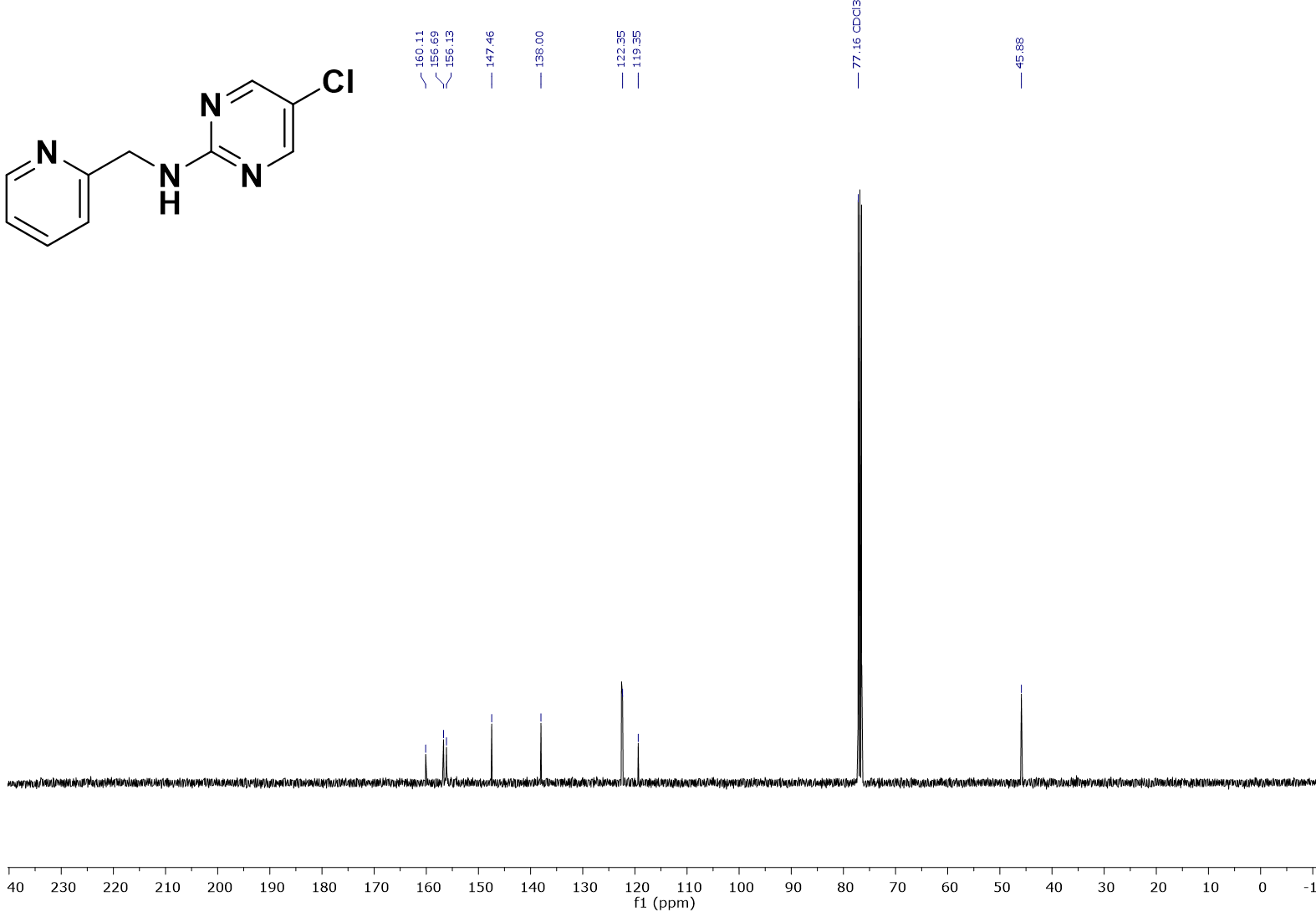


^13^C NMR of **5-chloro-N-(pyridin-2-ylmethyl)pyrimidin-2-amine (1–SO-05)** in CDCl_3_

**
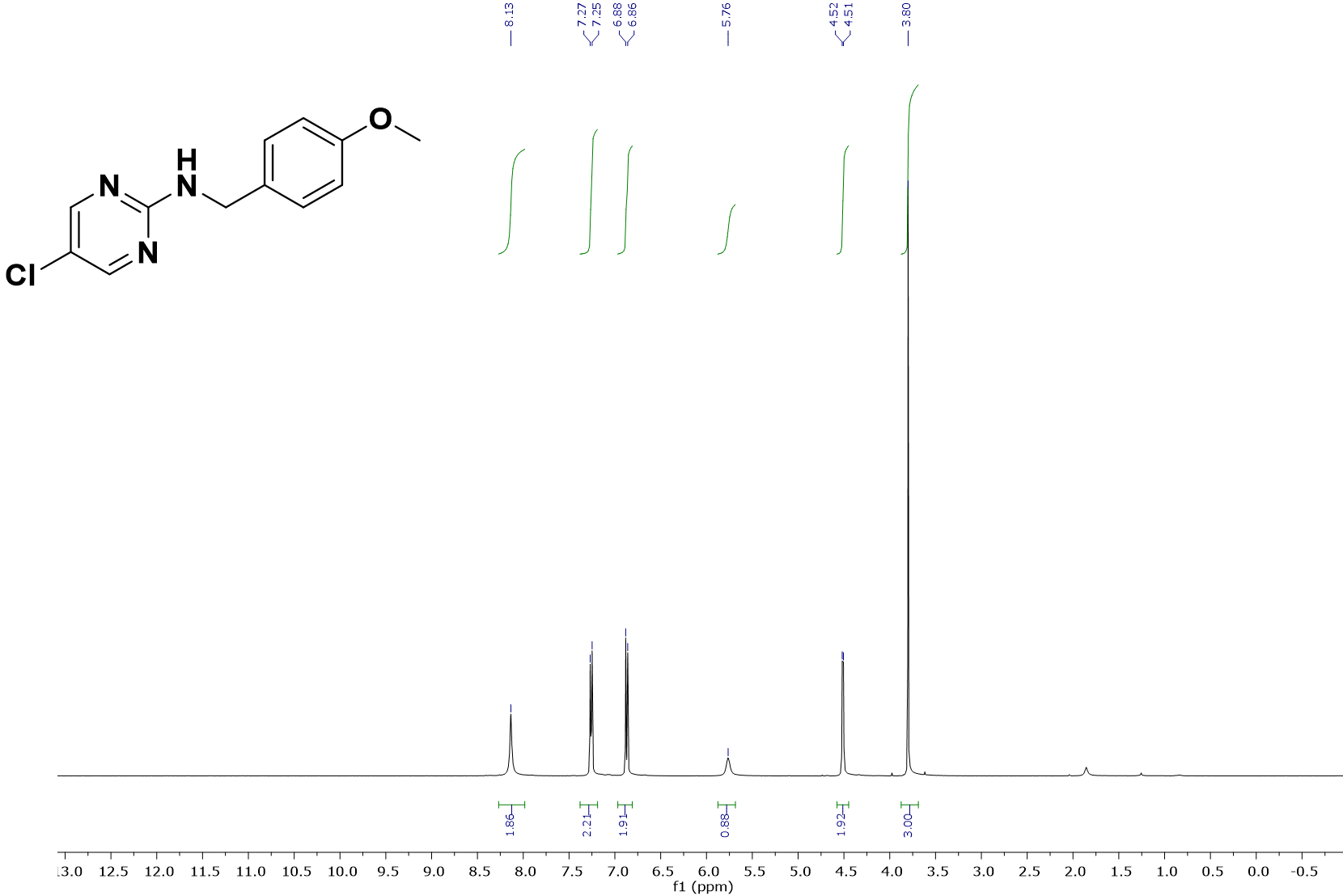
**

^1^H NMR of **Synthesis of 5-chloro-N-(4-methoxybenzyl)pyrimidin-2-amine (1-SO-29)** in CDCl_3_


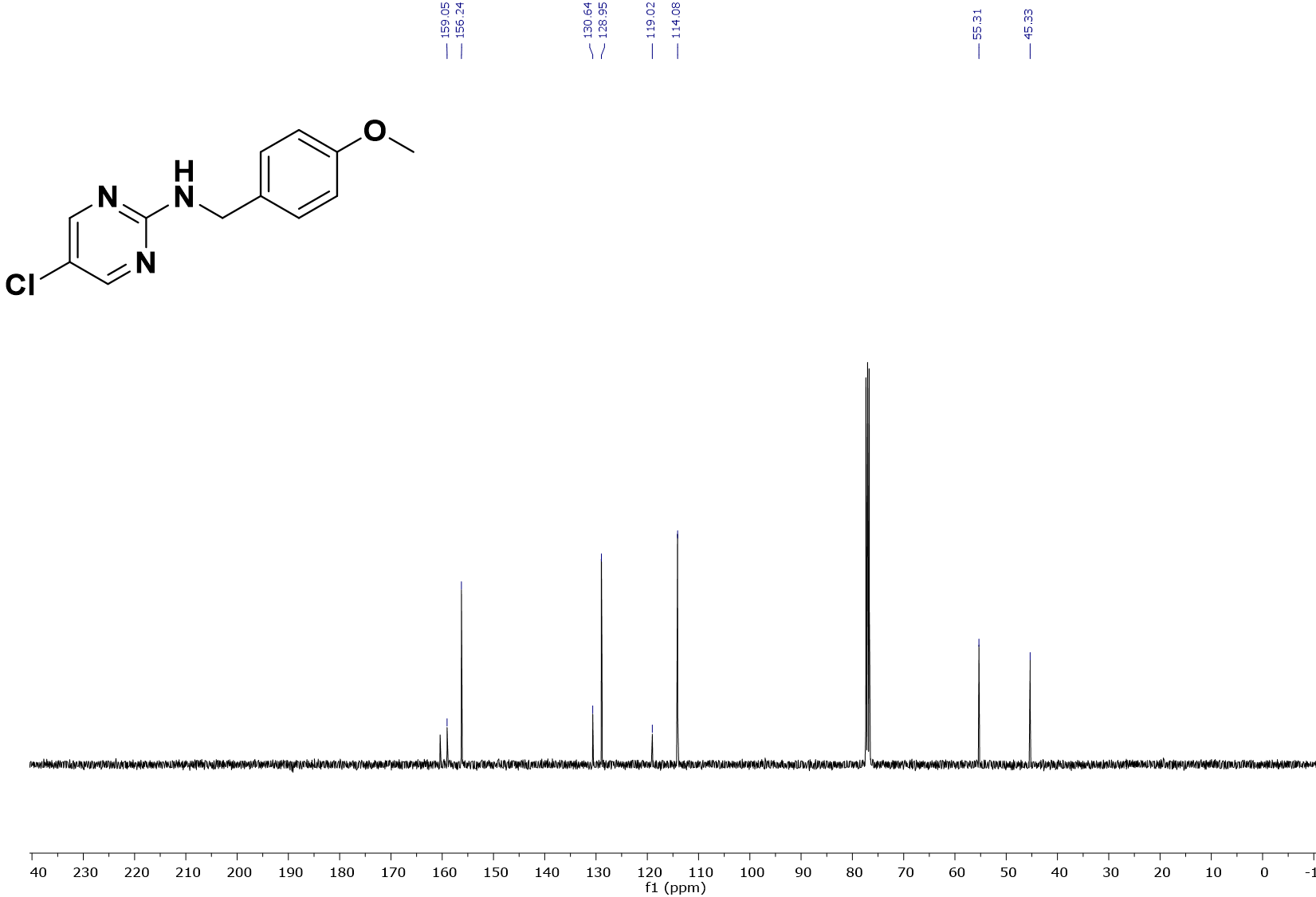


^13^C NMR of **Synthesis of 5-chloro-N-(4-methoxybenzyl)pyrimidin-2-amine (1-SO-29)** in CDCl_3_


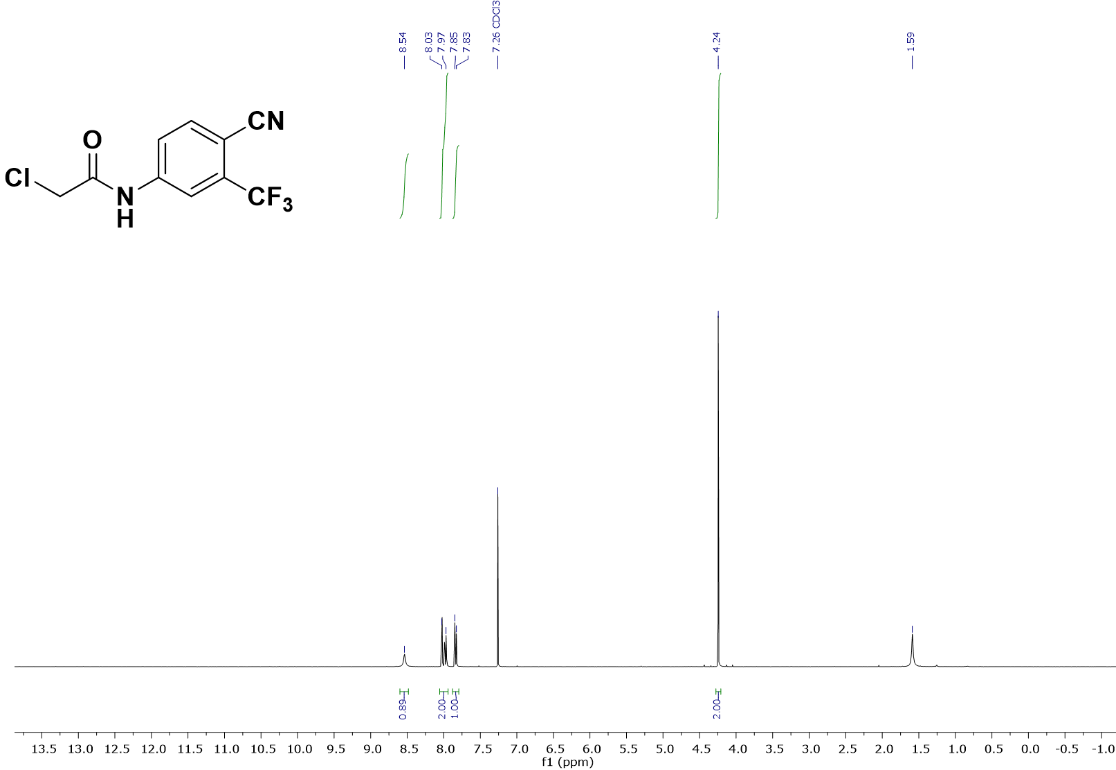


^1^H NMR of **2-chloro-N-(4-cyano-3-(trifluoromethyl)phenyl)acetamide (1-SO-56)** in CDCl_3_


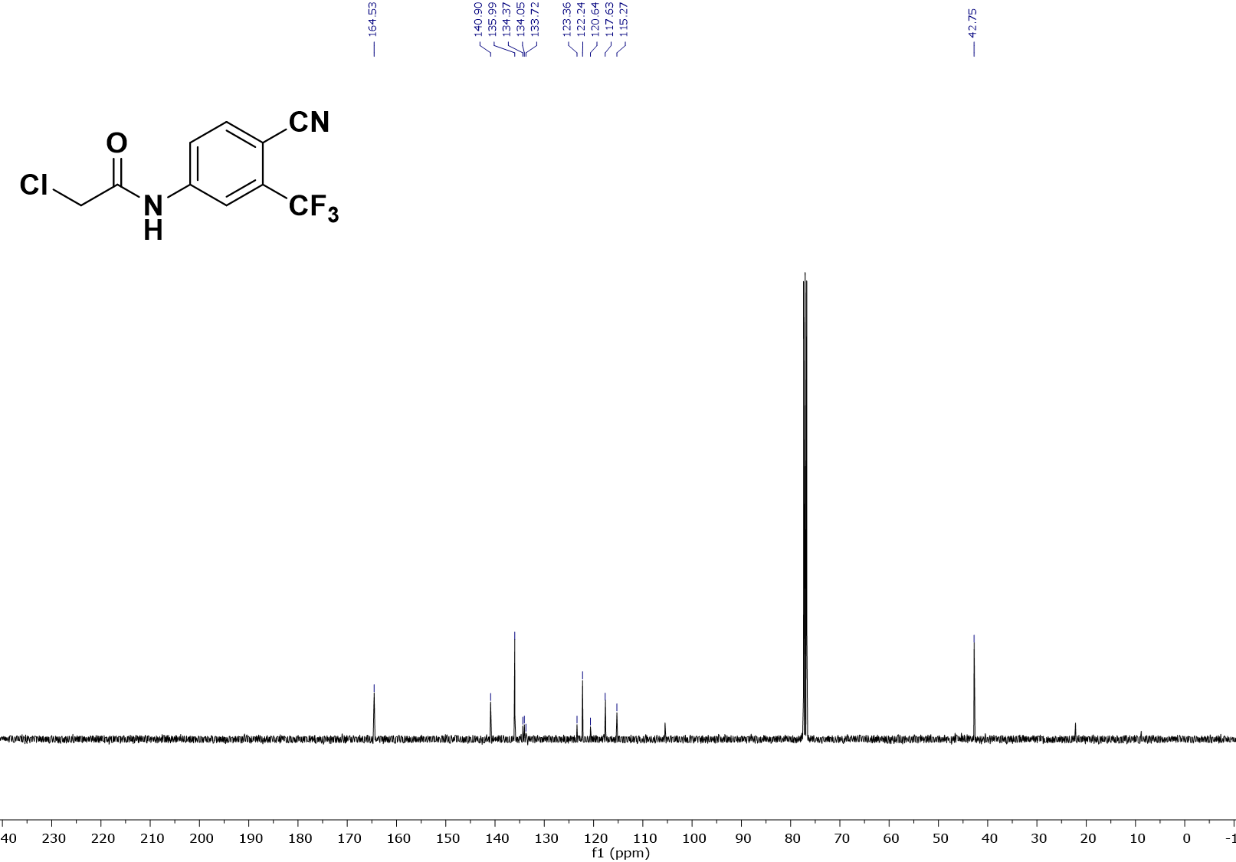


^13^C NMR of **2-chloro-N-(4-cyano-3-(trifluoromethyl)phenyl)acetamide (1-SO-56)** in CDCl_3_


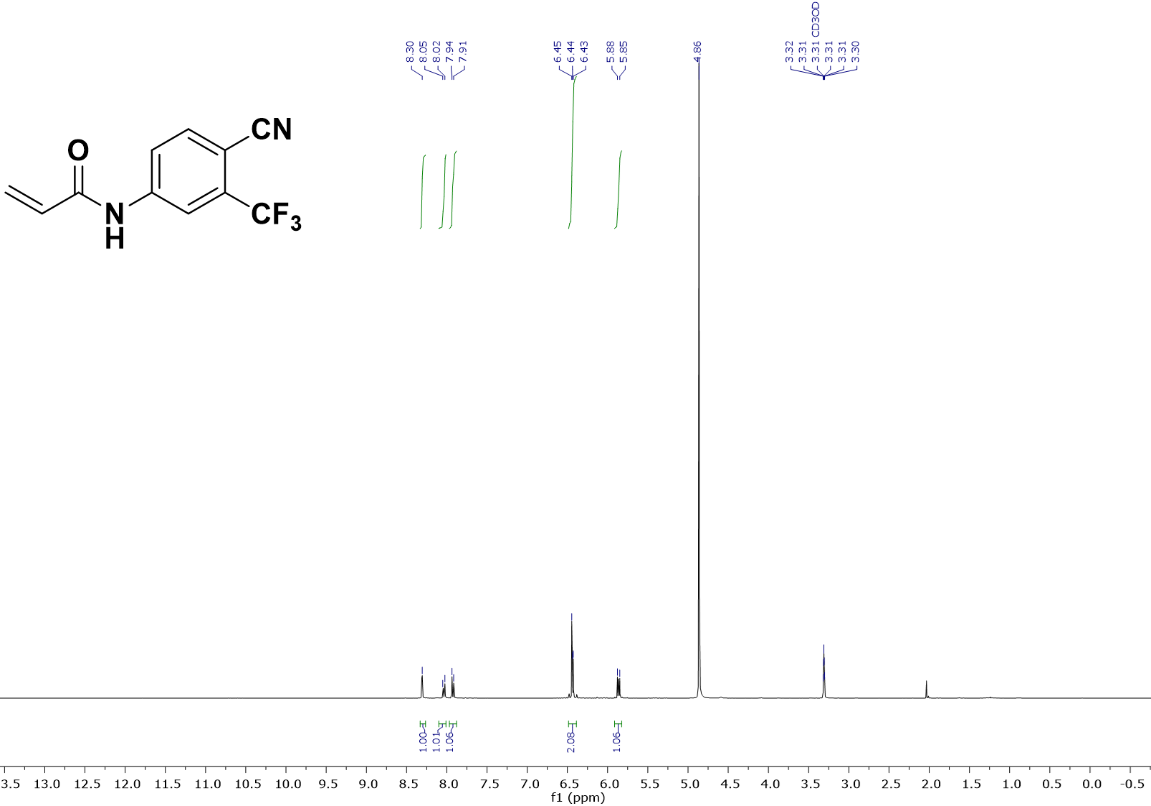


^1^H NMR of **N-(4-cyano-3-(trifluoromethyl)phenyl)acrylamide (1-SO-59)** in CD_3_OD


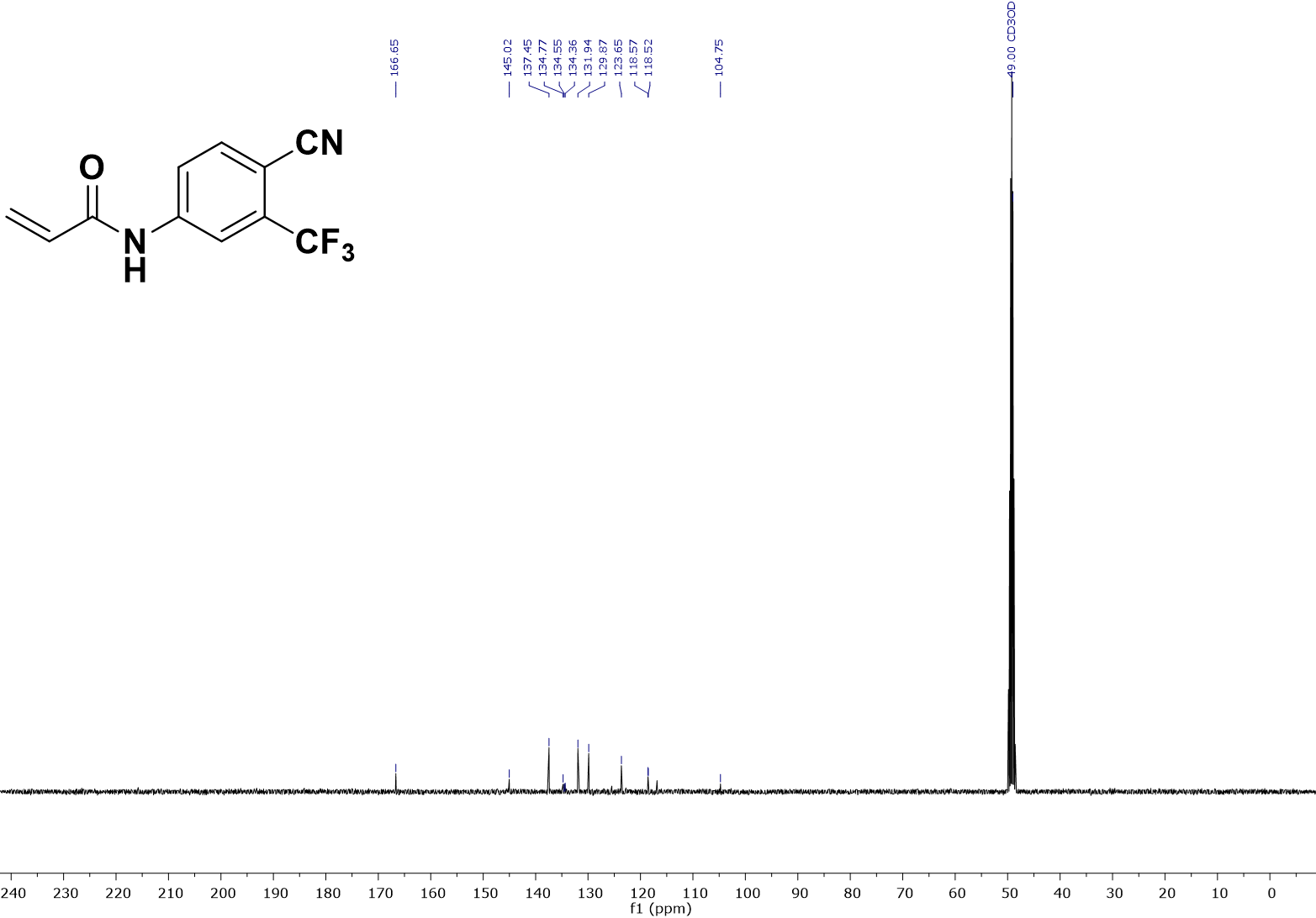


^13^C NMR of **N-(4-cyano-3-(trifluoromethyl)phenyl)acrylamide (1-SO-59)** in CD_3_OD


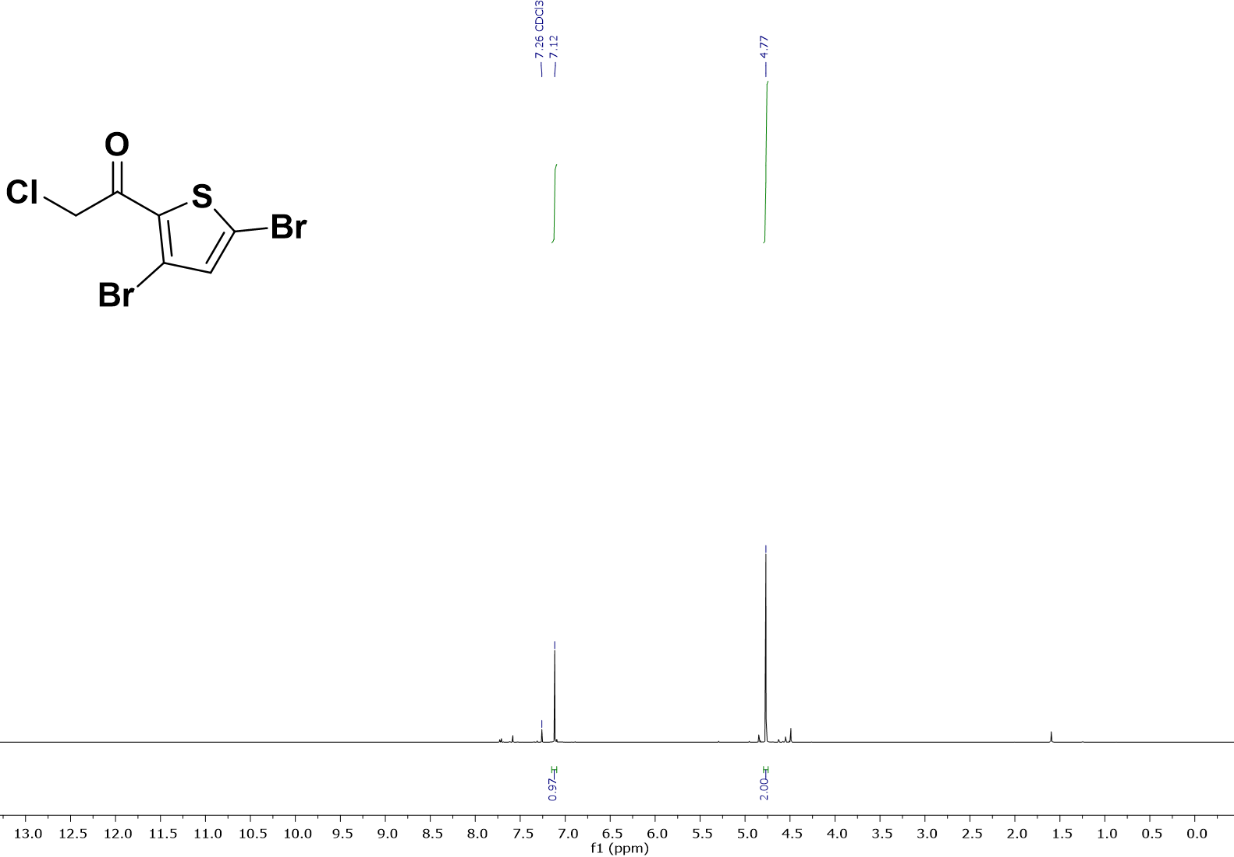


^1^H NMR of **2-chloro-1-(3,5-dibromothiophen-2-yl)ethan-1-one (1-SO-83)** in CDCl_3_


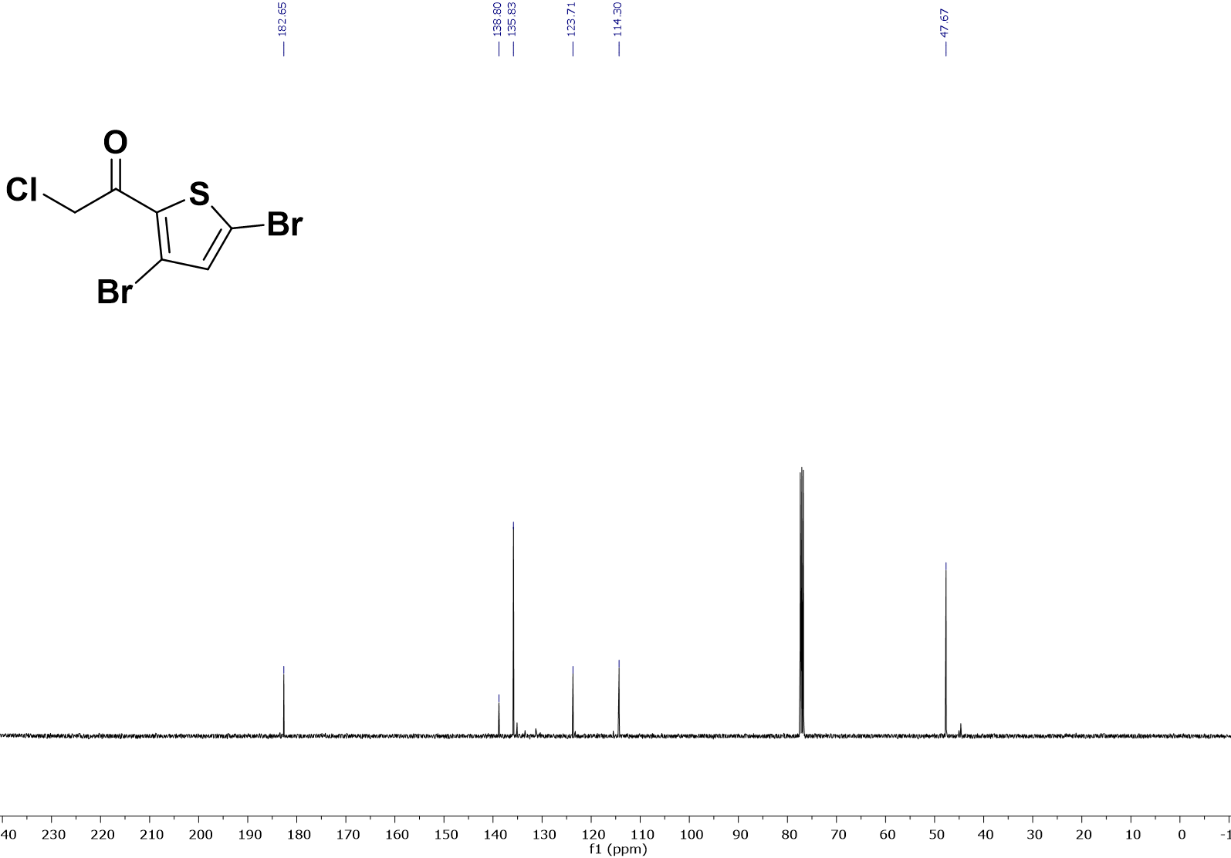


^13^C NMR of **2-chloro-1-(3,5-dibromothiophen-2-yl)ethan-1-one (1-SO-83)** in CDCl_3_


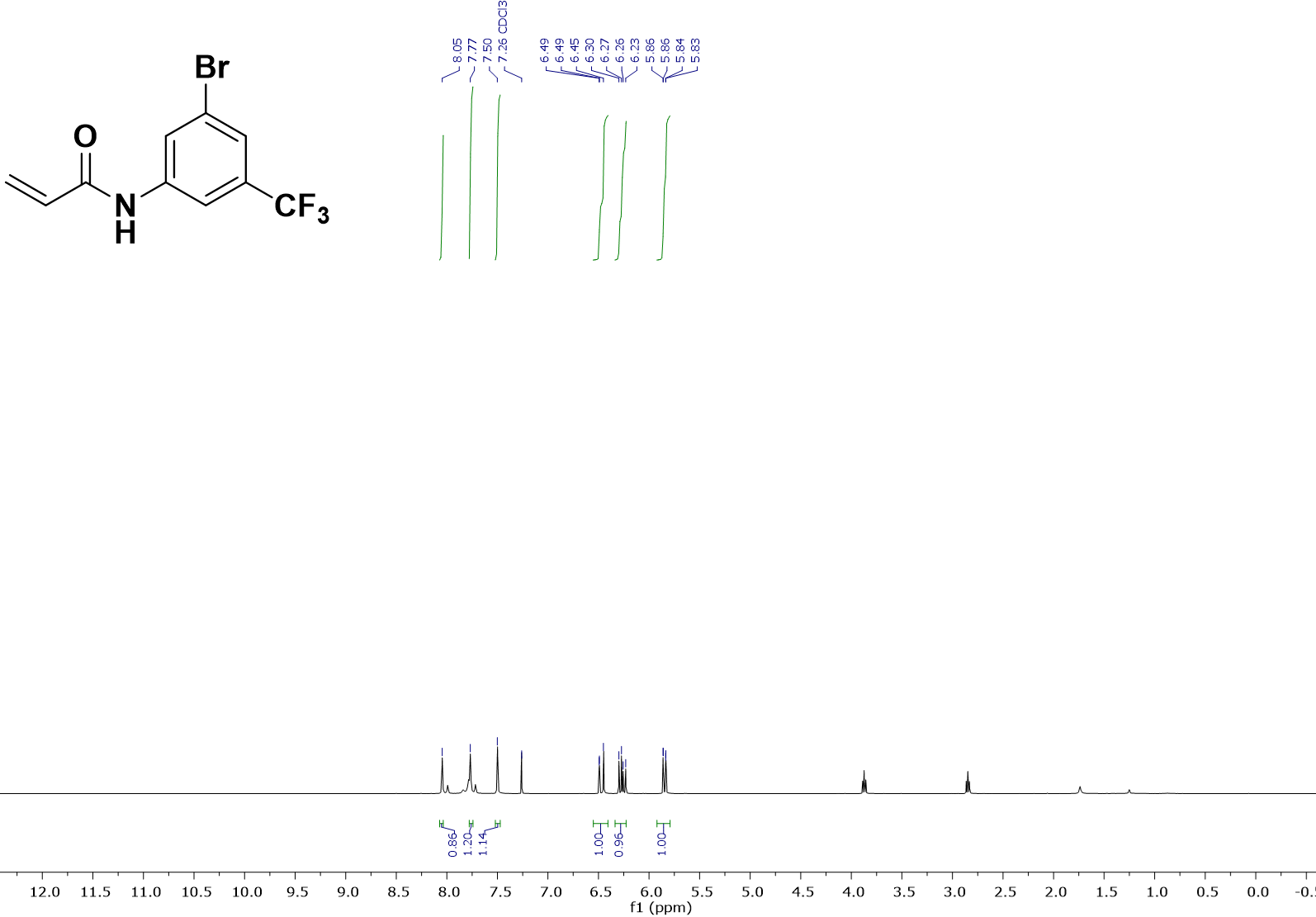


^1^H NMR of **N-(3-bromo-5-(trifluoromethyl)phenyl)acrylamide (1-SO-98)** in CDCl_3_


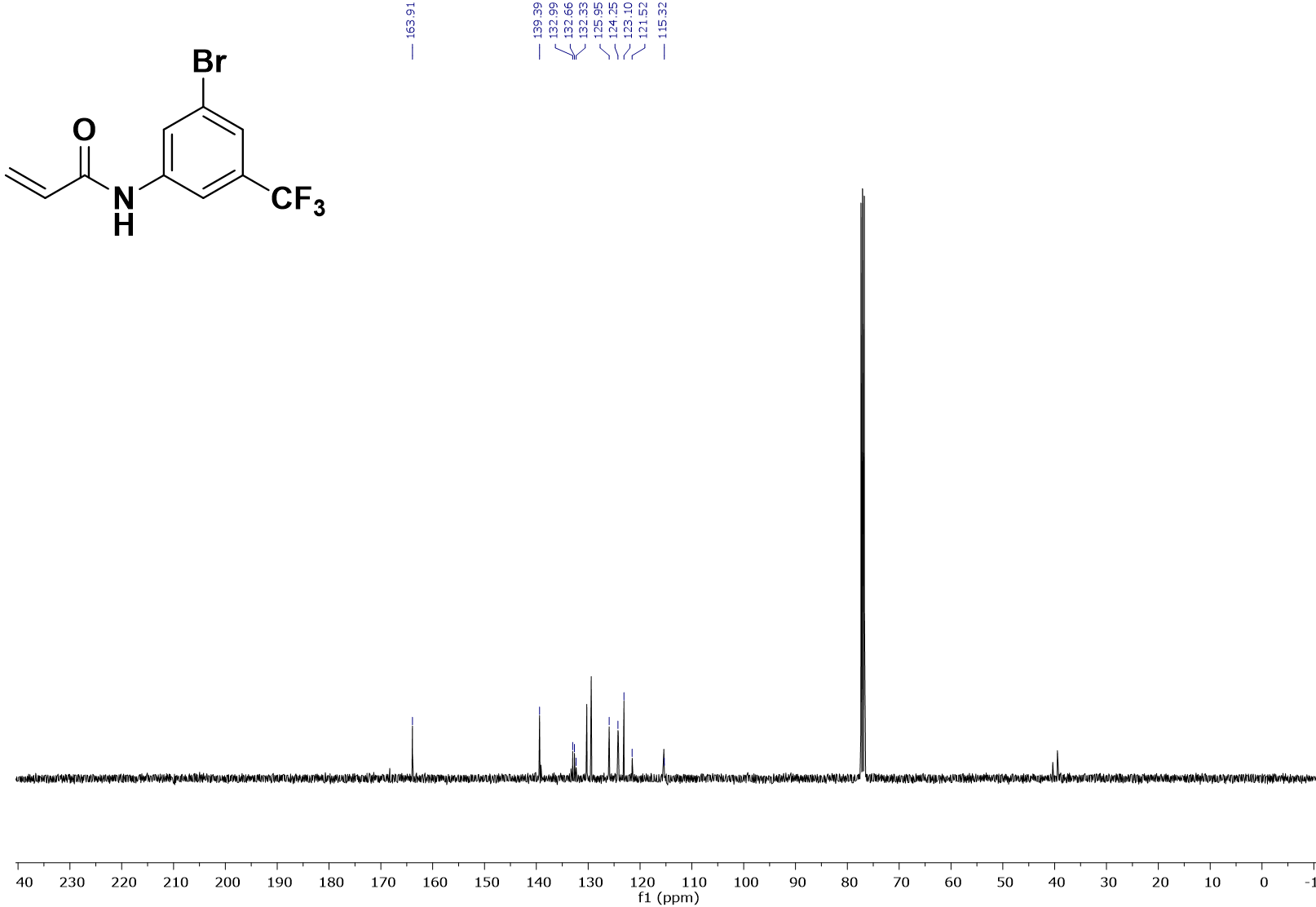


^13^C NMR of **N-(3-bromo-5-(trifluoromethyl)phenyl)acrylamide (1-SO-98)** in CDCl_3_


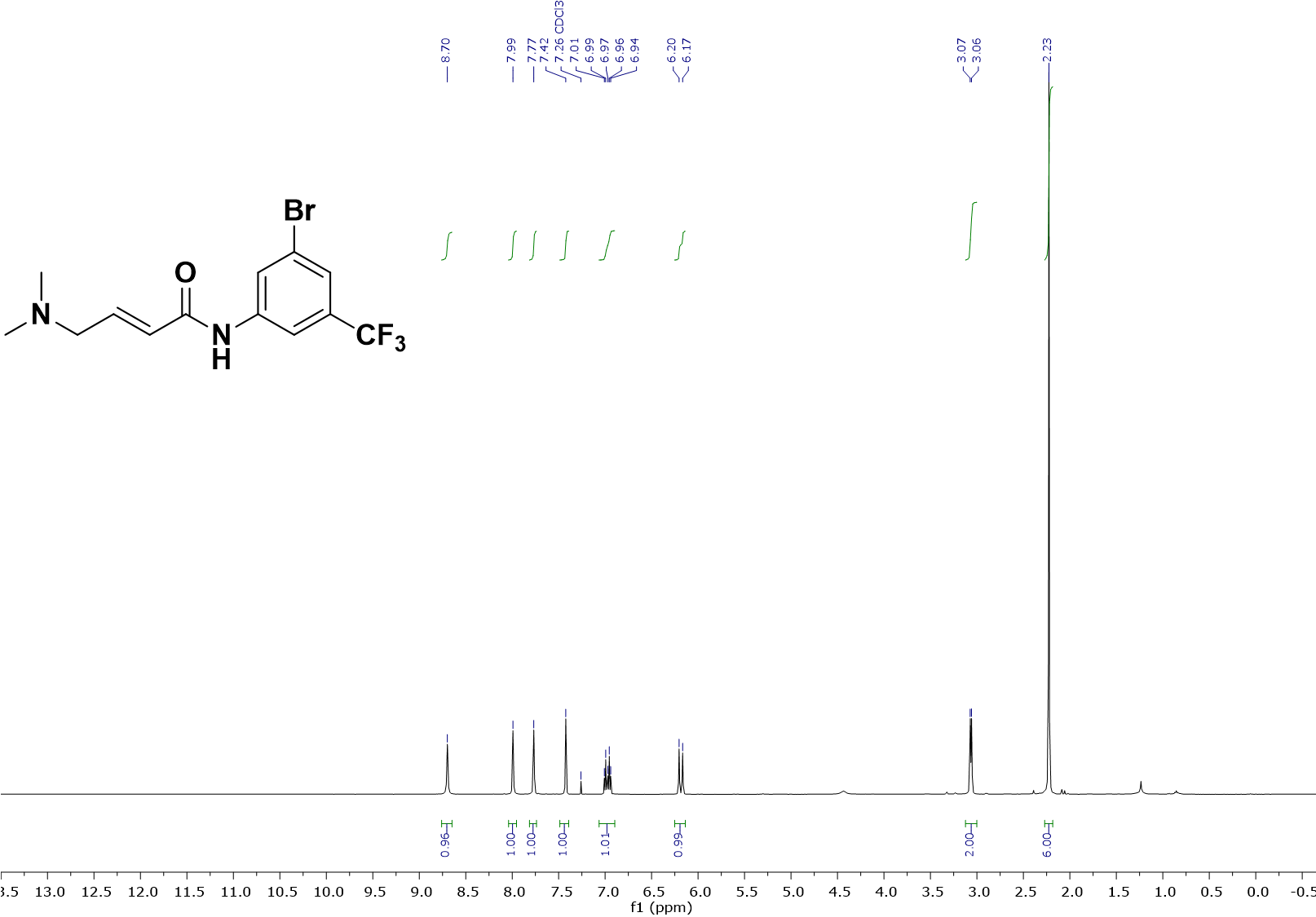


^1^H NMR of **(E)-N-(3-bromo-5-(trifluoromethyl)phenyl)-4-(dimethylamino)but-2-enamide (1-SO-176)** in CDCl_3_


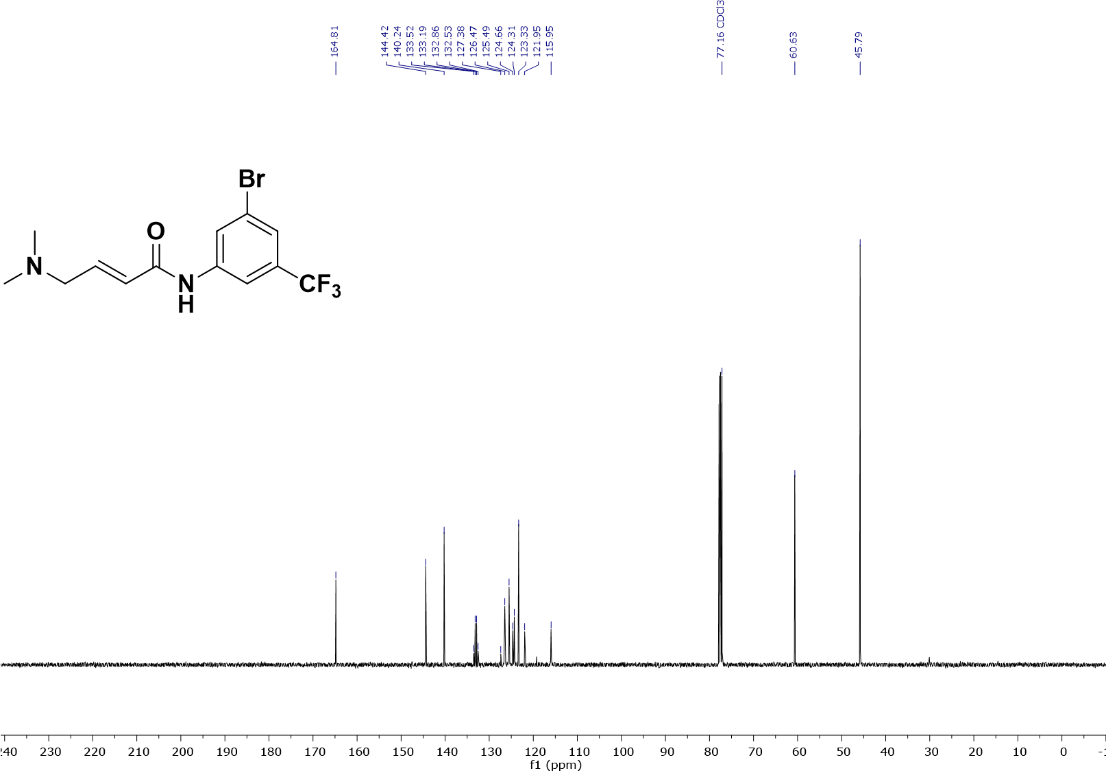


^13^C NMR of **(E)-N-(3-bromo-5-(trifluoromethyl)phenyl)-4-(dimethylamino)but-2-enamide (1-SO-176)** in CDCl_3_


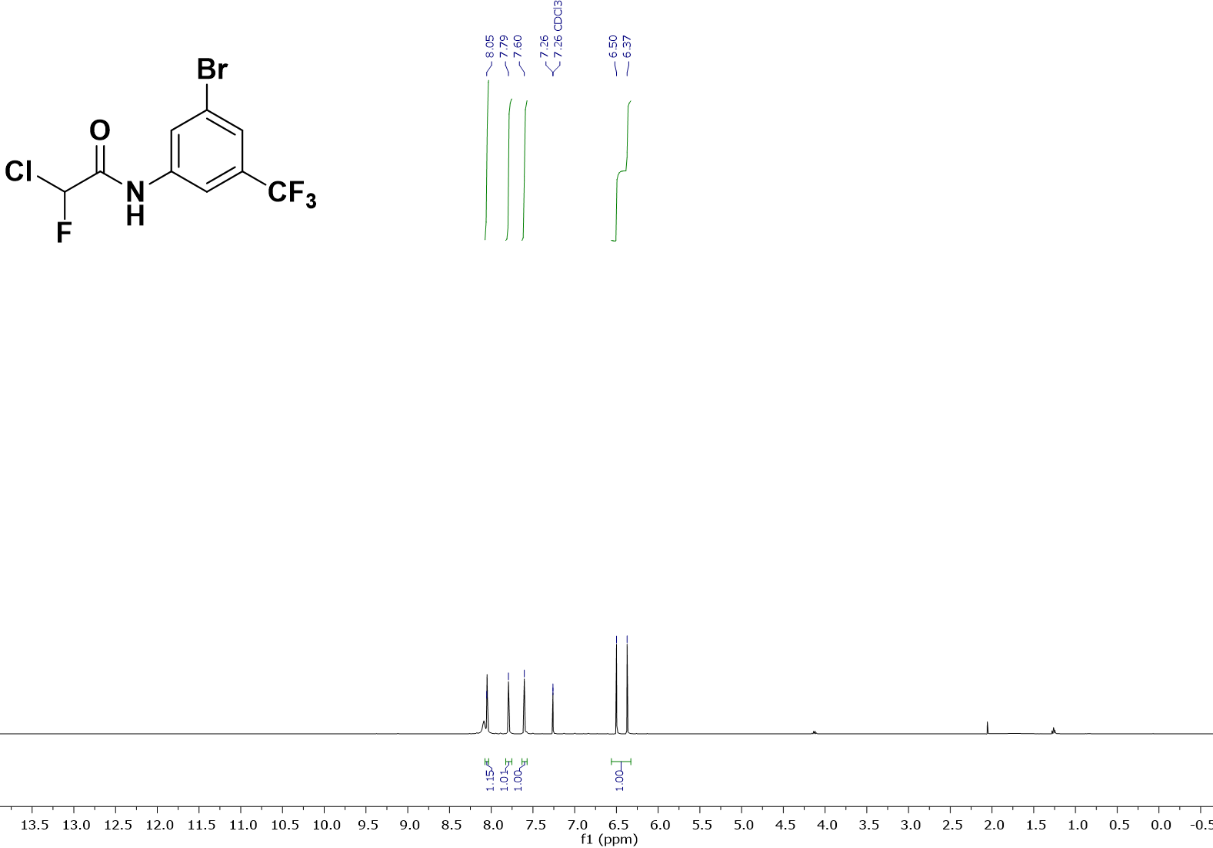


^1^H NMR of **N-(3-bromo-5-(trifluoromethyl)phenyl)-2-chloro-2-fluoroacetamide (2-SO-237)** in CDCl_3_


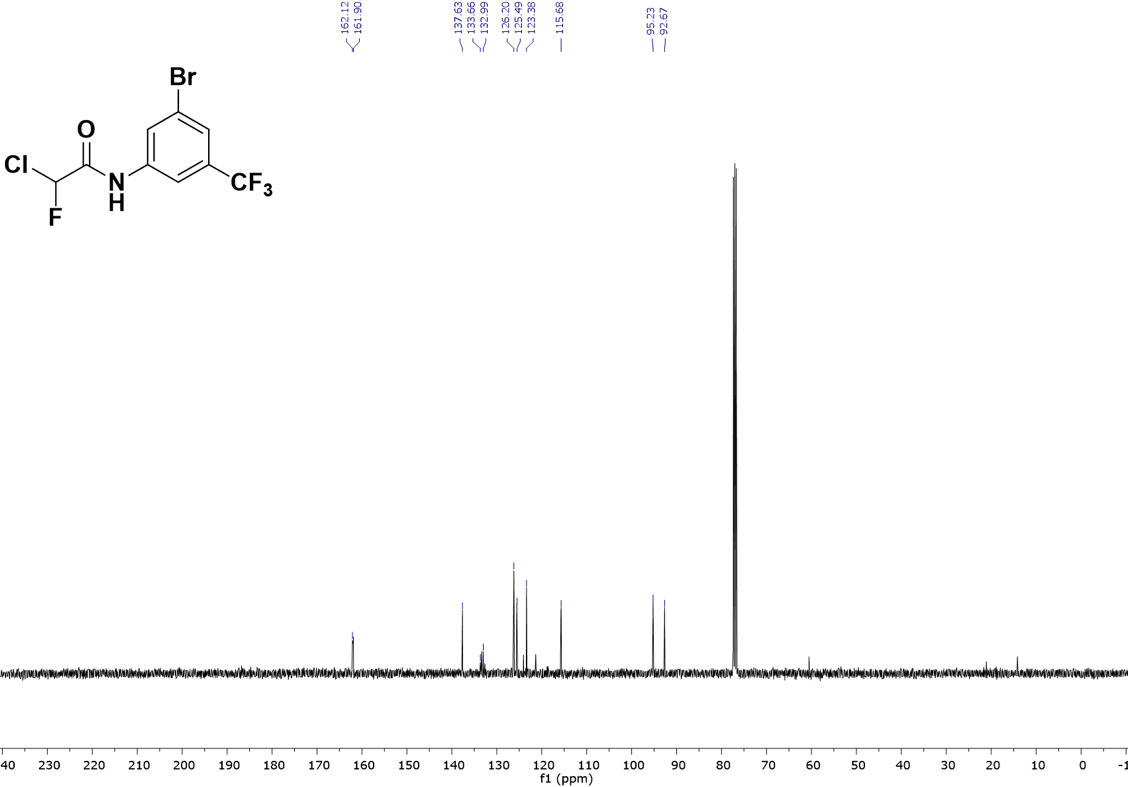


^13^C NMR of **N-(3-bromo-5-(trifluoromethyl)phenyl)-2-chloro-2-fluoroacetamide (2-SO-237)** in CDCl_3_


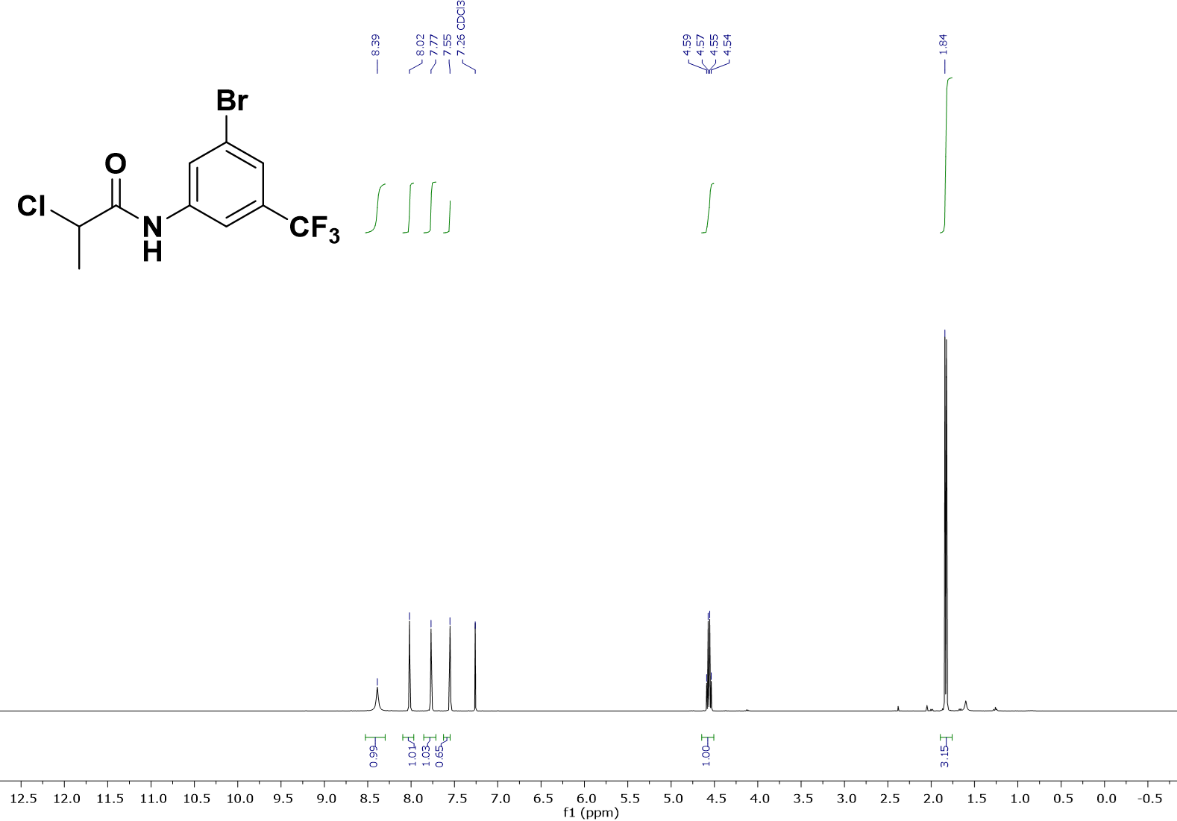


^1^H NMR of **N-(3-bromo-5-(trifluoromethyl)phenyl)-2-chloropropanamide (2-SO-238)** in CDCl_3_


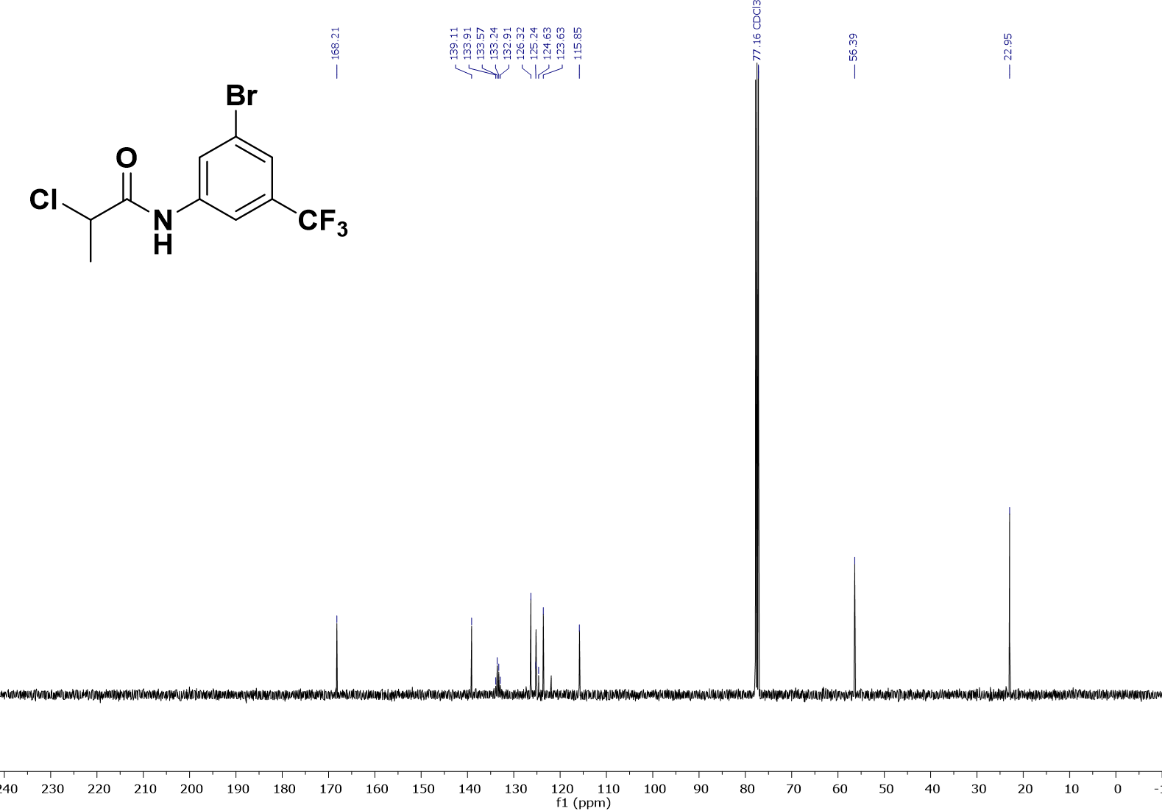


^13^C NMR of **N-(3-bromo-5-(trifluoromethyl)phenyl)-2-chloropropanamide (2-SO-238)** in CDCl_3_


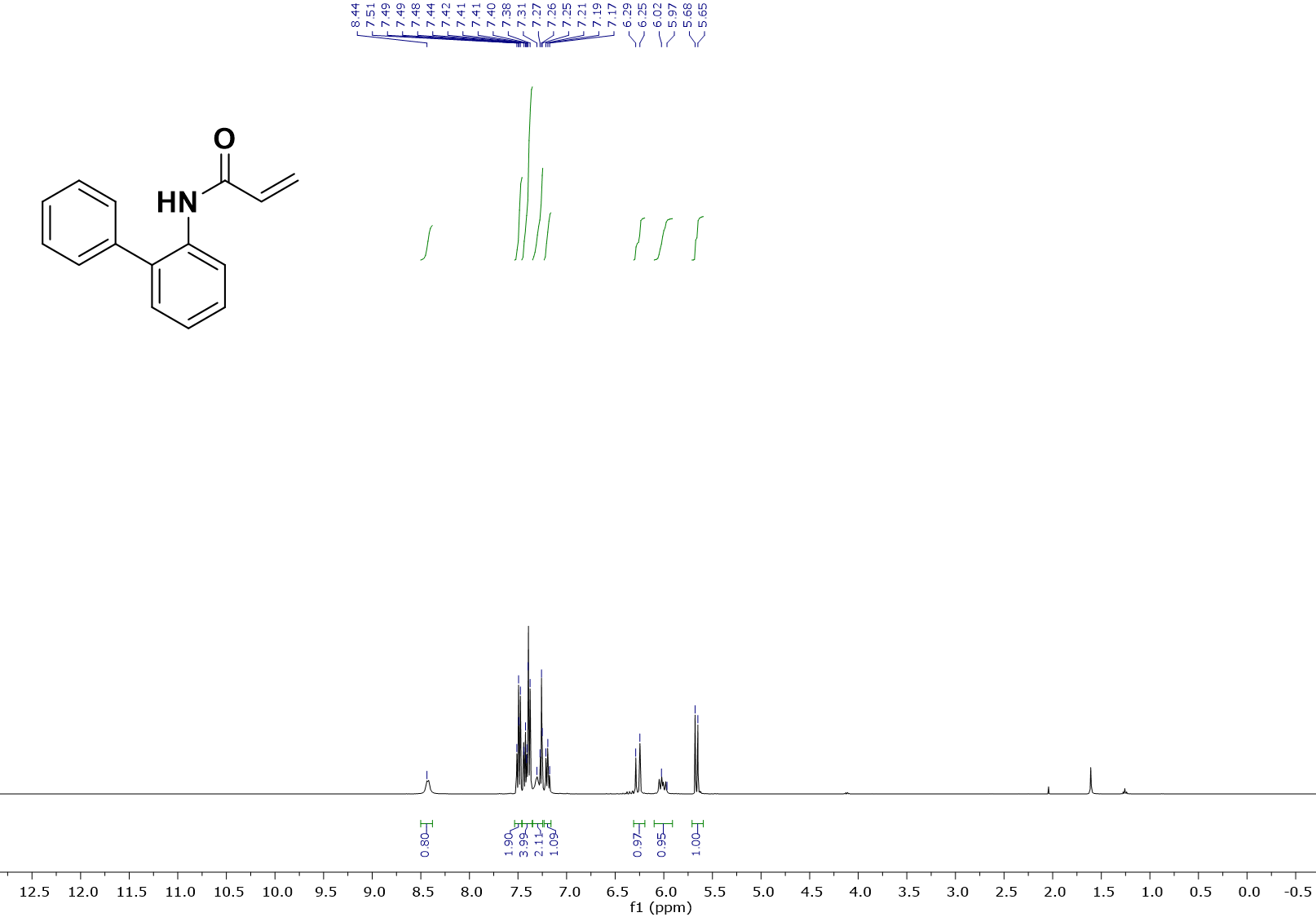


^1^H NMR of **1N-([1,1'-biphenyl]-2-yl)acrylamide (1-SO-96)** in CDCl_3_


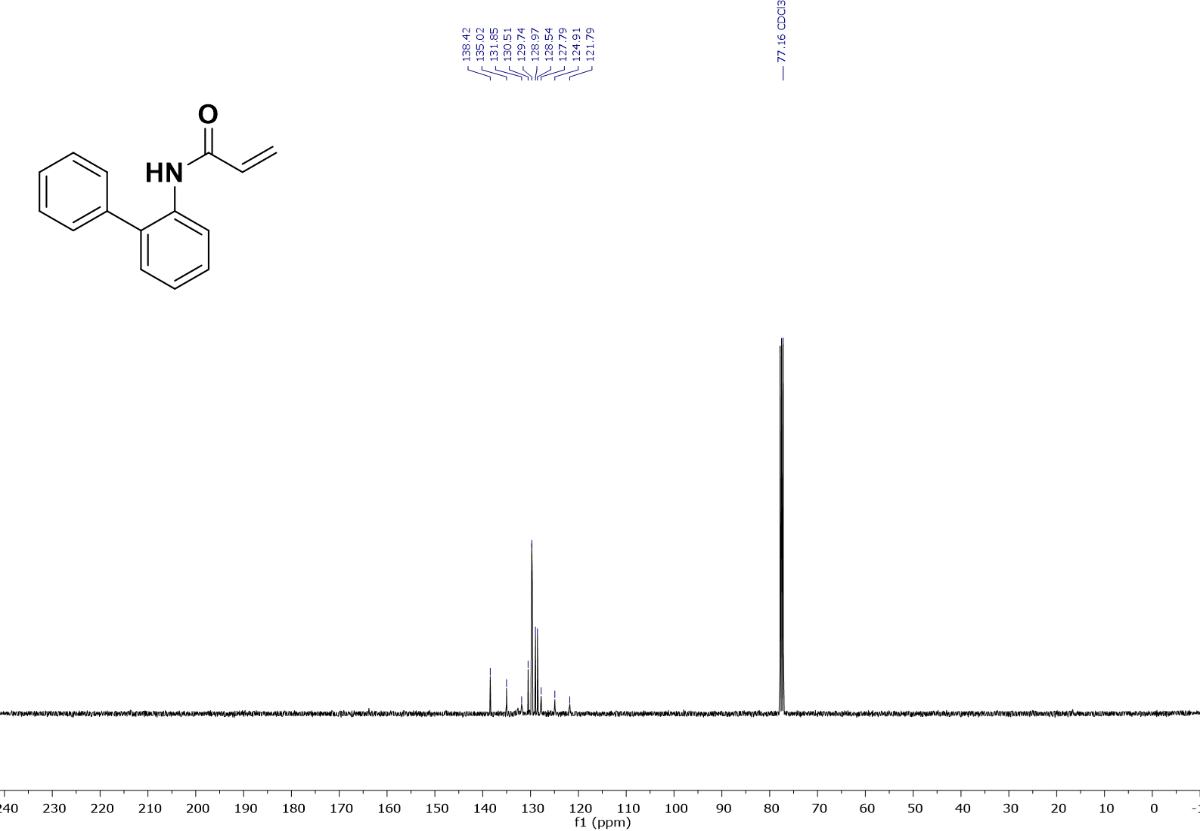


^13^C NMR of **N-([1,1'-biphenyl]-2-yl)acrylamide (1-SO-96)** in CDCl_3_


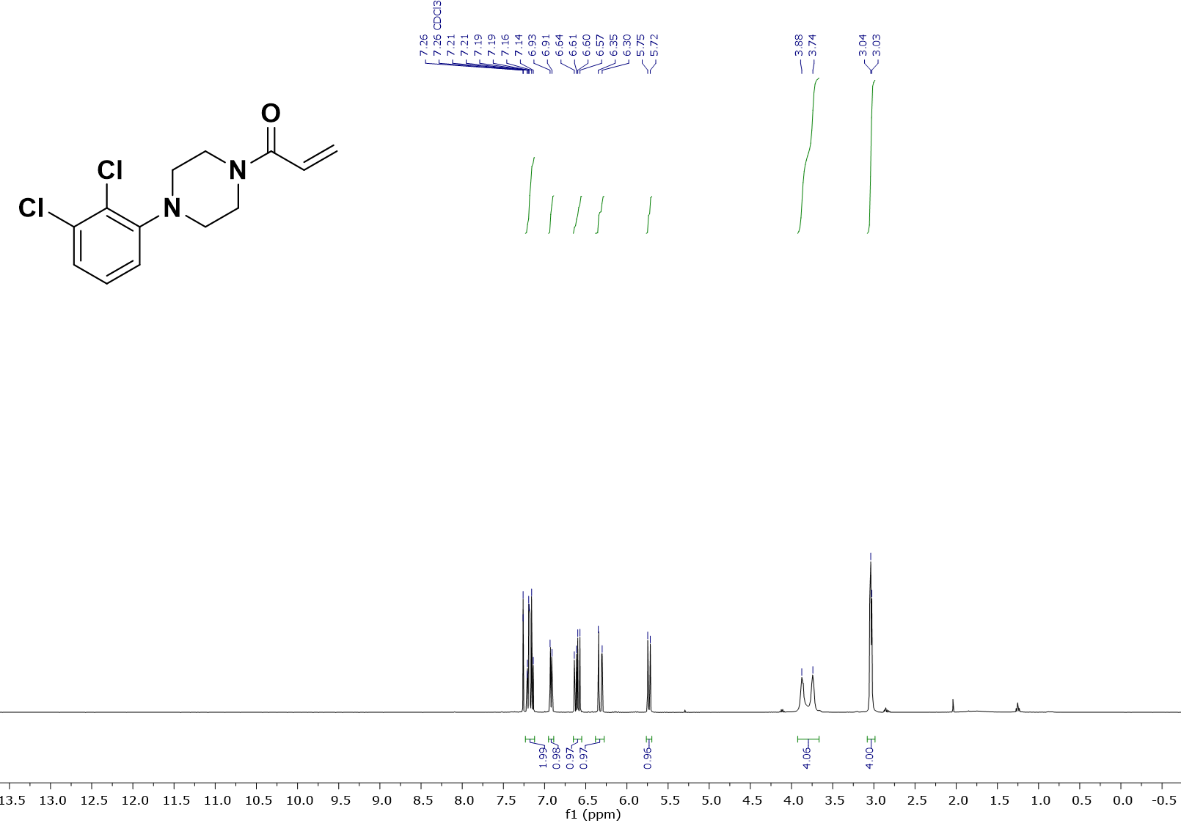


^1^H NMR of **1-(4-(2,3-dichlorophenyl)piperazin-1-yl)prop-2-en-1-one (1-SO-60)** in CDCl_3_


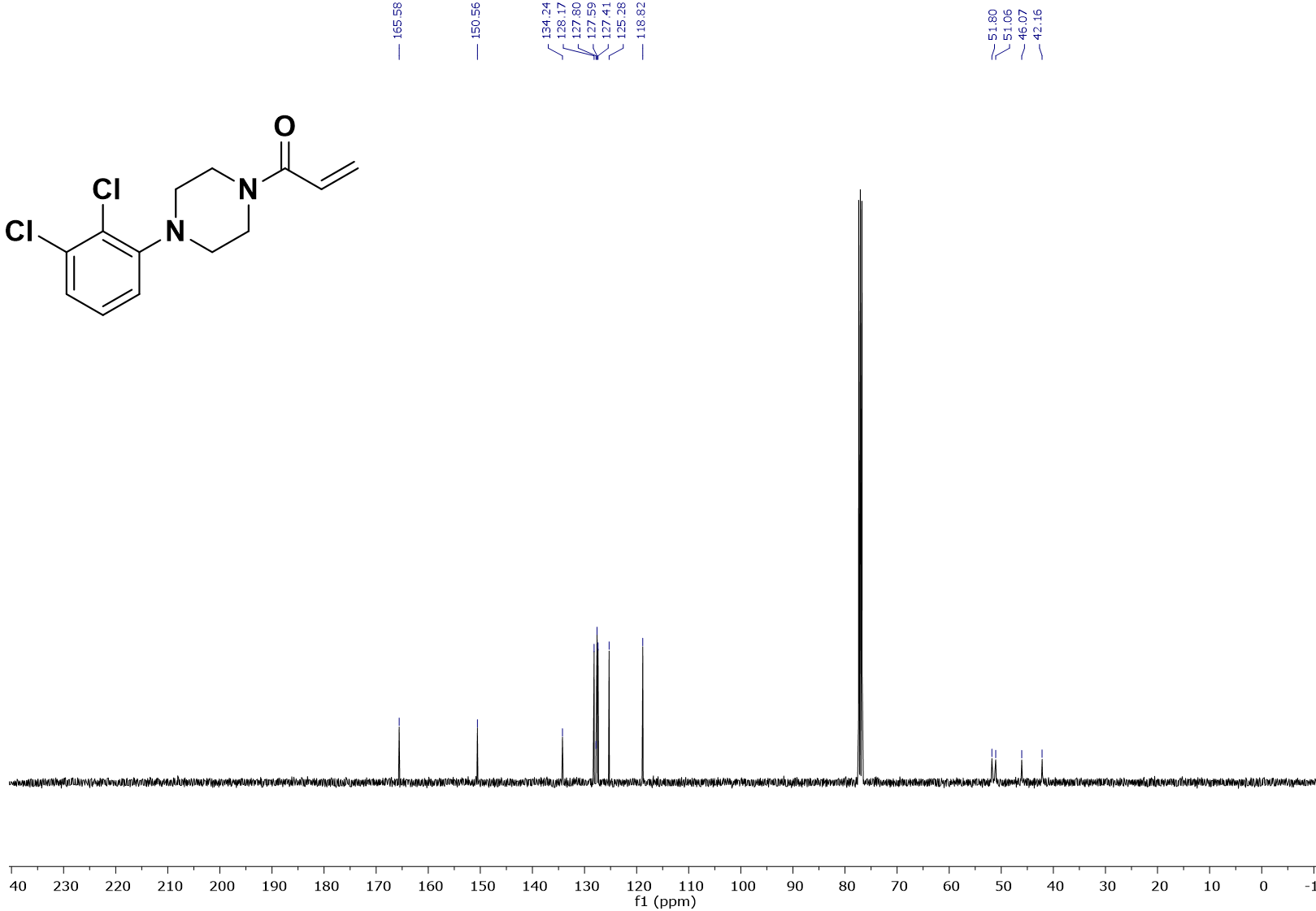


^13^C NMR of **1-(4-(2,3-dichlorophenyl)piperazin-1-yl)prop-2-en-1-one (1-SO-60)** in CDCl_3_


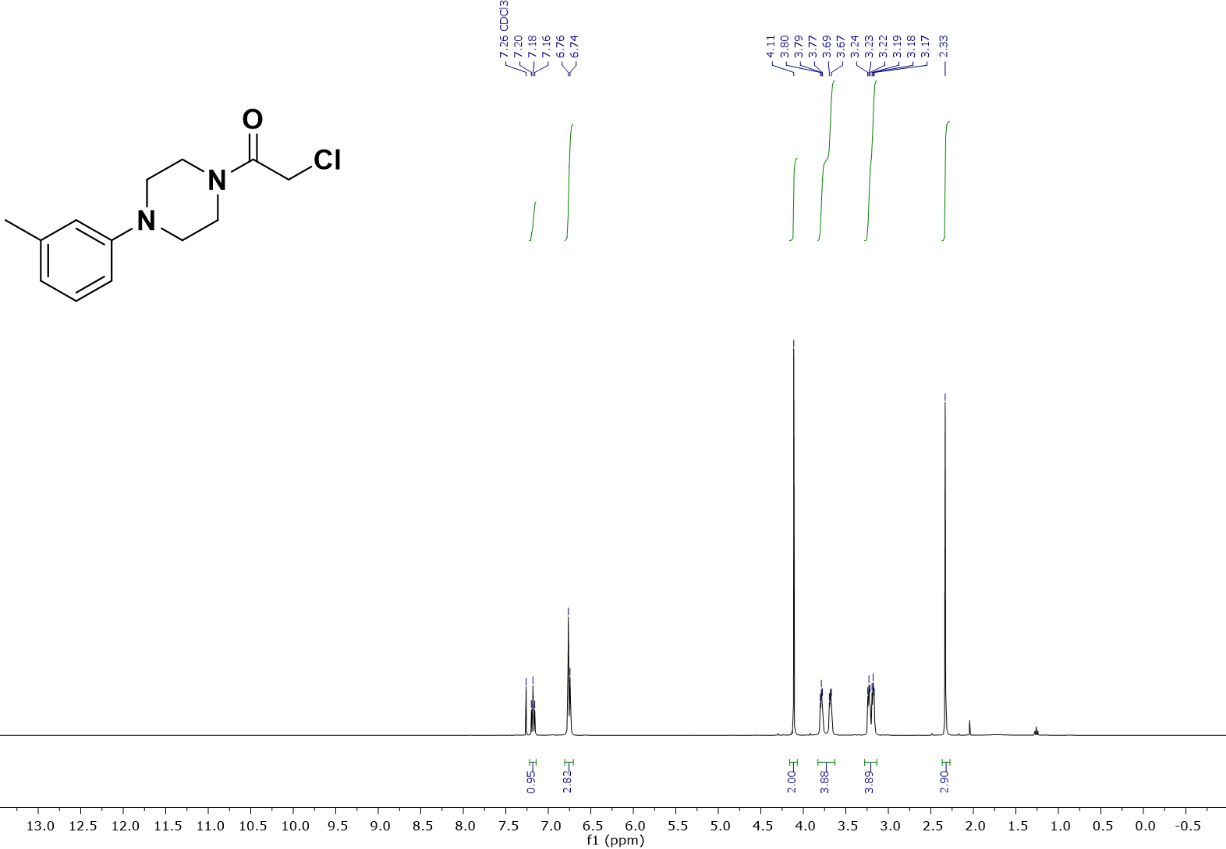


^1^H NMR of **2-chloro-1-(4-(m-tolyl)piperazin-1-yl)ethan-1-one (1-SO-68)** in CDCl_3_


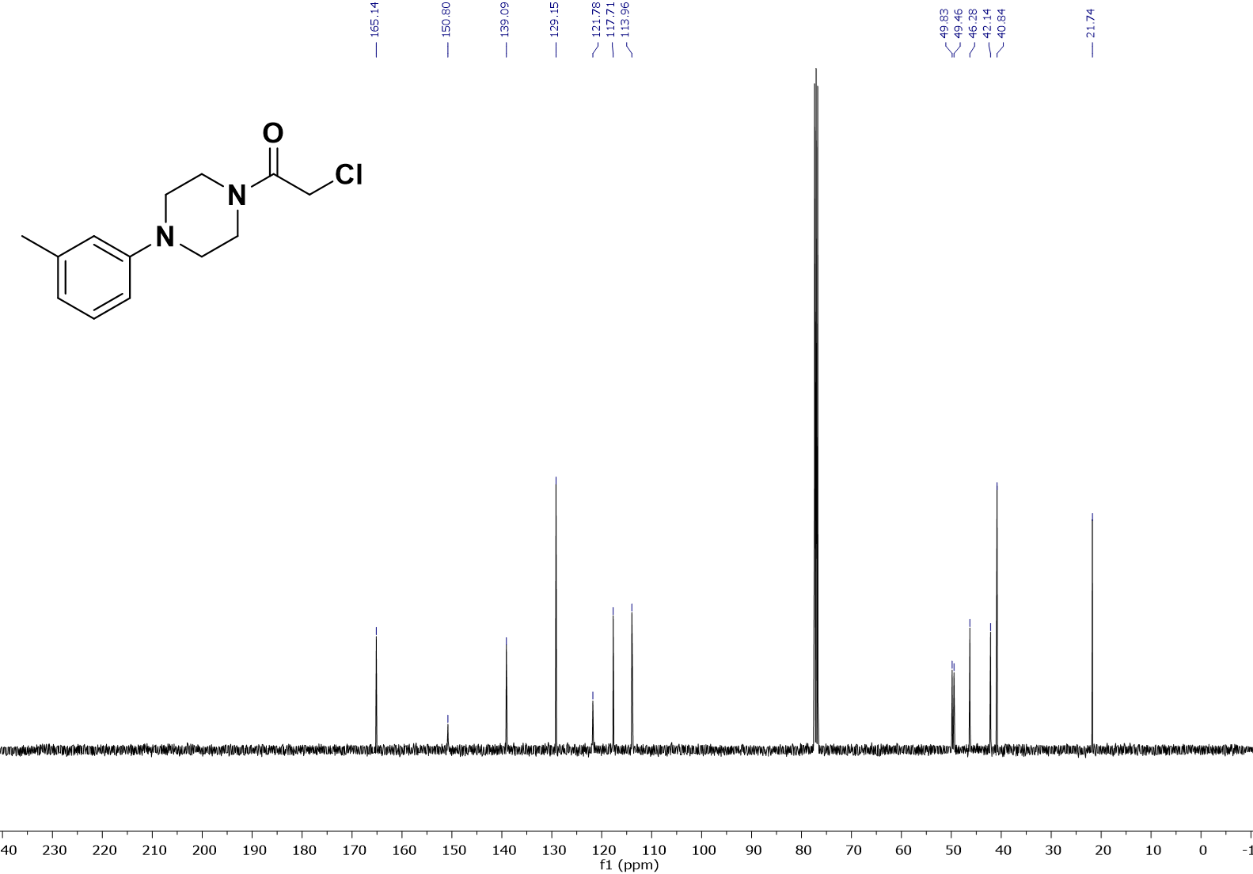


^13^C NMR of **2-chloro-1-(4-(m-tolyl)piperazin-1-yl)ethan-1-one (1-SO-68)** in CDCl_3_


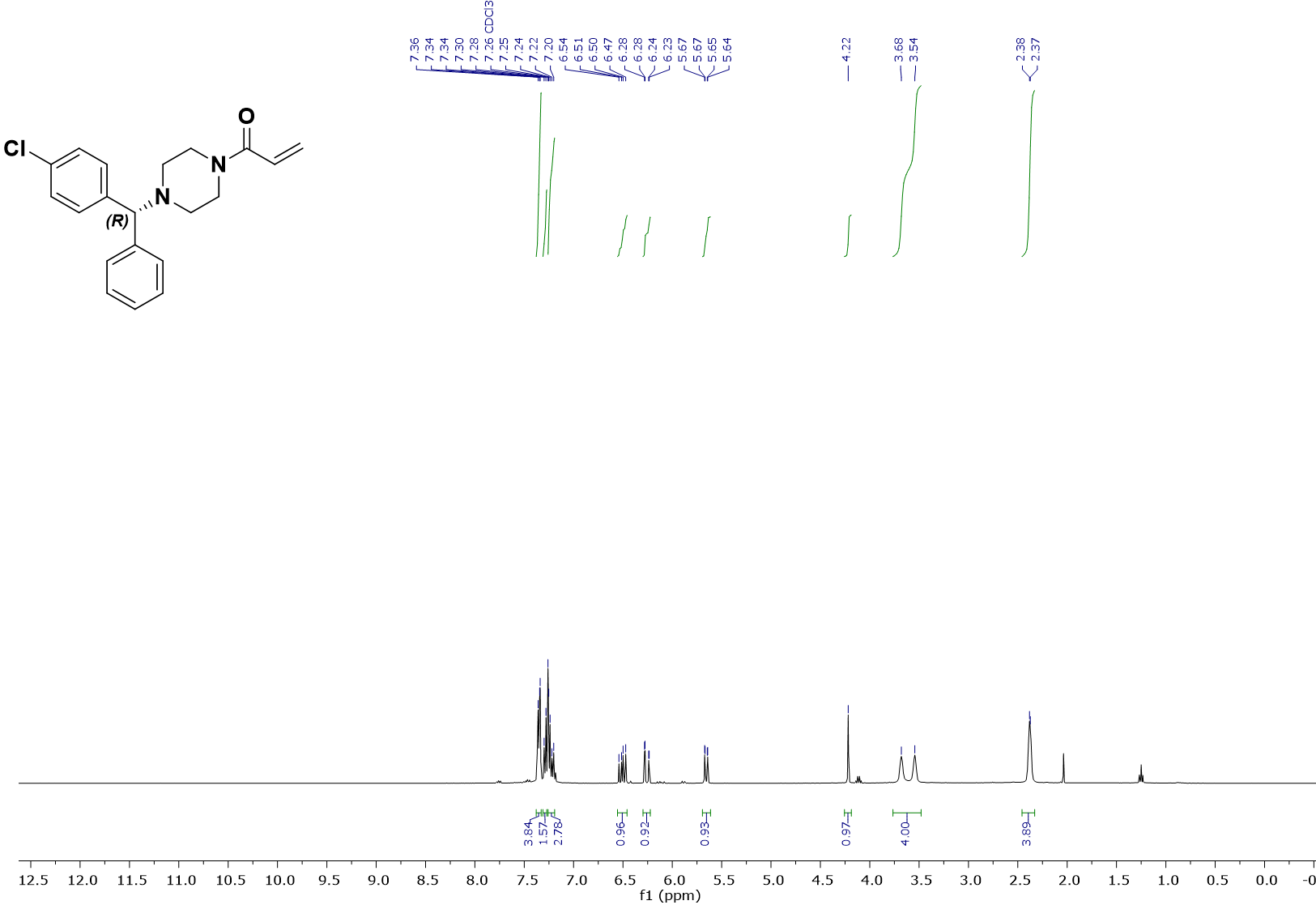


^1^H NMR of **(R)-1-(4-((4-chlorophenyl)(phenyl)methyl)piperazin-1-yl)prop-2-en-1-one (2-SO-253)** in CDCl_3_


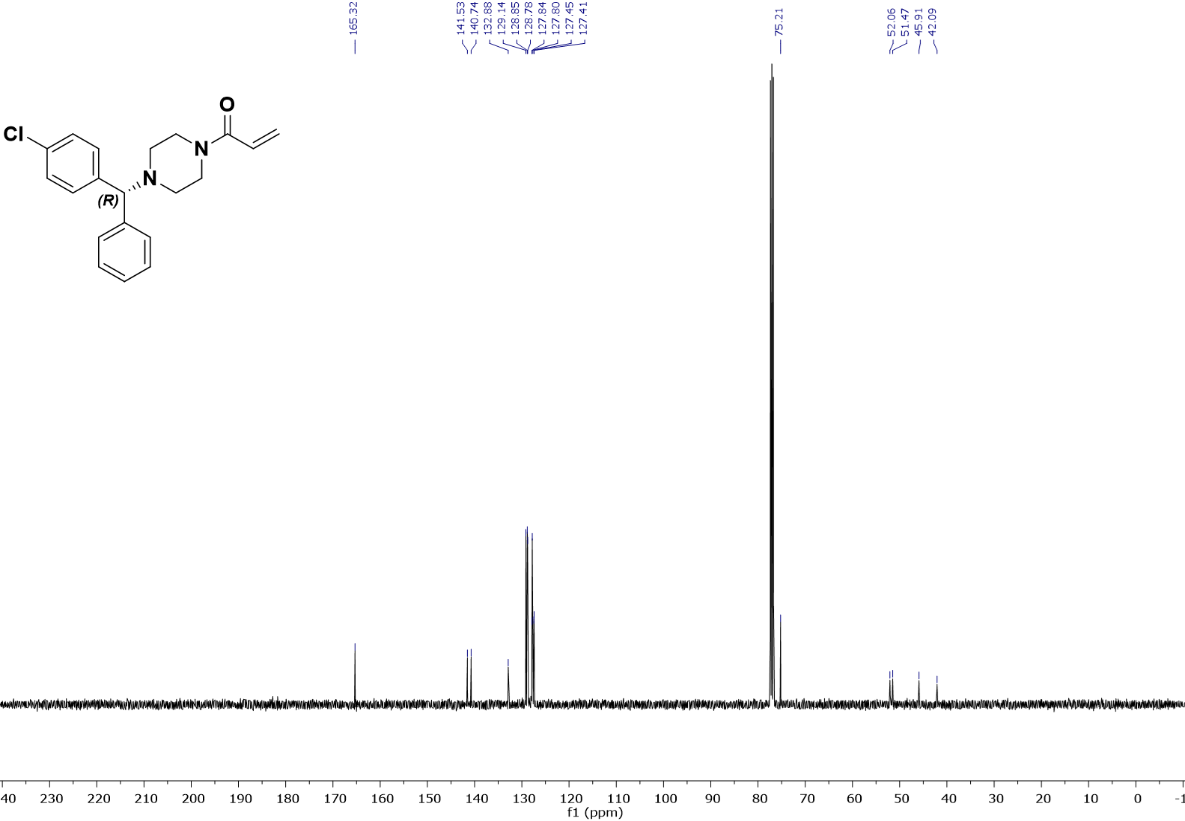


^13^C NMR of **(R)-1-(4-((4-chlorophenyl)(phenyl)methyl)piperazin-1-yl)prop-2-en-1-one (2-SO-253)**  in CDCl_3_

# References

[1.    Weerapana, E. *et al.* Quantitative reactivity profiling predicts functional cysteines in proteomes. *Nature* **468**, 790–795 (2010).](https://sciwheel.com/work/bibliography/1241563)

[2.    Yan, T. *et al.* Enhancing Cysteine Chemoproteomic Coverage through Systematic Assessment of Click Chemistry Product Fragmentation. *Anal. Chem.* **94**, 3800–3810 (2022).](https://sciwheel.com/work/bibliography/14615806)

[3.    Cao, J. *et al.* Multiplexed CuAAC Suzuki-Miyaura Labeling for Tandem Activity-Based Chemoproteomic Profiling. *Anal. Chem.* **93**, 2610–2618 (2021).](https://sciwheel.com/work/bibliography/11898890)

[4.    Boatner, L. M., Palafox, M. F., Schweppe, D. K. & Backus, K. M. CysDB: a human cysteine database based on experimental quantitative chemoproteomics. *Cell Chem. Biol.* **30**, 683-698.e3 (2023).](https://sciwheel.com/work/bibliography/14904677)

[5.    Yan, T. *et al.* SP3-FAIMS Chemoproteomics for High-Coverage Profiling of the Human Cysteinome*. *Chembiochem* **22**, 1841–1851 (2021).](https://sciwheel.com/work/bibliography/11898879)

[6.    Burton, N. R. *et al.* Solid-Phase Compatible Silane-Based Cleavable Linker Enables Custom Isobaric Quantitative Chemoproteomics. *J. Am. Chem. Soc.* **145**, 21303–21318 (2023).](https://sciwheel.com/work/bibliography/15549866)

[7.    Collins, D. M. *et al.* Preclinical Characteristics of the Irreversible Pan-HER Kinase Inhibitor Neratinib Compared with Lapatinib: Implications for the Treatment of HER2-Positive and HER2-Mutated Breast Cancer. *Cancers (Basel)* **11**, (2019).](https://sciwheel.com/work/bibliography/11109253)
